# Sequential Insertion of Alkynes, Alkenes, and CO into the Pd–C Bond of *ortho*-Palladated Primary Phenethylamines: from η^3^-Allyl Complexes and Enlarged Palladacycles to Functionalized Arylalkylamines

**DOI:** 10.1021/acs.organomet.0c00787

**Published:** 2021-01-29

**Authors:** José-Antonio García-López, María-José Oliva-Madrid, Delia Bautista, José Vicente, Isabel Saura-Llamas

**Affiliations:** † Grupo de Química Organometálica, Departamento de Química Inorgánica, Facultad de Química, Universidad de Murcia, E−30100 Murcia, Spain; ‡ ACTI, Universidad de Murcia, E−30100 Murcia, Spain

## Abstract

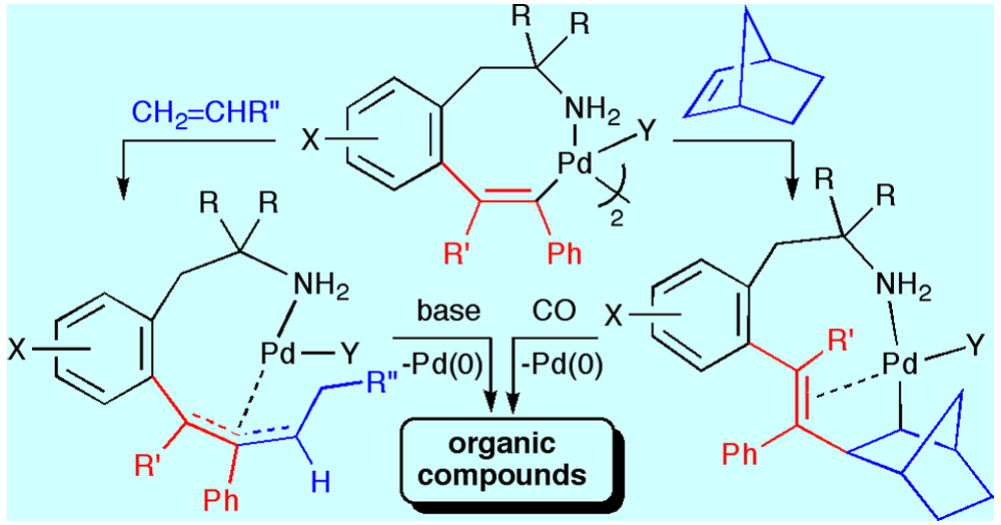

The eight-membered metallacycles arising from the insertion of 1 equiv of alkyne into the Pd–C bond of *ortho*-metalated homoveratrylamine and phentermine can further react with alkenes to give two different types of mononuclear complexes depending on the nature of the olefin. When terminal alkenes (styrene and ethyl acrylate) are used, a mixture of the *anti*/*syn* η^3^-allyl Pd(II) complexes are isolated, which evolve slowly to the *syn* isomers by heating the mixtures appropriately. These η^3^-allyl Pd(II) complexes do not react with CO or weak bases, but when they are treated with a strong base, such as KO^t^Bu, they afford Pd(0) and the functionalized starting phenethylamines containing a 1,3-butadienyl substituent in an *ortho* position. When 2-norbornene was used instead of terminal alkenes, the strained olefin inserts into the alkenyl Pd(II) complex to afford a 10-membered norbornyl palladium(II) complex, in which the new *C*,*N*-chelate ligand is coordinated to the metal through an additional double bond, occupying three coordination positions. The reactivity of these norbornyl complexes depends on the substituents on the inserted alkenyl fragment, and thus they can further react with (1) KO^t^Bu, to give Pd(0) and a tetrahydroisoquinoline nucleus containing a tricyclo[3.2.1]octyl ring, or (2) CO and TlOTf, to afford Pd(0) and amino acid derivatives or the corresponding lactones arising from an intramolecular Michael addition of the CO_2_H group to the α,β-unsaturated ester moiety. Crystal structures of every type of compound have been determined by X-ray diffraction studies.

## Introduction

Palladium is one of the most versatile transition metals in organic synthesis, given its well-known ability to catalyze the formation of C–C and C–heteroatom bonds.^1,2^ Nevertheless, the extraordinarily high variability of applications of palladium relies just on a few elementary steps inherent to its reactivity, and among them, the migratory insertion reaction of an unsaturated ligand into a Pd–C bond stands out.^3−5^ Thus, a myriad of methods to functionalize alkenes,^3,6^ alkynes,^7^ or allenes^8^ through Pd catalysis have been reported so far.

**Scheme 1:**
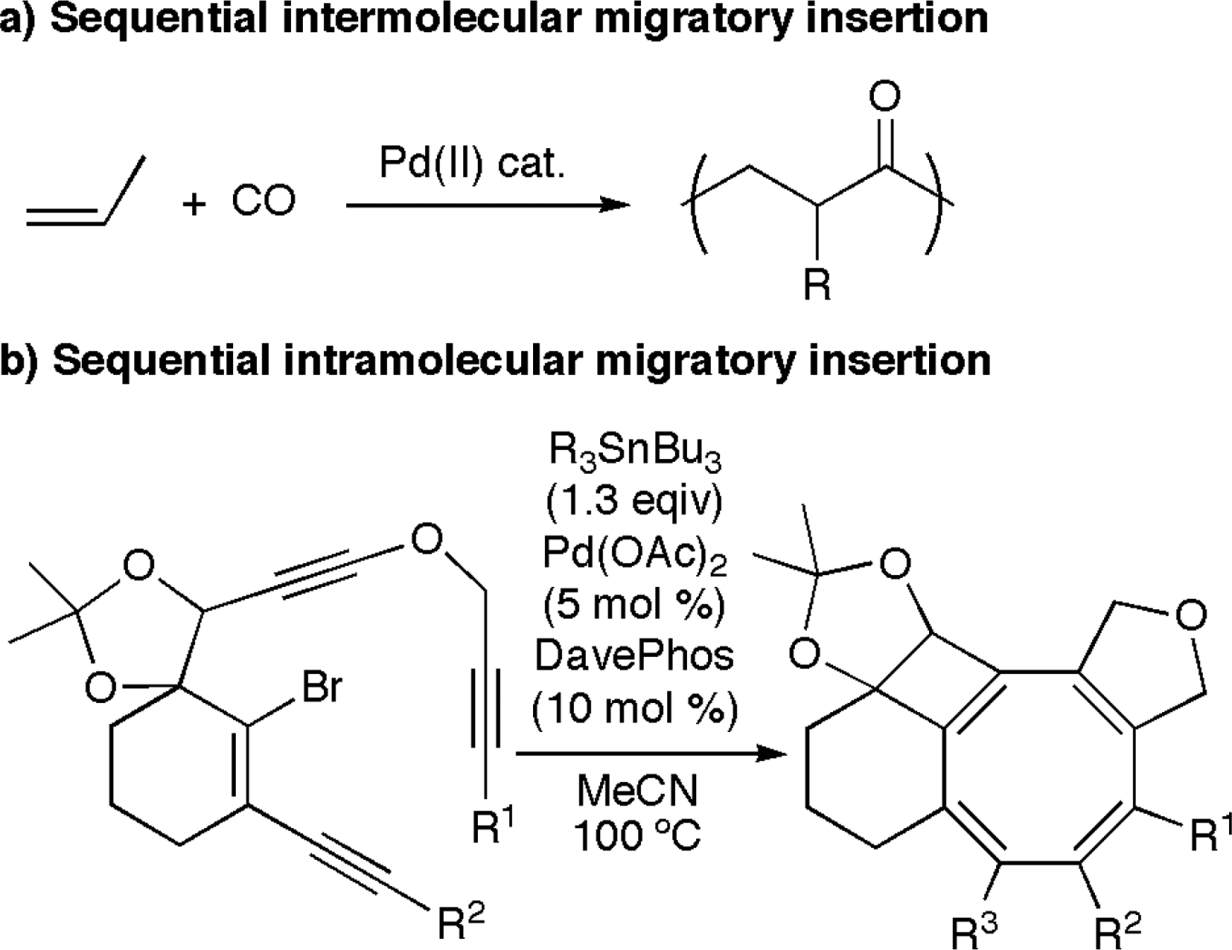
Examples of Reactions Involving Sequential Migratory Insertion into Pd–C Bonds^13,18c^

Multicomponent reactions, where several reagents assemble sequentially giving rise to complex structures, are valuable protocols, since they represent an effective modular approach to green organic synthesis.^9^ Nevertheless, transition-metal-catalyzed processes involving the use of several unsaturated coupling partners in the reaction mixture are challenging fields due to the difficulty in controlling the order of reactivity: that is, the control of regioselectivity of the migratory insertion sequence for the different unsaturated reagents.^10−12^ Some successful examples of these types of reactions are the copolymerization of alkenes and CO (Scheme 1a)^13^ and the copolymerization of olefins with and without polar groups,^14^ among others.^15−17^ Moreover, impressive progress has been made on intramolecular cascade reactions where different unsaturated moieties are involved (Scheme 1b).^5,18^ In the latter case, however, the selectivity is usually determined by the structure of the substrate itself.

While catalytic conditions make difficult the aforementioned control of the selectivity, stoichiometric reactions can offer the advantage of stepwise insertion paths for the synthesis of valuable organic products.^19−23^

**Scheme 2:**
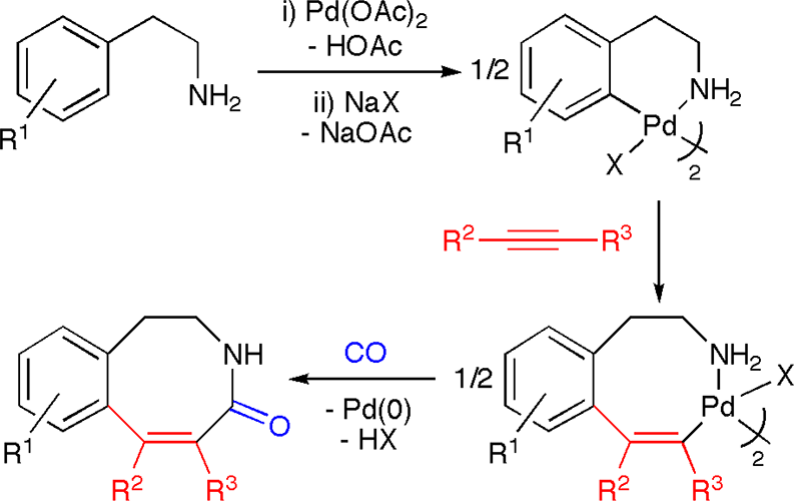
Synthesis of Benzazocinones through the Sequential Insertion of Alkynes and CO into the Pd–C Bond of Six-Membered Palladacycles

Our research group has previously reported the functionalization of primary phenethylamines through the isolation of *ortho*-palladated intermediates.^24−26^ These types of arylalkylamines constitute the core of many relevant biomolecules, such as neurotransmitters (i.e., dopamine), amino acids (i.e., phenylalanine), or marketed psychoactive drugs. The catalytic functionalization of these scaffolds has proven challenging, due to the strong coordination ability of the primary amine group to Pd(II).^27^ Hence, the study of the stoichiometric functionalization of primary phenethylamines can provide alternative routes to modify these interesting molecules. For instance, the sequential insertion of alkynes and CO or of alkynes and isocyanides into *ortho*-palladated derivatives allowed us to synthesize medium-sized rings such as eight-membered benzazocinones (Scheme 2).^20,21^

**Chart 1:**
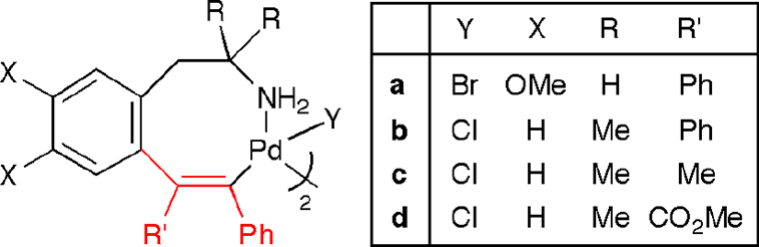
Previously Reported Eight-Membered Alkenyl Palladacycles Used as Starting Materials

We have previously reported the synthesis and isolation of stable eight-membered *C*,*N*-palladacycles arising from the insertion of one molecule of alkyne into the Pd–C bond of *ortho*-palladated phentermine and homoveratrylamine (Chart 1).^20^ We present here the results of a study on the insertion of alkenes and alkenes/CO into these eight-membered alkenyl palladacycles. We have studied the new organometallic species generated upon each insertion, as well as the final functionalized organic products, formed through depalladation of the final organopalladium compounds. The overall processes rendering the final organopalladium complexes involve the sequential insertion of (a) alkynes and alkenes or (b) alkynes, alkenes, and CO into the Pd–C bond of the *ortho*-metalated derivatives from the primary phenethylamines.

## Results and Discussion

### Insertion of Styrene or Ethyl Acrylate into the Pd–C Bond

#### Synthesis and Structure of η^3^-Allyl Palladium(II) Complexes

**Scheme 3:**
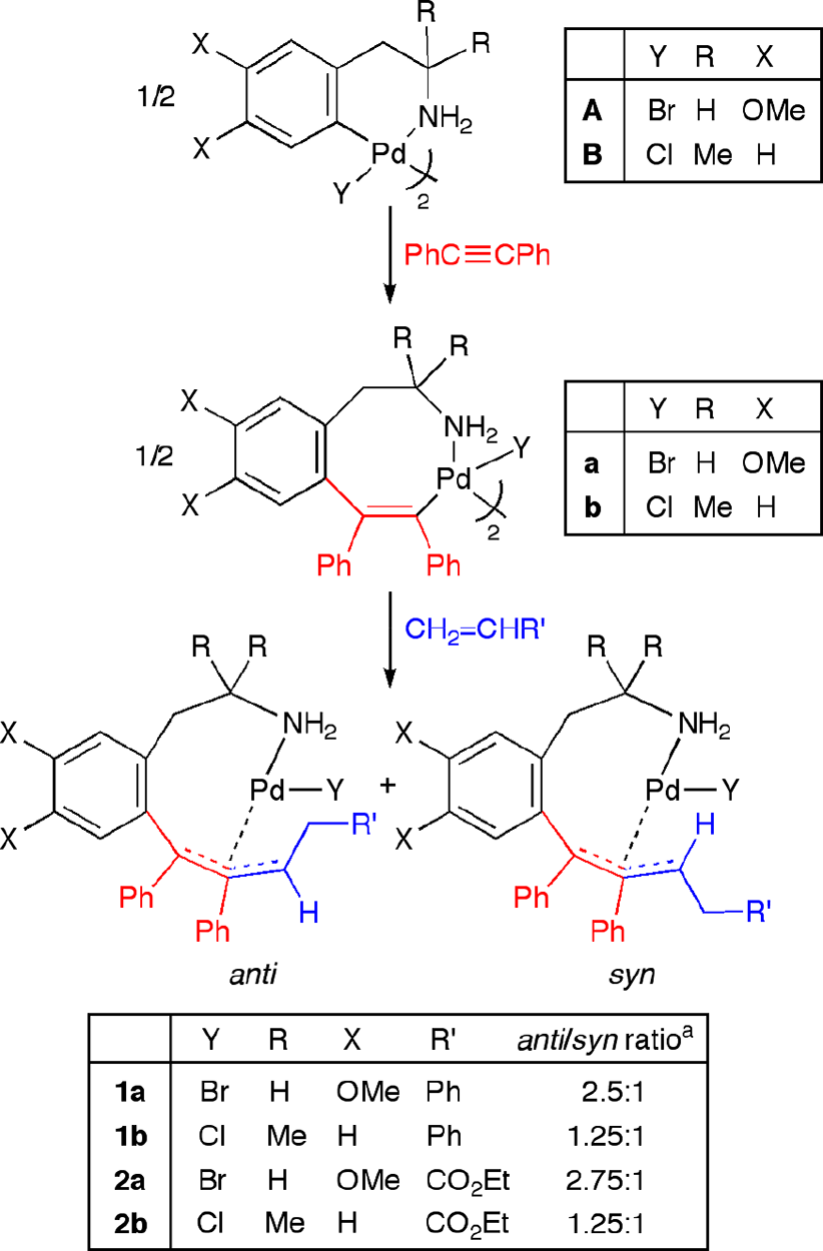
Reactions of Eight-Membered Alkenyl Palladacycles with Terminal Alkenes The ratios correspond to the isolated solids and were estimated by the integrals of the CH allylic signals of both isomers in the ^1^H NMR spectra of the mixtures.

The reaction of the previously described alkenyl palladacycle **a** or **b**, arising from the monoinsertion of diphenylacetylene into the *ortho*-palladated homoveratrylamine and phentermine derivative **A** or **B**, with 2 equiv of CH_2_=CHR′ (molar ratio Pd/alkene = 1/1; R = Ph (**1**), CO_2_Et (**2**)) in CH_2_Cl_2_ at room temperature (16 h) gave a mixture of two complexes, which were identified as the *anti* and *syn* isomers of the η^3^-allyl Pd(II) complex **1** or **2** (Scheme 3).

**Figure 1:**
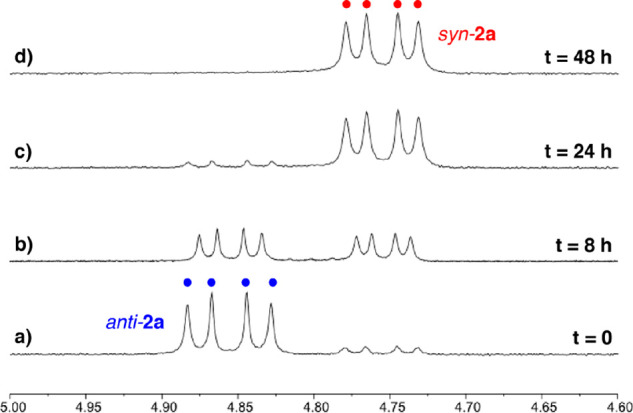
^1^H NMR spectra (4.6–5.0 ppm) of complex **2a** (CDCl_3_): (a) *anti*-enriched mixture of complex **2a** at room temperature (300 MHz) and (b) after heating at 60 °C for 8 h (400 MHz), (c) 24 h (300 MHz) and (d) 48 h (300 MHz).

After workup, a mixture of η^3^-allyl Pd(II) complexes *anti*-/*syn*-**1a** (2.5/1) or *anti-*/*syn*-**2a** (5/1) was isolated. Attempts to separate both isomers by fractional crystallization were unsuccessful. However, single crystals of *anti*-**1a** or *anti*-**2a**, suitable for X-ray diffraction studies, were obtained by slow diffusion of *n*-pentane into a solution of the mixture of *anti-*/*syn*-**1a** or *anti-*/*syn*-**2a** in CH_2_Cl_2_. The ratio between isomers, for both **1a** and **2a**, practically did not change after 48 h in CDCl_3_ solution at room temperature (by ^1^H NMR). However, the conversion to the *syn* isomers was complete after heating CDCl_3_ solutions of the mixtures at 60 °C for 10 days (*syn*-**1a**) or 48 h (*syn*-**2a**; by ^1^H NMR; Figure 1 and Table 1).

**Table 1:**
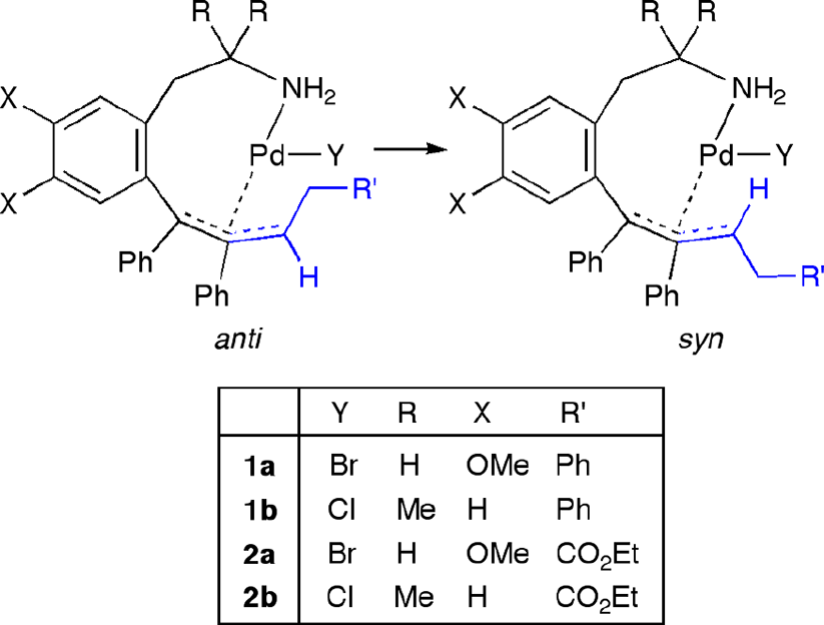
Isomerization Ratios for *anti-*/*syn*-**1** and *anti*-/*syn*-**2**

		*anti*/*syn* ratio			
temperature	time (h)	**1a**	**1b**	**2a**	**2b**
RT	0	2.5/1	2.5/1	5/1	1.25/1
	12	2.5/1		4.2/1	
	16				1.25/1
	24	2.5/1		4.2/1	
	48	2.5/1	2/1	3.3/1	1.25/1
					
60 °C	0	2.5/1	2.5/1	5/1	1.25/1
	8	1.7/1		1/1	
	12				1/5
	24	1.1/1		1/10	
	48	1/1.25	1/3.3	0/1	1/20
	120		1/10		
	240	0/1			

Enriched mixtures of both *anti* and *syn* isomers of **1b** and **2b** could be obtained due to the higher solubility of one of the two isomers in Et_2_O. When a solution of the mixture *anti-*/*syn*-**1b** or *anti-*/*syn*-**2b** (**1b**, ratio 1.25/1, 0.161 mmol; **2b**, ratio 1.25/1, 0.260 mmol) in CH_2_Cl_2_ (**1b**, 3 mL; **2b**, 2 mL) was treated with Et_2_O (15 mL), a mixture enriched in the *anti* (**1b**) or *syn* (**2b**) isomer precipitated (ratio *anti*/*syn*: **1b**, 3/1; **2b**: 1/3). When the mother liquors were concentrated (**1b**: 3 mL; **2b**: 5 mL) and treated with *n*-pentane (**1b**, 20 mL) or cooled to 0 °C in an ice bath (**2b**), a mixture enriched in the *syn* (**1b**) or *anti* (**2b**) isomer precipitated (ratio *anti*/*syn*: **1b**, 1/3; **2b**: 3/1). Almost spectroscopically pure samples of *anti*- and *syn*-**1b** and *anti*- and *syn*-**2b** could be obtained by growing single crystals from the enriched mixtures.

**Scheme 4:**
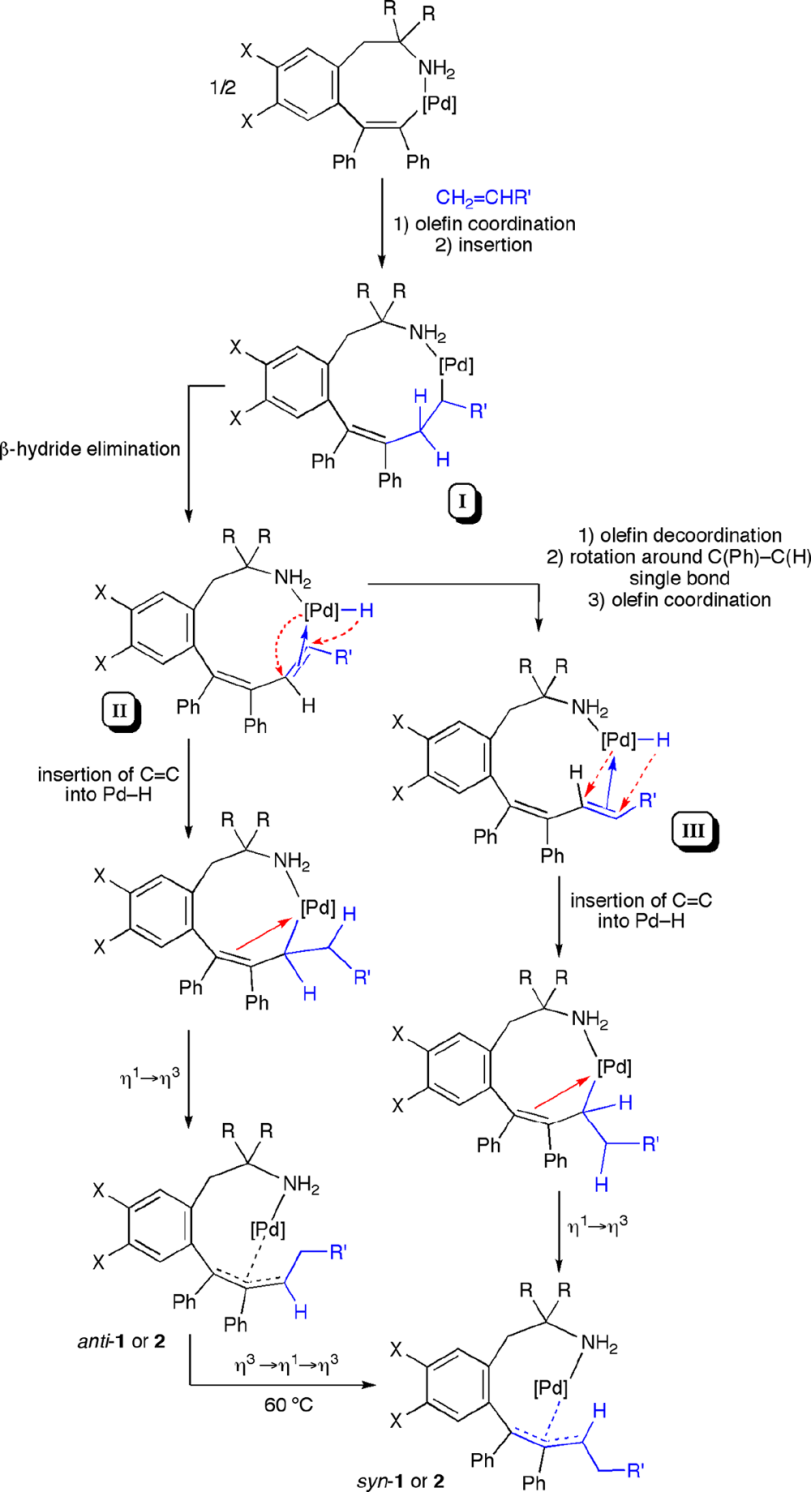
Formation of *anti* and *syn* Isomers of η^3^-Allyl Pd(II) Complexes

Since *anti*-**1** and *anti*-**2** complexes practically did not isomerize to the *syn*-**1** and *syn*-**2** derivatives at room temperature (Table 1), both isomers should be formed during the reaction. We proposed that the formation of *anti-*/*syn*-**1** or *anti-*/*syn*-**2** could be envisioned by the mechanism depicted in Scheme 4. Insertion of one molecule of the alkene into the Pd–C bond of the starting palladacycle (**a** or **b**) would afford the 10-membered alkyl palladacycle **I**. For this intermediate, we propose that the R′ group is at the carbon atom bonded to Pd(II), because (1) this is the regiochemistry that explains the formation of the allyl moiety coordinated to Pd(II) in complexes **1a**,**b** and **2a**,**b** and (2) this is the most frequent regioisomer found in the insertion of electron-poor alkenes into the Pd–C bonds of neutral complexes.^22,28^ Intermediate **I** could undergo β-hydride elimination to give the *cisoid*-(diene)PdH species **II**. *Syn* addition of Pd–H to the alkene moiety with regioselectivity in contrast with its previous elimination results in the formation of the η^3^-allyl complex *anti*-**1** or *anti*-**2** (Scheme 4), which seems to be formed preferentially as the kinetic product. On the other hand, the *transoid*-(diene)PdH intermediate **III** could be generated from **II** upon decoordination and rotation of the tertiary olefin moiety, prior to the Pd–H addition step, hence giving rise to the formation of the *syn* isomer. The thermal isomerization of the complexes *anti-***1** and *anti*-**2** to the *syn* isomers at 60 °C (Table 1) proceeded by the usual η^3^–η^1^–η^3^ rearrangement.

Complexes **1** and **2** were fully characterized by elemental analyses and NMR and IR spectroscopic techniques. The ^1^H NMR spectra of all the allyl complexes showed the diastereotopic nature of the hydrogen atoms of the NH_2_ and CH_2_ groups, as well as both of Me groups of the CMe_2_ moiety for **1b** and **2b**. The most characteristic signal was that corresponding to the methine group of the inserted fragment, which appeared as a doublet of doublets in the range 4.85–5.16 ppm for the *anti* isomers and 4.75–4.84 ppm for the *syn* isomers. The two coupling constants ^3^*J*_HH_ (*anti*, 11.2 ≤ ^3^*J*_HH_ ≤ 12.3 Hz, average value 11.7 Hz; *syn*, 9.6 ≤ ^3^*J*_HH_ ≤ 11.0 Hz, average value 10.3 Hz; *anti*, 4.5 ≤ ^3^*J*_HH_ ≤ 5.2 Hz, average value 4.7 Hz; *syn*, 3.7 ≤ ^3^*J*_HH_ ≤ 4.2 Hz, average value 4.0 Hz) were slightly smaller for the *syn* isomers than for the *anti* isomers.

**Figure 2:**
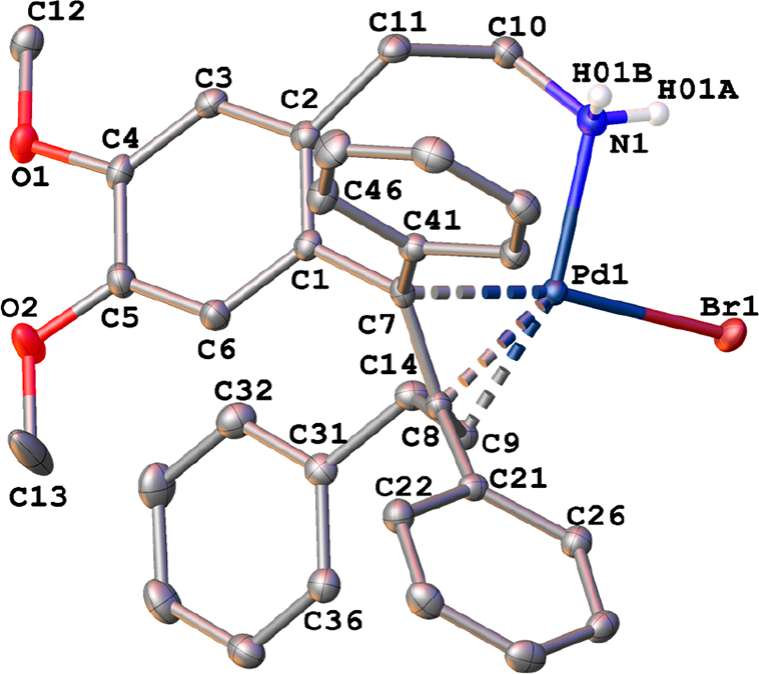
X-ray thermal ellipsoid plot (50% probability) of *anti-***1a** along with the labeling scheme. The hydrogen atoms bonded to carbon have been omitted for clarity. Selected bond lengths (Å) and angles (deg): Pd(1)–Br(1) = 2.4959(3), Pd(1)–N(1) = 2.1124(15), Pd(1)–C(7) = 2.1295(15), Pd(1)–C(8) = 2.1047(15), Pd(1)–C(9) = 2.1181(16), C(7)–C(8) = 1.458(2), C(8)–C(9) = 1.427(2); Br(1)–Pd(1)–N(1) = 92.82(4), C(7)–C(8)–C(9) = 119.41(14), C(8)–C(9)–C(14) = 131.14(14).

**Figure 3:**
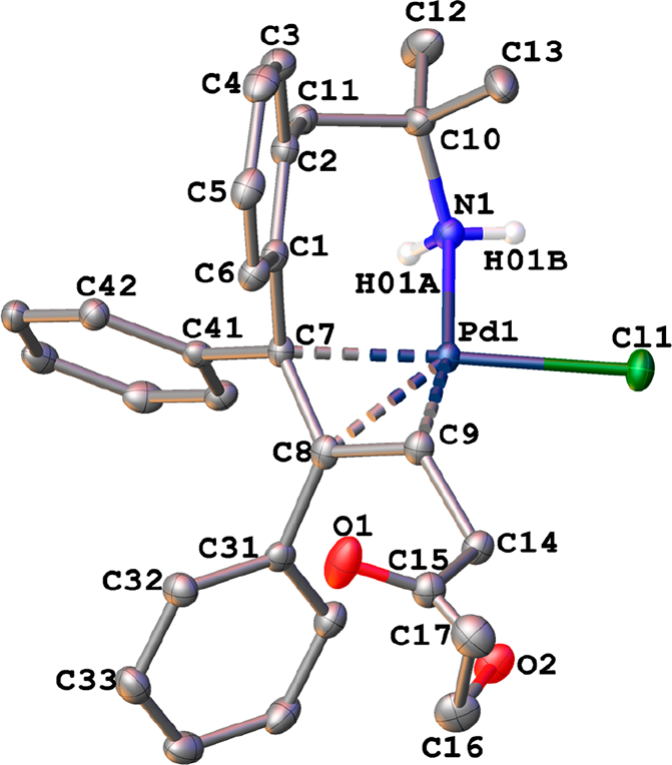
X-ray thermal ellipsoid plot (50% probability) of *syn-***2b** along with the labeling scheme. The hydrogen atoms bonded to carbon have been omitted for clarity. Selected bond lengths (Å) and angles (deg): Pd(1)–Cl(1) = 2.3864(5), Pd(1)–N(1) = 2.1257(18), Pd(1)–C(7) = 2.1198(19), Pd(1)–C(8) = 2.1400(19), Pd(1)–C(9) = 2.1167(19), C(7)–C(8) = 1.441(3), C(8)–C(9) = 1.421(3); Cl(1)–Pd(1)–N(1) = 89.81(5), C(7)–C(8)–C(9) = 117.38(18), C(8)–C(9)–C(14) = 122.49(18).

The crystal structures of the η^3^-allyl complexes *anti*-**1a**, *anti*-**1b**, *syn*-**1b**, *anti*-**2a**·CH_2_Cl_2_, *anti*-**2b**·CHCl_3_, and *syn*-**2b** were determined by X-ray diffraction (see the Supporting Information). The X-ray thermal ellipsoid plots of *anti*-**1a** and *syn*-**2b** are depicted in Figures 2 and 3, respectively. In both cases, the palladium atoms adopted a square-planar geometry (mean deviation from the plane Y–Pd(1)–X(1)–N(1) 0.0194 Å, where Y = centroid from C(7), C(8), and C(9) atoms). The halogeno ligand and the amino group of the metalated fragment were coordinated in *cis* positions. The other two coordination sites were occupied by the allyl moiety, which was η^3^-bonded via C(7), C(8), and C(9), with C(7)–C(8)–C(9) angles of 119.4 and 122.5° for *anti-***1a** and *syn-***2b**, respectively.

Some catalytic transformations involving the sequential migratory insertion of alkynes and alkenes into a Pd–C^11,12,17,29^ or a Pd–H^15^ bond have been reported in the literature. In these processes, substituted 1,3-butadienes are formed. The mechanism proposed for those catalytic transformations does not consider the formation of η^3^-allyl intermediates. In our case, however, the presence of a coordinating group, such as NH_2_, may favor the isomerization of the 1,3-butadiene to form a stabilized allyl Pd(II) complex. As far as we are aware, this is the first stoichiometric study where the sequential migratory insertion of an alkyne and an alkene has been studied.

#### Reactivity of the η^3^-Allyl Complexes

**Scheme 5:**
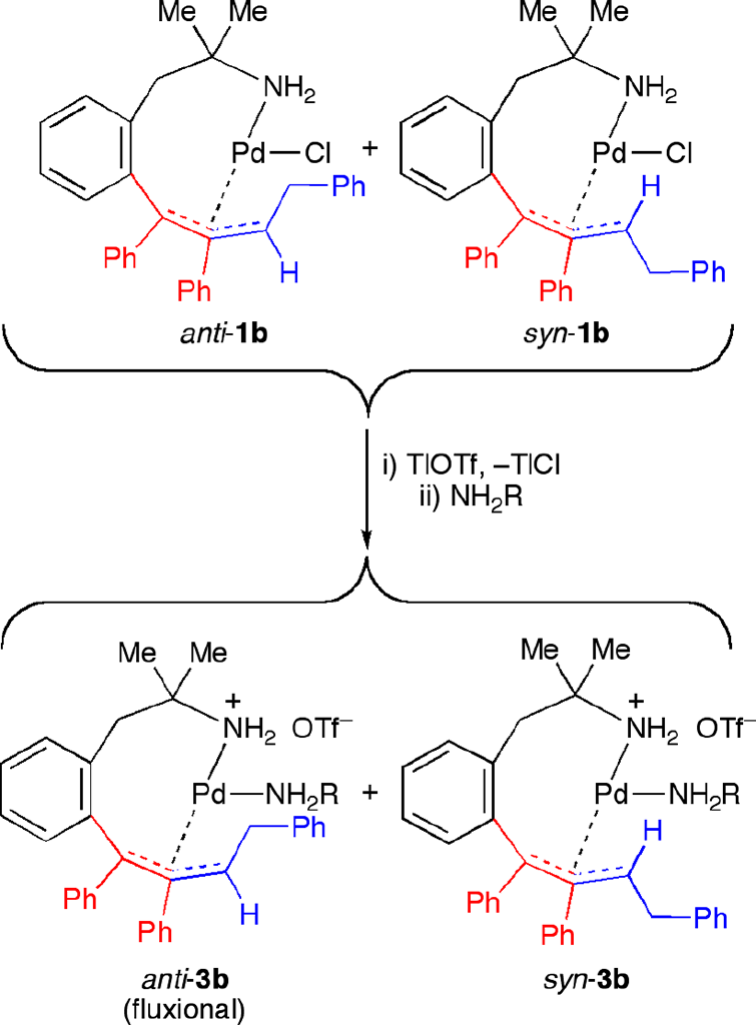
Reactions of η^3^-Allyl Complex **1b** with *p*-Toluidine

The nucleophilic attack to η^3^-allyl Pd(II) intermediates to give functionalized alkenes is a well-known strategy in organic synthesis.^30^ However, most of the times the key η^3^-allyl Pd(II) species are generated through the oxidative addition of allyl halides, pseudohalides or acetates to Pd(0). We wondered if the η^3^-allyl complexes **1** and **2** could undergo the attack of a suitable nucleophile to render an organic derivative. Hence, we performed a range of reactions with several nucleophiles, such as KCN, [Tl(acac)], *p*-toluidine, and the phosphorus ylide Ph_3_PCHCOMe. There was no reaction at room temperature with any of these nucleophiles. When the reaction mixture was heated in refluxing toluene, complicated mixtures were produced. The reaction of complex **1b** and dimethyl malonate in the presence of Cs_2_CO_3_ (CHCl_3_, room temperature) afforded an *anti*/*syn* mixture of isomers of a new complex containing the η^3^-allyl moiety and a coordinated malonate, whose structures were not studied further. When a solution of this complex in CHCl_3_ was heated at 60 °C, again a complicated mixture was obtained. In order to facilitate the nucleophilic attack, we performed the reaction of the η^3^-allyl complex **1b** with *p*-toluidine in the presence of TlOTf, which would generate a cationic complex by replacing the Cl^–^ ligand by OTf^–^ in the coordination sphere of Pd(II). In this case, the stable cationic η^3^-allyl complex **3b** was isolated, which contained a coordinated *p*-toluidine ligand (Scheme 5). This complex was stable in refluxing toluene and did not decompose to the expected organic product.

**Figure 4:**
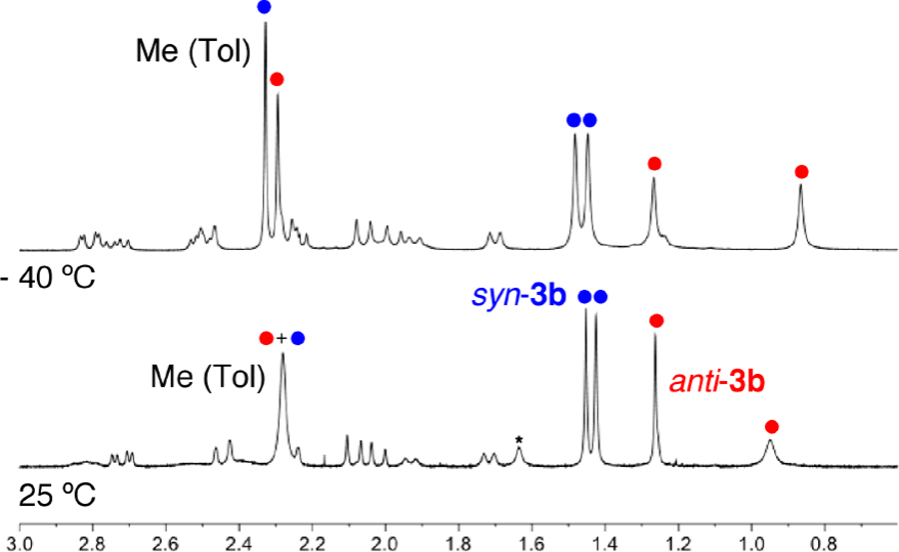
^1^H NMR spectra (0.6–3.0 ppm) of complex **3b** (400 MHz) in CDCl_3_ at 25 °C (bottom) and −40 °C (top). The asterisk indicates the signal corresponding to H_2_O. The blue and red circles correspond to the *syn* and *anti* isomers, respectively.

The ^1^H NMR of complex **3b** at room temperature showed some broad signals and seemed to correspond to a mixture of two isomers with a fluxional behavior (Figure 4). Perhaps the most significant resonances are those attributed to the Me groups: three sharp singlets at 1.45 (3 H), 1.42 (3 H), and 1.26 ppm (2.2 H) and two broad singlets at 2.28 (5.4 H) and 0.94 ppm (2.2 H). When the spectrum was measured at −40 °C, the broad singlet below 1 ppm become sharper, and the signal at 2.28 ppm split into two new singlets with the relative intensities 3:2.2. According to these data, we assumed that both isomers, *anti-*/*syn*-**3b**, are formed in a 1.33/1 ratio, one of which presented a fluxional behavior in solution. The less stable isomer should be *anti*-**3b** due to steric hindrance of the toluidine ligand and the substituent on the terminal allylic carbon, and for it, it was reasonable to suppose that the neutral ligand could be coming in and out of the metal coordination sphere.

**Figure 5:**
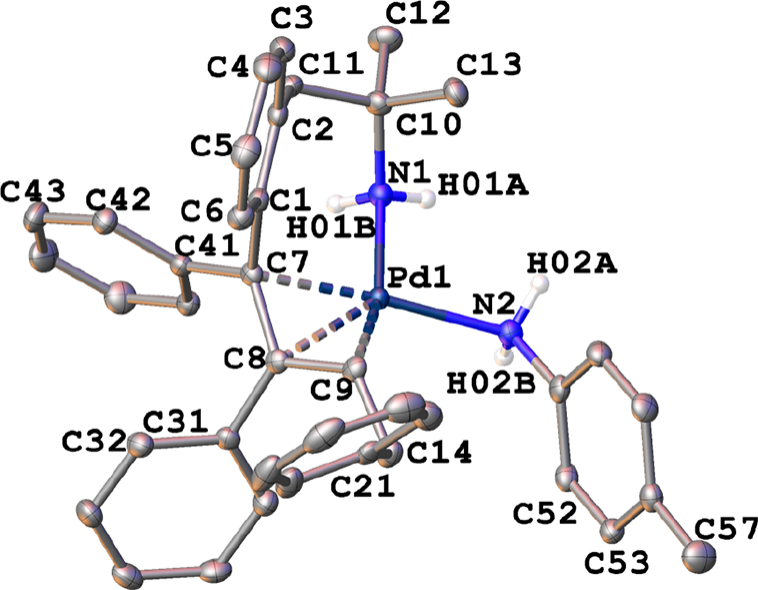
Thermal ellipsoid plot (50% probability) of the cation of one (A) of the two independent molecules of the complex *syn*-**3b·**CH_2_Cl_2_ showing the labeling scheme. The solvent molecule and the hydrogen atoms bonded to carbon have been omitted for clarity. Selected bond lengths (Å) and angles (deg) are given for both independent molecules (A and A′). For A: Pd(1)–N(1) = 2.131(2), Pd(1)–N(2) = 2.161(2), Pd(1)–C(7) = 2.100(2), Pd(1)–C(8) = 2.138(2), Pd(1)–C(9) = 2.165(2), C(7)–C(8) = 1.454(3), C(8)–C(9) = 1.408(3); N(1)–Pd(1)–N(2) = 90.26(9), C(7)–C(8)–C(9) = 116.9(2), C(8)–C(9)–C(14) = 124.9(2). For A′: Pd(1′)–N(1′) = 2.130(2), Pd(1′)–N(2′) = 2.163(2), Pd(1′)–C(7′) = 2.117(2), Pd(1′)–C(8′) = 2.134(2), Pd(1′)–C(9′) = 2.165(2), C(7′)–C(8′) = 1.452(3), C(8′)–C(9′) = 1.413(3); N(1′)–Pd(1′)–N(2′) = 88.94(8), C(7′)–C(8′)–C(9′) = 117.5(2), C(8)–C(9)–C(14) = 124.0(2).

The crystal structure of the η^3^-allyl complex *syn*-**3b**·CH_2_Cl_2_ was determined by X-ray diffraction (Figure 5), showing that the phenyl and benzyl substituents at the meso and terminal carbon atoms of the allylic unit occupied mutually *syn* positions and indicating that this was, in fact, the most stable isomer. For this complex there were two independent molecules in the asymmetric unit (A and A′). For molecule A, the palladium atom exhibited a square-planar geometry (mean deviation from the plane: X–Pd(1)–N(1)–N(2) 0.0067 Å, where X = centroid from C(7), C(8), and C(9) atoms). Both amino groups were coordinated in *cis* positions. The other two coordination sites were occupied by the allyl moiety, which was η^3^-bonded via C(7), C(8), and C(9), with a C(7)–C(8)–C(9) angle of 116.9(2)°. The allyl plane, defined by atoms C(7), C(8), and C(9), formed a dihedral angle of 118.1° with the Pd(II) coordination plane. The aromatic rings formed angles of 71.3° (metalated ring), 65.5° (phenyl ring at C7), 71.9° (phenyl ring at C8), 73.3° (phenyl ring at C14), and 92.4° (toluidine ring) with respect to the Pd coordination plane, to avoid steric hindrance. Hydrogen bond interactions were observed between the cationic palladium moiety and the OTf^–^ anion (see the Supporting Information).

**Scheme 6:**
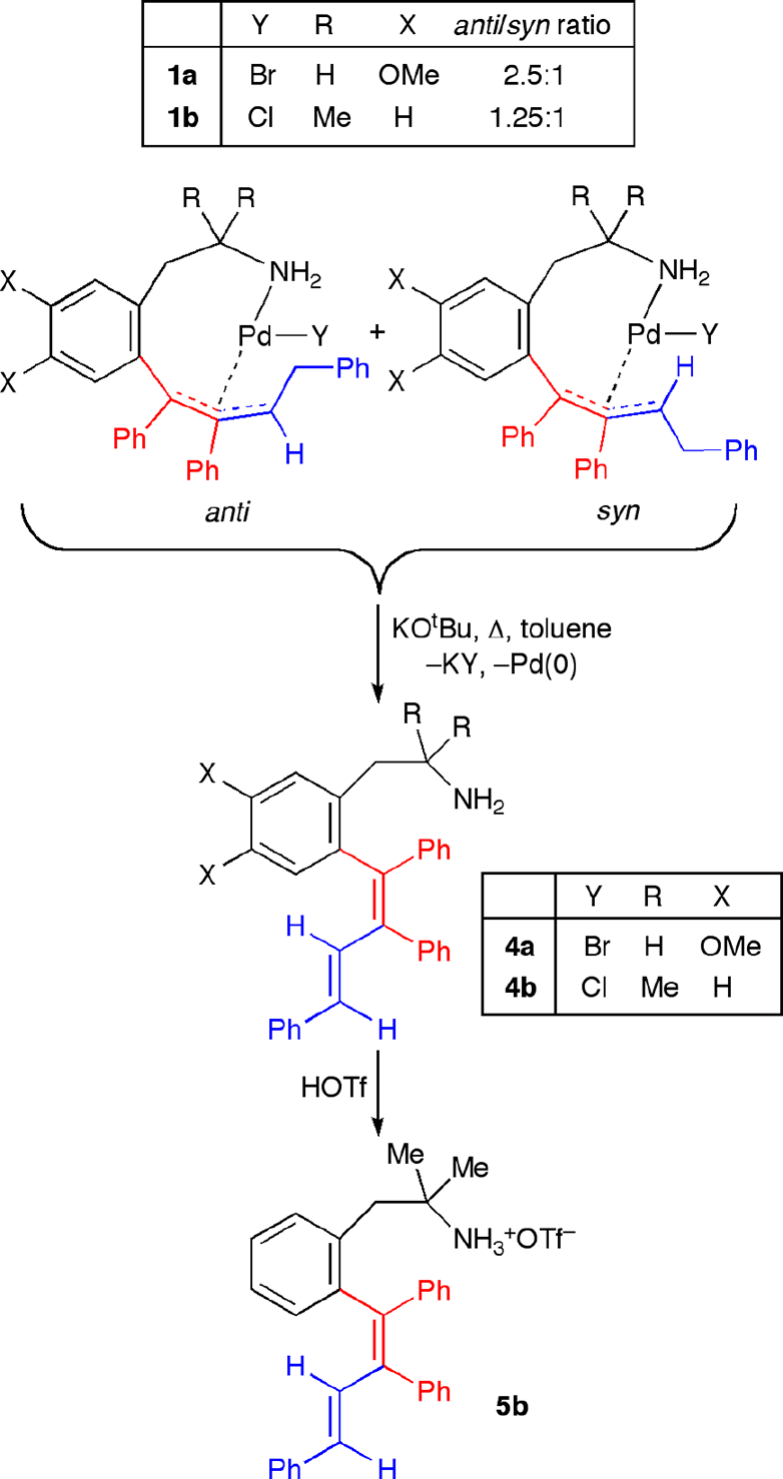
Synthesis of Functionalized Phenethylamine Derivatives Containing 1,3-Butadienyl Substituents

Additionally, we studied the behavior of the *anti-*/*syn*-**1a**,**b** complexes toward a strong base. The reaction of these complexes with KO^t^Bu in toluene under a N_2_ atmosphere afforded Pd(0) and the functionalized phenethylamines containing a 1,3-butadienyl substituent in an *ortho* position (**4a**,**b**; Scheme 6). We did not observe intramolecular cyclization arising from the possible nucleophilic attack of the NH_2_ group to the η^3^-allyl moiety. The compound **4a** was a colorless solid and was easily isolated, but **4b** was obtained as a liquid. In order to get a more easily isolable derivative, triflic acid was added to a solution of compound **4b** in diethyl ether (Scheme 6). The corresponding ammonium triflate **5b** precipitated in the reaction mixture and was obtained as a white powder in moderate yield (50%).

Miura et al.^12^ described the intermolecular three-component coupling of aryl iodides, diarylacetylenes, and alkenes in the presence of palladium acetylacetonate and silver acetate as catalysts, to give the corresponding 1:1:1 and 1:2:1 coupling products (1,3-butadiene and 1,3,5-hexatriene derivatives, respectively). The mechanism proposed for these catalytic transformations followed the sequential steps analogous to those of the synthesis of compounds **4** reported here, (1) formation of an aryl Pd(II) complex (either by oxidative addition or C–H activation), (2) alkyne insertion into the Pd–C bond to form an alkenyl complex, (3) alkene insertion into the Pd–C bond to form an alkyl intermediate, and (4) β-H elimination, assisted by a base, to afford the final diene. In our case, and due to the particular nature of the initial aryl group, we have been able to isolate all of the organometallic intermediates involved in the process.

**Figure 6:**
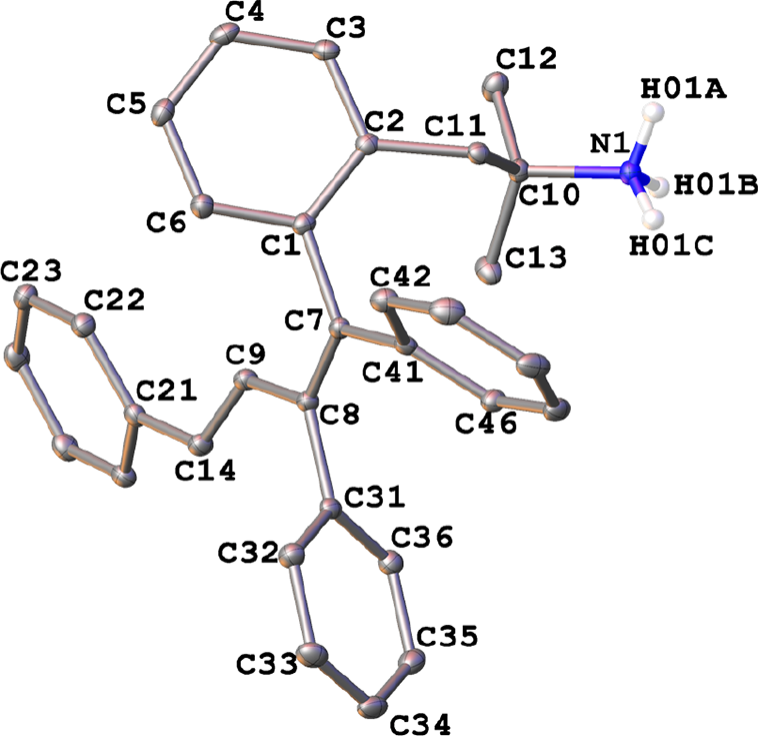
Thermal ellipsoid plot (50% probability) of the cation of compound **5b**·H_2_O along with the labeling scheme. The solvent molecule and the hydrogen atoms bonded to carbon have been omitted for clarity. Selected bond lengths (Å) and angles (deg): C(1)–C(7) = 1.503(2), C(7)–C(8) = 1.359(2), C(8)–C(9) = 1.461(2), C(9)–C(14) = 1.341(2), C(14)–C(21) = 1.466(2), C(8)–C(31) = 1.496(2), C(7)–C(41) = 1.487(2); C(1)–C(7)–C(41) = 115.93(15), C(1)–C(7)–C(8) = 120.51(16), C(7)–C(8)–C(9) = 120.85(16), C(8)–C(9)–C(14) = 127.20(17), C(9)–C(8)–C(31) = 116.96(15).

The crystal structure of the ammonium triflate **5b**·H_2_O was solved by X-ray diffraction studies (Figure 6) and showed a slightly distorted planar diene skeleton (mean deviation from the plane: C(7)–C(8)–C(9)–C(1)–C(14)–C(21)–C(31)–C(41) = 0.041 Å) with an *E*,*E* geometry. Within the butadiene fragment, the average C–C double-bond distance (C7–C8, C9–C14) was 1.35(2) Å, being slightly longer than that corresponding to the most substituted unsaturated unit, and the average single-bond value (C1–C7, C7–C41, C8–C9, C8–C31, C14–C21) was 1.48(2) Å. The aryl rings formed angles of 62.5, 20.8, 69.1, and 48.0° with respect to the diene plane, adopting a helical conformation and releasing steric hindrance.

We also attempted to insert a third unsaturated molecule into the Pd–C bonds of the η^3^-allyl complexes by bubbling CO through a solution of **1b** in CH_2_Cl_2_ (room temperature, 4 h). Nevertheless, no reaction was observed, probably because these complexes were too stable. A similar reaction, with addition of TlOTf and use of THF as the solvent, led to a cationic complex with no CO inserted.

### Insertion of 2-Norbornene into the Pd–C Bond

#### Synthesis and Structure of 10-Membered Norbornyl Palladium(II) Complexes

**Scheme 7:**
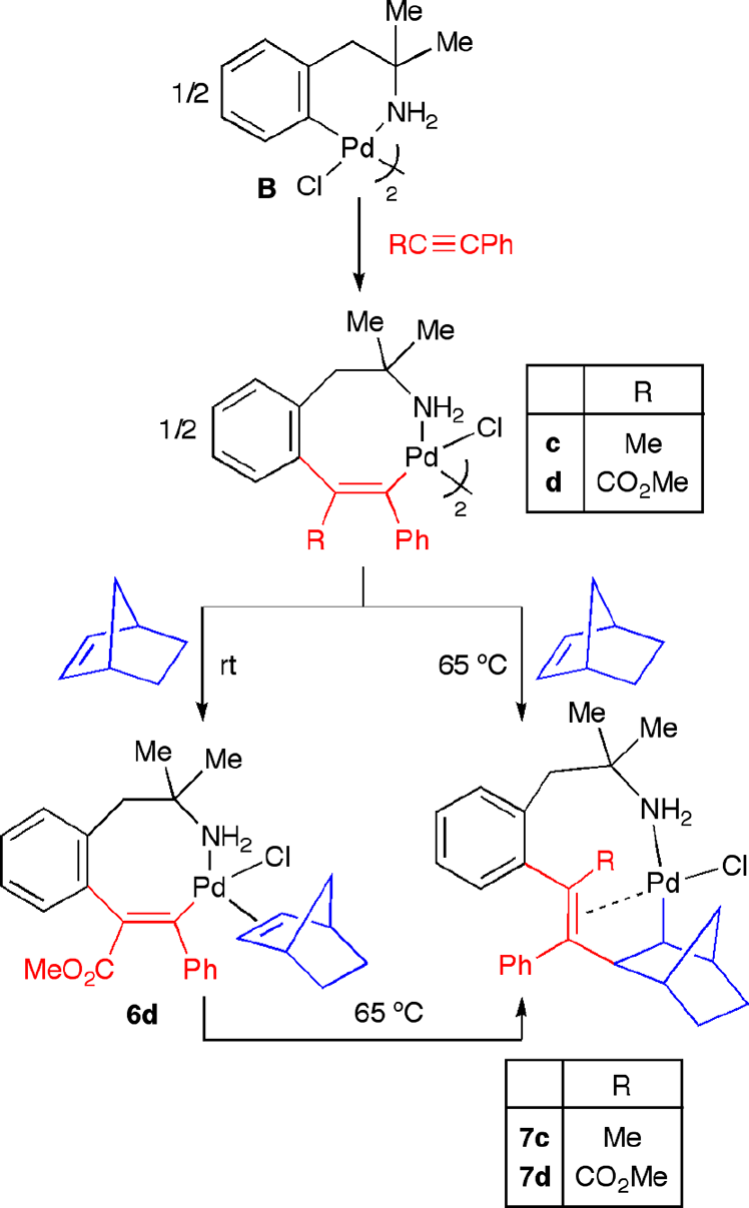
Sequential Insertion of Alkyne/2-Norbornene into the Pd–C Bond of Six-Membered Palladacycle **B**

In contrast to styrene or ethyl acrylate, when 1 equiv of 2-norbornene was added to a solution of the alkenyl complex **b** (arising from insertion of one molecule of diphenylacetylene into the Pd–C bond of palladacycle **B**; see Scheme 7 for its structural formula), no insertion reaction was observed, neither at room temperature nor on heating the mixture to 65 °C in CHCl_3_. We also attempted the insertion reactions using as starting materials the eight-membered palladacycles **c** and **d**, arising from the insertion of methyl phenylpropiolate and 1-phenyl-1-propyne, respectively, into the Pd–C bond of complex **B**. When palladacycle **d** was used, the reaction at room temperature led to a new species that was tentatively assigned to the norbornene coordination monomer species **6d** (Scheme 7), which could not be fully characterized, since it evolved easily to the starting dimeric complex **d** when we tried to crystallize it. Nevertheless, when these reaction mixtures (palladacycle **c** or **d** and 2-norbornene) were heated to 65 °C, we could successfully isolate the complexes **7c**,**d**, where the insertion of the 2-norbornene moiety into the alkenyl Pd(II) complex had taken place (Scheme 7). These complexes have a monomeric nature, since the Pd center completes its coordination sphere with the intramolecular olefin moiety.

**Figure 7:**
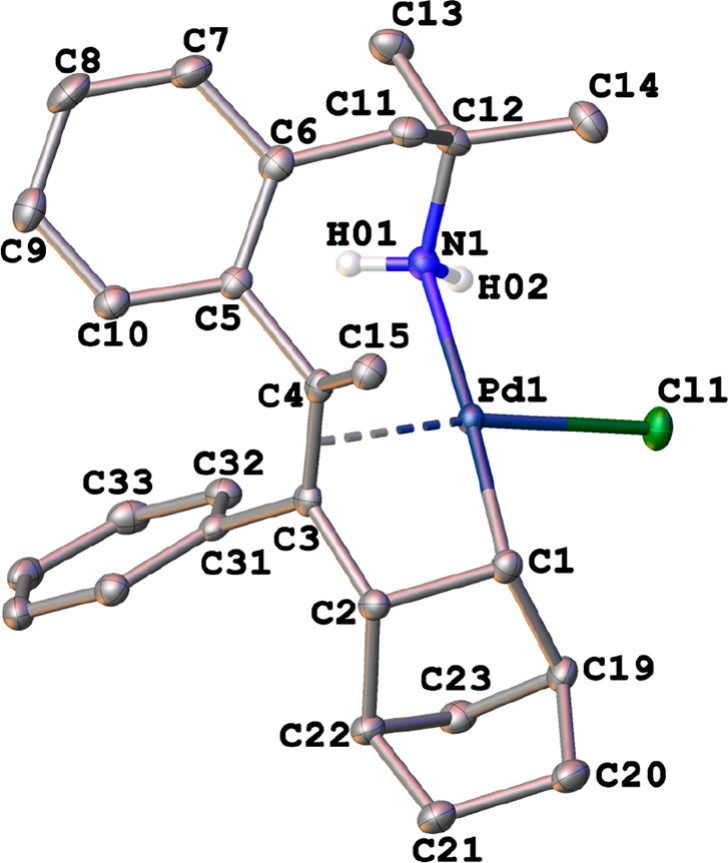
Thermal ellipsoid plot (50% probability) of the complex **7c**·CHCl_3_ along with the labeling scheme. The solvent molecule and the hydrogen atoms bonded to carbon have been omitted for clarity. Selected bond lengths (Å) and angles (deg): Pd(1)–N(1) = 2.2185(19), Pd(1)–Cl(1) = 2.3589(5), Pd(1)–C(1) = 2.047(2), Pd(1)–C(3) = 2.159(2), Pd(1)–C(4) = 2.203(2), Pd(1)–X = 2.065, C(1)–C(2) = 1.553(3), C(2)–C(3) = 1.542(3), C(3)–C(4) = 1.403(3), C(4)–C(5) = 1.512(3); N(1)–Pd(1)–Cl(1) = 88.03(5), Cl(1)–Pd(1)–C(1) = 93.88(6), C(1)–Pd(1)–X = 76.7, X–Pd(1)–N(1) = 101.3, C(2)–C(3)–C(4) = 117.17(17), C(3)–C(4)–C(5) = 122.65(18). X represents the midpoint of the double bond C(3)–C(4).

The crystal structures of complexes **7c**·CHCl_3_ and **7d**·1/2CHCl_3_ were solved by X-ray diffraction studies (see Figure 7 and the Supporting Information), and both showed similar features. For the complex **7c**·CHCl_3_, the atoms Cl(1), N(1), and C(1) together with the midpoint of the C(3)–C(4) double bond form a square plane around the palladium atom (mean deviation from the plane: Pd(1)–C(1)–N(1)–Cl(1)–X 0.0230 Å, where X = centroid from C(3) and C(4) atoms). The norbornene unit adopted an *exo* conformation, arising from the *syn* addition of the Pd–C bond to the *exo* face of the olefin, as expected. The C(3)–C(4) double bond forms an angle of 61.8° with the palladium coordination plane. The methyl and phenyl substituents are mutually *trans*, which requires the isomerization of the first inserted alkyne. This arrangement reduces the steric hindrance between the substituents and is the normal behavior for di-inserted derivatives containing cyclometalated amines.^31^

**Scheme 8:**
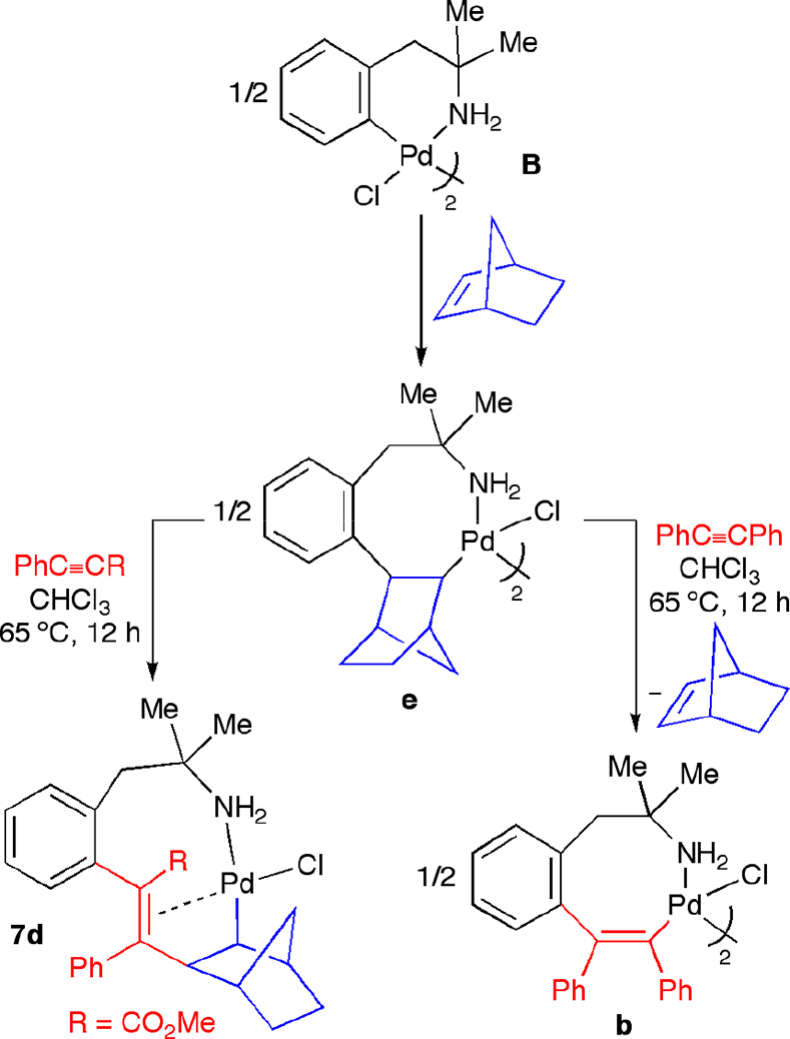
Sequential Insertion of Norbornene/Alkynes into the Pd–C Bond of Palladacycle **B**

We performed an analogous experiment reversing the order of the addition of the unsaturated reagents. That is, we studied the reactivity of the previously reported norbornyl derivative **e** (arising from the insertion of 2-norbornene into the six-membered palladacyle **B**) toward alkynes (Scheme 8). A suspension of the norbornyl Pd(II) complex **e** in CHCl_3_ was heated to 65 °C in the presence of methyl phenylpropiolate. The complex **7d**, which arose from a reverse insertion order of the reagent addition, was isolated in 20% yield from the reaction mixture. In this reaction, palladacycle **b** was also formed. The formation of **7d** could be explained through the deinsertion of the 2-norbornene fragment in **e** to give the starting palladacycle **B**, which in turn would undergo the sequential insertion of the alkyne and 2-norbornene. The ability of this cyclic olefin to insert reversibly into Pd–aryl bonds is well-known. This behavior has promoted the use of norbornene as an essential ligand in the functionalization of haloarenes: for instance, in the versatile Catellani type reaction.^2^ We tried to apply this strategy to obtain the diphenylacetylene/2-norbornene insertion derivative. Nevertheless, the reaction of the norbornyl complex **e** with diphenylacetylene gave rise mainly to the stable eight-membered palladacycle **b**, arising from monoinsertion of the alkyne (Scheme 8). That is, the olefin did not insert into the Pd–C bond of **b**, as noted above.

**Scheme 9:**
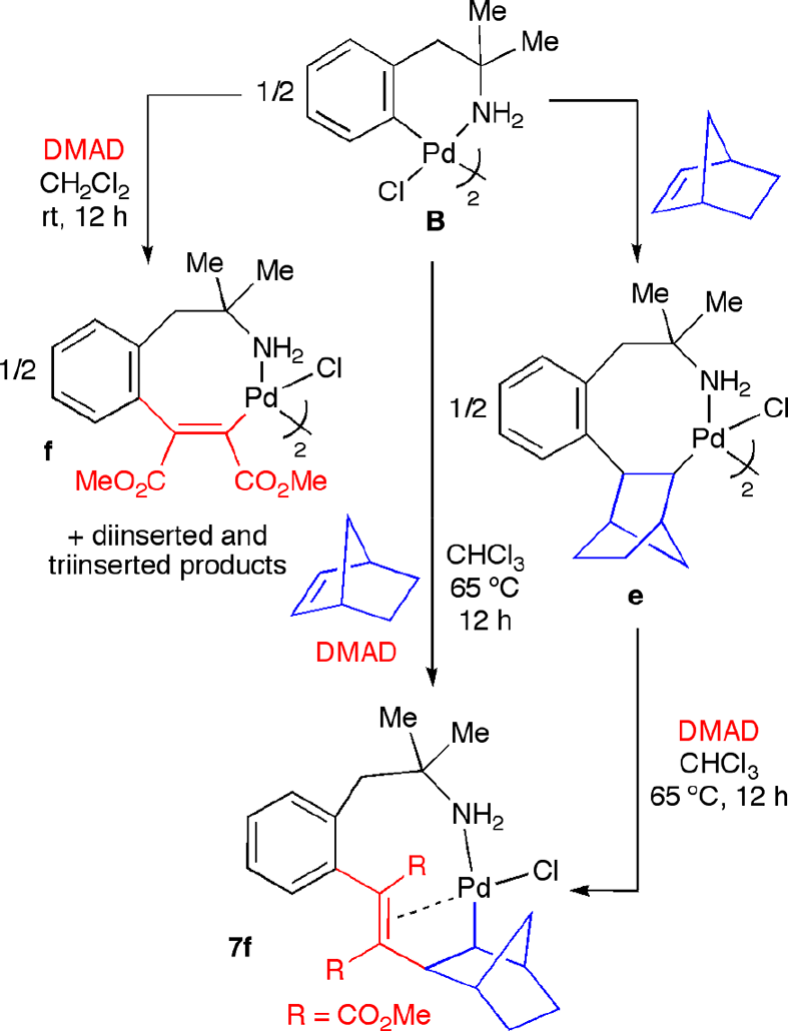
Sequential Insertion of Norbornene/DMAD into the Pd–C Bond of Palladacycle **B**

The synthesis of alkenyl palladacycles arising from the insertion of one molecule of alkyne into the Pd–C bond of a starting complex is not always a simple task, due to the possibility of di- and tri-insertion processes. This aspect is especially relevant when a very electrophilic alkyne, such as dimethyl acetylenedicarboxylate (DMAD), is used. We have previously tried to isolate the palladacycle **f** arising from monoinsertion of DMAD into the Pd–C bond of **B**; nevertheless, mixtures of mono-, di-, and tri-inserted products were obtained (Scheme 9). We thought that performing the reaction of **B** and DMAD in the presence of 2-norbornene could drive the reaction to the formation of a sequential DMAD/norbornene insertion product, given the fact that norbornene seems to react readily with other monoinserted alkyne derivatives. Indeed, when a solution of the palladacycle **B** in CHCl_3_ was heated to 65 °C in the presence of 1 equiv of DMAD and 1 equiv of 2-norbornene, the alkyne/alkene sequential insertion product **7f** was isolated in good yield (Scheme 9). The crystal structure of complex **7f** was also solved by X-ray diffraction studies (see the Supporting Information) and showed features similar to those discussed for **7c**·CHCl_3_.

#### Reactivity of the Norbornyl Complexes

**Scheme 10:**
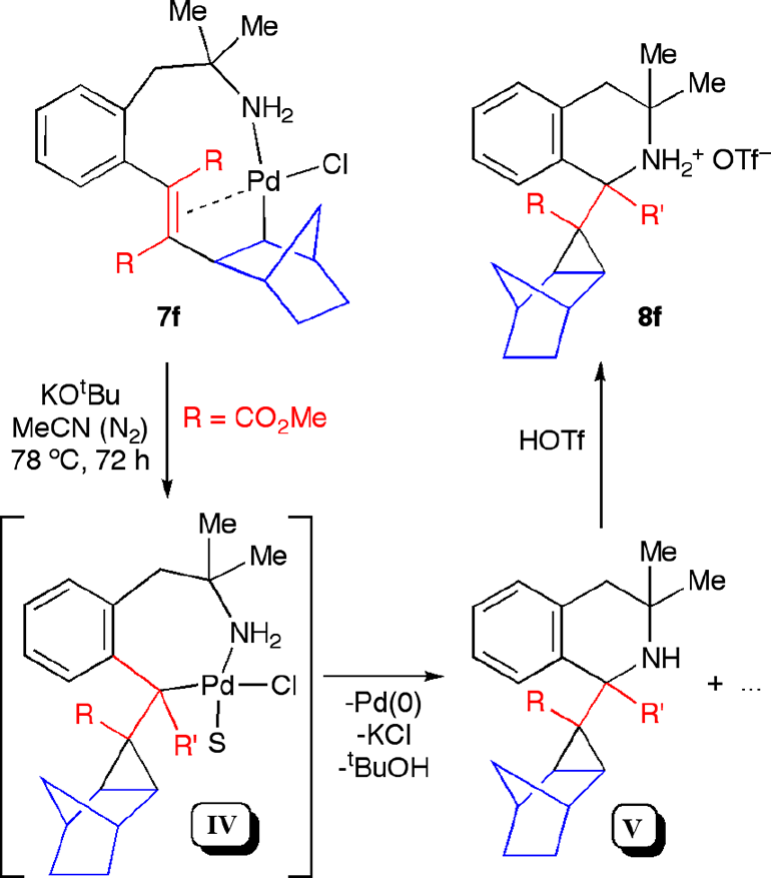
Reaction of Complex **7f** with KO^t^Bu

We explored the reactivity of the complex **7f** toward KO^t^Bu in MeCN at 78 °C. From the reaction mixture, we could isolate the tetrahydroisoquinoline derivative **8f** by treatment of the crude product with HOTf: that is, from the protonation of **V** (Scheme 10). A possible path to explain the formation of **8f** would involve the intramolecular carbopalladation of the alkene moiety, giving rise to a cyclopropyl ring. The new organometallic intermediate **IV** would then undergo a C–N coupling process with reductive elimination of Pd(0). Several Pd-catalyzed procedures to promote the cyclopropanation of 2-norbornene have been reported in the literature.^32^ Some of these procedures rely on the migratory insertion of norbornene into a Pd alkynyl^33^ or Pd alkenyl^34^ complex, and some organometallic intermediates similar to complexes **7** have been proposed, where the alkenyl moiety is coordinated intramolecularly to Pd.

**Figure 8:**
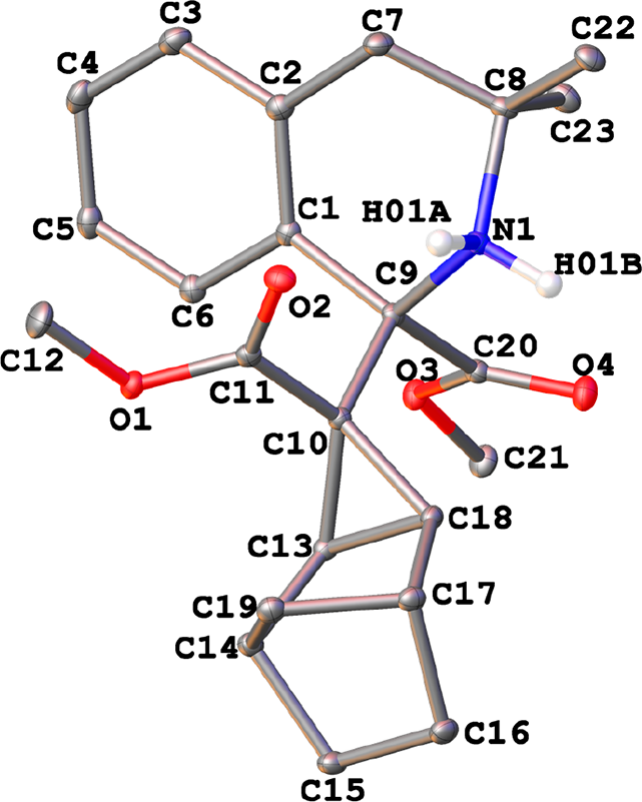
Thermal ellipsoid plot (50% probability) of the cation of compound **8f** along with the labeling scheme. The hydrogen atoms bonded to carbon have been omitted for clarity. Selected bond lengths (Å) and angles (deg): N(1)–C(8) = 1.5233(17), N(1)–C(9) = 1.5134(16), C(9)–C(10) = 1.5595(18), C(10)–C(13) = 1.5071(17), C(10)–C(18) = 1.5259(18), C(13)–C(18) = 1.5261(17); C(1)–C(9)–N(1) = 111.04(10), N(1)–C(9)–C(20) = 105.93(10), C(20)–C(9)–C(10) = 110.53(10), C(10)–C(18)–C(13) = 59.18(8), C(18)–C(13)–C(10) = 60.40(8), C(13)–C(10)–C(18) = 60.42(8).

The crystal structure of **8f** was solved by X-ray diffraction studies (Figure 8) and showed the isoquinoline nucleus derived from phentermine substituted at C1 with a methoxycarbonyl group and a tricyclo[3.2.1]octyl ring. It is worth noting that highly strained hydrocarbons such as this cyclopropane-containing norbornyl moiety have been tested as new highly energetic materials for liquid-fueled propulsion systems.^35^

**Scheme 11:**
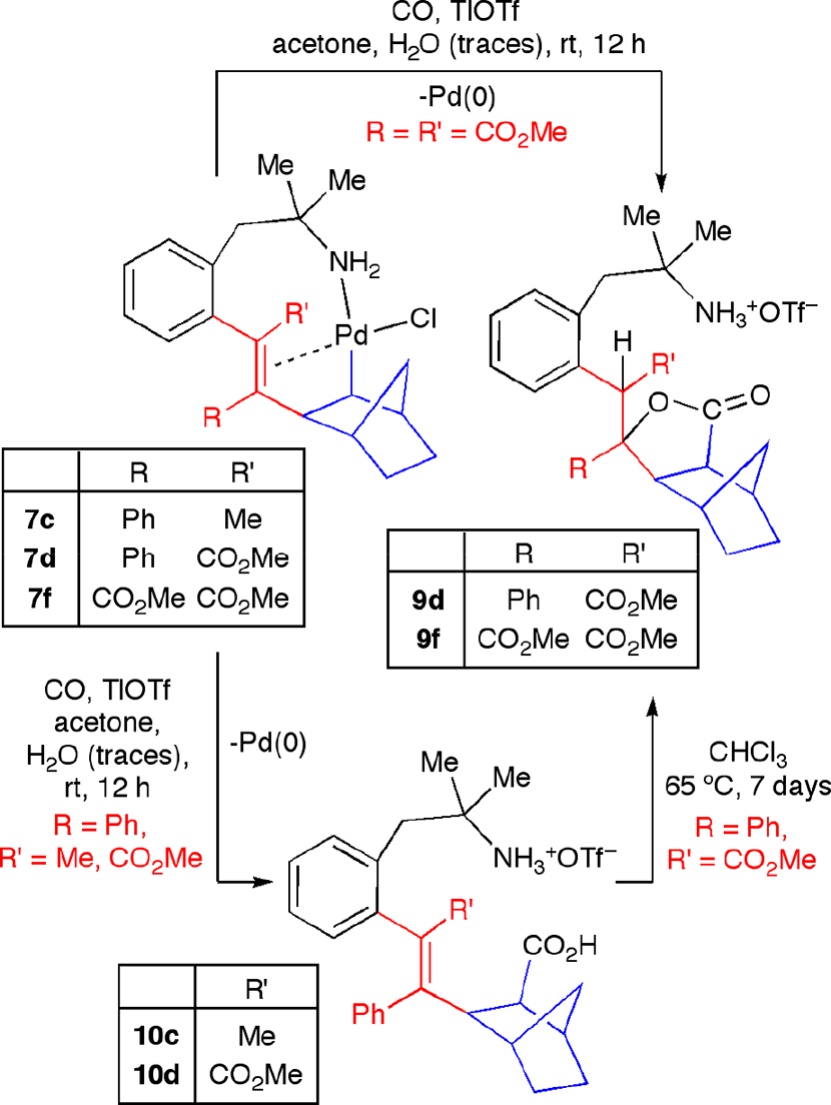
Reactions of Complexes **7** with CO

We studied whether a further insertion of an unsaturated molecule could be carried out in the complexes **7c**,**d**,**f**, obtained after alkyne/norbornene insertion. Complex **7f** did not react when it was stirred in CHCl_3_ in the presence of CO at room temperature for 5 h. When the reaction mixture was heated to 65 °C, a complicated mixture was formed. In order to facilitate the insertion of CO, we performed the reaction in the presence of TlOTf in acetone, hence generating a cationic Pd(II) intermediate *in situ*. In this case, the reaction afforded the lactone **9f** and Pd(0) (Scheme 11). The formation of **9f** could be explained as the result of the hydrolysis of the acyl Pd(II) intermediate generated upon CO insertion into the Pd–C bond present in **7f**. Under those conditions, the carboxylic acid derivative underwent an intramolecular Michael addition of the CO_2_H group to the α,β-unsaturated ester moiety.

When the cationic derivatives of complexes **7c**,**d** (generated *in situ*) reacted with CO at room temperature, the amino acid derivatives **10c**,**d** were obtained (Scheme 11). In the case of **10d**, intramolecular cyclization to give **9d** took place after heating at 65 °C in CHCl_3_ for 7 days. As expected, the derivative **10c** was stable upon heating, since it was not activated for the Michael addition step, as occurred in the cases of the alkenyl complexes bearing a CO_2_Me substituent.

**Figure 9:**
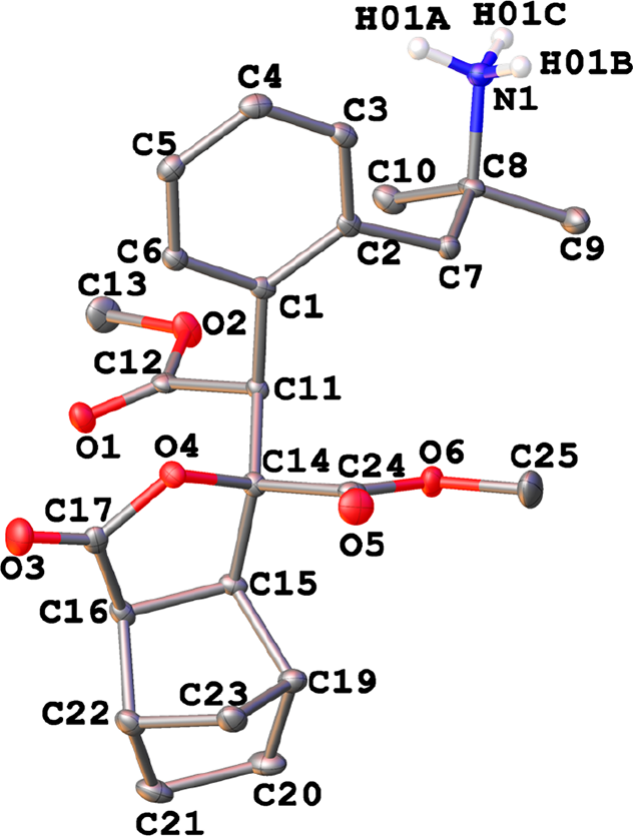
Thermal ellipsoid plot (50% probability) of the cation of **9f**·Et_2_O along with the labeling scheme. The solvent molecule and the hydrogen atoms bonded to carbon have been omitted for clarity. Selected bond lengths (Å) and angles (deg): N(1)–C(8) = 1.5209(19), C(11)–C(12) = 1.522(2), C(11)–C(14) = 1.557(2), C(14)–O(4) = 1.4423(18), C(17)–O(3) = 1.2010(19), C(17)–O(4) = 1.3514(19), C(16)–C(17) = 1.499(2); C(1)–C(11)–C(12) = 107.19(12), C(11)–C(14)–O(4) = 109.88(12), C(14)–O(4)–C(17) = 111.86(11), O(4)–C(17)–C(16) = 111.77(13), C(17)–C(16)–C(15) = 104.53(12), C(16)–C(15)–C(14) = 104.02(12), C(15)–C(14)–O(4) = 106.07(11).

The crystal structure of **9f**·Et_2_O has been solved by X-ray diffraction studies (Figure 9) and showed an ammonium salt derived from the starting phentermine containing a bicyclo[2.2.1]heptane lactone substituent. The hexahydro-4,7-methanoisobenzofuran-1(3*H*)-one core is a known compound that has attracted interest because of its potential use as a prostaglandin/thromboxane receptor antagonist.^36^ The most frequent routes for its synthesis involved (a) the cycloaddition reaction of furan-2(5*H*)-one and cyclopentadiene^37^ and (b) the oxidation of *cis*-2,3-bis(hydroxymethyl)bicyclo[2.2.1]heptane, which could be performed in a stoichiometric way with standard organic oxidants^38^ or by catalysis, with the use of enzymes^39^ or transition metals.^40^

**Figure 10:**
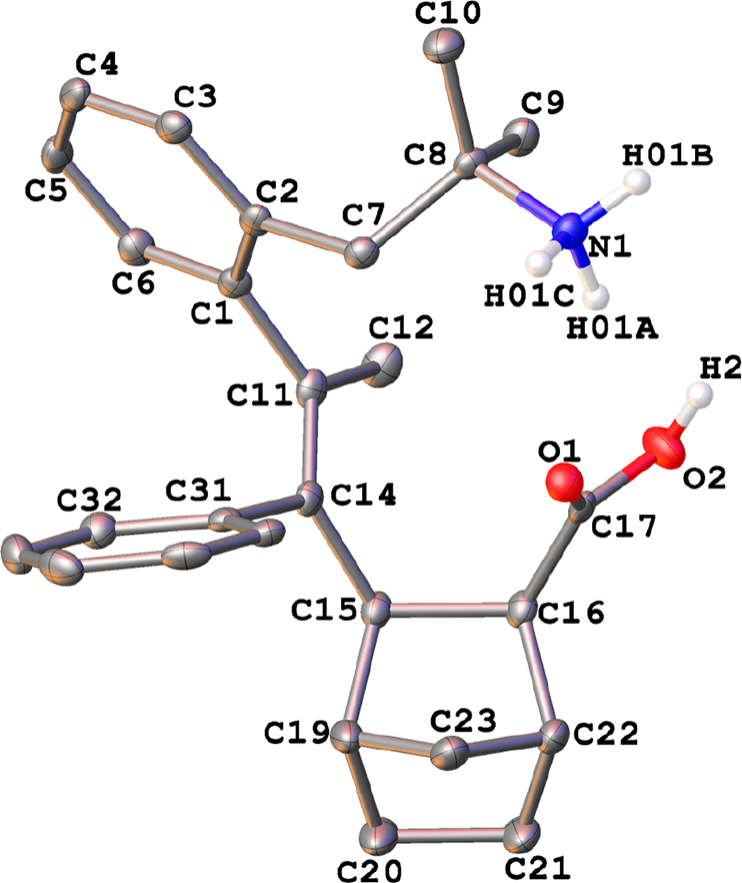
Thermal ellipsoid plot (50% probability) of the cation of **10c**·H_2_O along with the labeling scheme. The solvent molecule and the hydrogen atoms bonded to carbon have been omitted for clarity. Selected bond lengths (Å) and angles (deg): N(1)–C(8) = 1.518(2), C(1)–C(11) = 1.504(3), C(11)–C(14) = 1.342(3), C(14)–C(15) = 1.532(2), C(15)–C(16) = 1.582(2), C(16)–C(17) = 1.506(3), C(17)–O(1) = 1.218(2), C(17)–O(2) = 1.328(2); N(1)–C(8)–C(7) = 104.46(14), C(1)–(11)–C(14) = 121.73(17), C(11)–C(14)–C(15) = 121.56(17), C(14)–C(15)–C(16) = 118.26(15), C(15)–C(16)–C(17) = 116.58(15), C(16)–C(17)–O(1) = 126.22(17), C(16)–C(17)–O(2) = 111.65(16).

The crystal structure of **10c** has also been determined by X-ray diffraction studies (Figure 10), and it showed the ammonium salt derived from the sequential insertion of alkyne/norbornene/CO into the initial phentermine palladacycle. Both substituents (the carboxylate and the alkenyl groups) at the norbornadienyl moiety were in an *exo* disposition.

## Conclusion

In summary, the stoichiometric sequential insertion of alkynes and alkenes into the Pd–C bond of metalated phenethylamines has been studied. The result of these reactions depends on the nature of the olefin used. The insertion of styrene or ethyl acrylate into the eight-membered palladacycle arising from monoinsertion of diphenylacetylene affords the η^3^-allyl species **1** or **2**, via sequential β-H elimination/hydropalladation steps. However, the insertion of 2-norbornene leads to norbornyl Pd(II) complexes with an alkenyl moiety intramolecularly coordinated to the metal center.

The treatment with a strong base of the organometallic complexes obtained upon the sequential insertion of alkyne/alkene (**1**, **2**, and **7**) also led to different results. While the η^3^-allyl complexes **1** and **2** afforded 1,3-butadienyl-substituted phenethylamines (**4b** and **5b**), the norbornyl Pd derivative **7f** gave a tetrahydroisoquinoline derivative upon cyclopropanation of the norbornene fragment and C–N coupling (**8f**).

The η^3^-allyl complexes **1** and **2** were unreactive toward CO insertion. In contrast, the norbornyl derivatives **7c**,**d**,**f** afforded interesting amino acid or lactone derivatives upon CO insertion into the Pd–C bond and subsequent hydrolysis, under the appropriate conditions.

Overall, we have shown that the isolation of the organometallic intermediates obtained upon stepwise insertion of alkynes and alkenes into palladated phenethylamines allows the synthesis of functionalized primary phenethylamines. These stoichiometric routes avoid the problems associated with substrates that make difficult the development of catalytic processes (such as primary alkylamines) and allow the control of the regioselective insertion of the different unsaturated coupling partners.

## Experimental Section

*Caution! Special precautions should be taken in handling thallium(I) compounds, which are toxic*.

### General Procedures

Infrared spectra were recorded on a PerkinElmer 16F-PC-FT spectrometer. C, H, N, and S analyses were carried out with a LECO CHNS-932 microanalyzer. Conductance measurements and melting point determinations were carried out as described elsewhere.^41^ Unless stated otherwise, NMR spectra were recorded in CDCl_3_ with Bruker Avance 300, 400, and 600 spectrometers. Chemical shifts are referenced to TMS (^1^H and ^13^C{^1^H}). Signals in the ^1^H and ^13^C NMR spectra of all compounds were assigned with the help of APT, HMQC, and HMBC experiments. High-resolution electrospray ionization mass spectra (ESI-MS) were recorded on an Agilent 6220 Accurate-Mass time-of-flight (TOF) LC/MS. Reactions were carried out at room temperature without special precautions against moisture unless specified otherwise. The groups C_6_H_4_ and C_6_H_2_ are denoted by Ar. Free and inserted 2-norbornene are denoted by C_7_H_10_ (free), CH(C_5_H_8_)CH (inserted), and nor (both, NMR signals).

**Chart 2:**
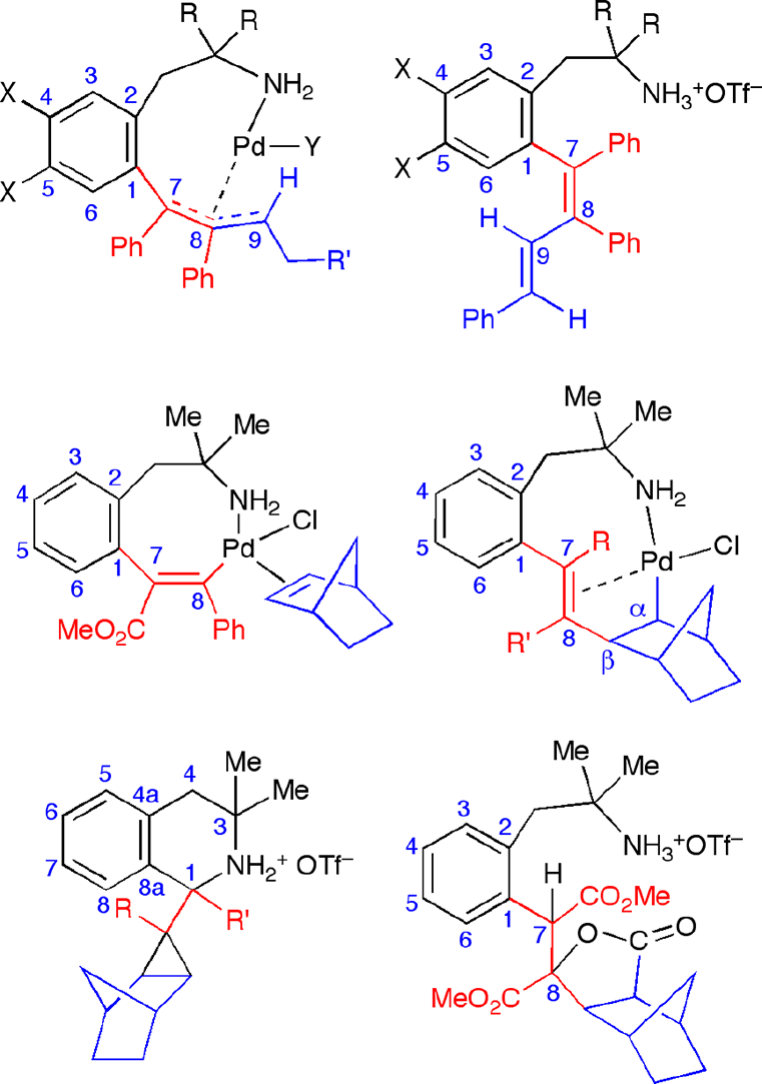
Numbering Schemes for the New Palladium(II) Complexes and the Organic Derivatives

Styrene, ethyl acrylate, 2-norbornene, dimethyl acetylenedicarboxylate (DMAD), methyl phenylpropiolate, *p*-toluidine (Tol), KO^t^Bu, and HOTf (HO_3_SCF_3_) were used as received from comertial sources. The palladacycles [Pd{*C,N*-C_6_H_2_(CH_2_CH_2_NH_2_)-2-(OMe)_2_-4,5}(μ-Br)]_2_ (**A**),^26^ [Pd{*C,N*-C_6_H_4_(CH_2_CMe_2_NH_2_)-2}(μ-Cl)]_2_ (**B**),^24^ [Pd{*C,N*-C(Ph)=C(Ph)C_6_H_2_(CH_2_CH_2_NH_2_)-2-(OMe)_2_-4,5}(μ-Br)]_2_ (**a**), [Pd{*C,N*-C(Ph)=C(R)C_6_H_4_(CH_2_CMe_2_NH_2_)-2}(μ-Cl)]_2_ (R = Ph (**b**), Me (**c**), CO_2_Me (**d**)),^20^ and [Pd{*C,N*-CH(C_5_H_8_)CHC_6_H_4_(CH_2_CMe_2_NH_2_)-2}(μ-Cl)]_2_ (**e**)^19^ were prepared as previously reported. TlOTf was prepared by the reaction of Tl_2_CO_3_ and HO_3_SCF_3_ (1/2) in water and recrystallized from acetone/Et_2_O. Chart 2 gives the numbering schemes for the new organometallic and organic derivatives.

### Synthesis of *anti-*/*syn*-**1a**

Styrene (23 μL, 0.202 mmol) was added to a solution of palladacycle **a** (110 mg, 0.100 mmol) in CH_2_Cl_2_ (10 mL), and the mixture was stirred for 20 h. The resulting mixture was filtered through a plug of Celite, the filtrate was concentrated to ca. 1 mL, and Et_2_O (15 mL) was added. The mixture was cooled to 0 °C, the resulting suspension was filtered, and the pale yellow solid was air-dried to give a mixture of *anti-*/*syn*-**1a**. Yield: 86 mg, 0.132 mmol, 66%. Mp: 143 °C. Anal. Calcd for C_32_H_32_BrNO_2_Pd (648.936): C, 59.23; H, 4.97; N, 2.16. Found: C, 59.32; H, 5.10; N, 2.19. IR (cm^–1^): ν(NH) 3319 m, 3258 w, 3206 m, 3135 w. The NMR spectra of this complex showed two sets of signals in approximately a 2.5/1 ratio (determined by ^1^H integration), corresponding to the mixture *anti-*/*syn*-**1a**. Data for *anti-***1a** (extracted from the mixture) are as follows. ^1^H NMR (300.1 MHz): δ 1.63–1.70 (m, 1 H, NH_2_), 1.88 (m, 1 H, CH_2_Ar), 2.56 (br dd, 1 H, CH_2_Ar, ^2^*J*_HH_ = 15.3, ^3^*J*_HH_ = 7.8 Hz), 2.76–2.85 (m, partially obscured by the resonance of MeO, 1 H, CH_2_Ph), 2.86 (s, 3 H, MeO), 2.97–3.02 (br d, 1 H, CH_2_N, ^2^*J*_HH_ = 12.6 Hz), 3.22–3.35 (m, 2 H, 1 H of CH_2_N + 1 H of NH_2_), 2.38 (br dd, 1 H, CH_2_Ph, ^2^*J*_HH_ = 17.4, ^3^*J*_HH_ = 4.5 Hz), 3.87 (s, 3 H, MeO), 5.11 (dd, 1 H, CH, ^3^*J*_HH_ = 12.3, ^3^*J*_HH_ = 4.5 Hz), 6.47 (s, 1 H, H6), 6.64 (s, 1 H, H3), 6.80–7.27 (m, 15 H, Ph). ^13^C{^1^H} NMR (75.5 MHz): δ 35.2 (CH_2_Ar), 39.1 (CH_2_Ph), 42.9 (CH_2_N), 54.7 (MeO), 55.9 (MeO), 85.0 (CH), 93.8 (C7), 111.9 (CH6), 116.1 (CH3), 123.6 (C8), 125.8 (CH, Ph), 126.8 (CH, Ph), 127.0 (CH, Ph), 127.3 (CH, Ph), 128.0 (CH, Ph), 128.4 (CH, Ph), 128.5 (CH, Ph), 128.6 (CH, Ph), 129.6 (CH, Ph), 132.2 (C1), 135.1 (*i-*C, Ph), 140.4 (*i-*C, Ph), 141.1 (*i-*C, Ph), 143.6 (C2), 146.4 (C5), 148.3 (C4).

### *anti*/*syn* Isomerization of **1a**

An NMR tube was charged with a 2.5:1 mixture of *anti*-/*syn*-**1a** (20 mg) and CDCl_3_ (0.6 mL), and the solution was heated at 60 °C. The sample was checked by ^1^H NMR periodically, until complete conversion. After 10 days at 60 °C, a spectroscopically pure sample of *syn*-**1a** was obtained. Data for *syn*-**1a** are as follows. ^1^H NMR (400.9 MHz): δ 1.61–1.67 (m, 1 H, NH_2_), 1.89 (br dd, 1 H, CH_2_Ar, ^2^*J*_HH_ = 15.3, ^3^*J*_HH_ = 10.8 Hz), 2.47 (br dd, 1 H, CH_2_Ar, ^2^*J*_HH_ = 15.4, ^3^*J*_HH_ = 8.0 Hz), 2.86–2.90 (m, 1 H, CH_2_Ph), 3.23 (dd, partially obscured by the resonance of NH_2_, 1 H, CH_2_N, ^2^*J*_HH_ = 12.0, ^3^*J*_HH_ = 8.0 Hz), 3.02 (br d, 1 H, NH_2_, ^2^*J*_HH_ = 12.0 Hz), 3.42 (s, 3 H, MeO), 3.46 (br dd, 1 H, CH_2_Ph, ^2^*J*_HH_ = 14.2, ^3^*J*_HH_ = 3.5 Hz), 3.87 (s, 3 H, MeO), 4.80 (dd, 1 H, CH, ^3^*J*_HH_ = 11.0, ^3^*J*_HH_ = 3.7 Hz), 6.39 (s, 1 H, H6), 6.60 (s, 1 H, H3), 6.19 (br d, 1 H, Ph, ^3^*J*_HH_ = 7.0 Hz), 6.80 (br d, 2 H, Ph, ^3^*J*_HH_ = 7.0 Hz), 6.90–7.23 (m, 10 H, Ph), 7.40 (m, 1 H, Ph), 7.85 (br d, 1 H, Ph, ^3^*J*_HH_ = 6.0 Hz). ^13^C{^1^H} NMR (75.5 MHz): δ 34.8 (CH_2_Ar), 35.8 (CH_2_Ph), 43.6 (CH_2_N), 55.6 (MeO), 60.0 (MeO), 84.5 (CH), 90.8 (C7), 110.0 (CH6), 116.2 (CH3), 124.0 (C8), 126.0 (CH, Ph), 127.0 (CH, Ph), 127.3 (CH, Ph), 127.7 (CH, Ph), 128.4 (CH, Ph), 128.5 (CH, Ph), 129.0 (CH, Ph), 131.8 (C1), 133.2 (*i-*C, Ph), 133.7 (CH, Ph), 135.1 (*i-*C, Ph), 140.5 (*i-*C, Ph), 142.4 (C2), 147.5 (C5), 148.2 (C4).

### Synthesis of *anti-*/*syn*-**1b**

#### Method A

Styrene (52 μL, 0.453 mmol) was added to a solution of palladacycle **b** (200 mg, 0.213 mmol) in CH_2_Cl_2_ (10 mL). The solution was stirred for 12 h, the solvent was concentrated to ca. 5 mL, Et_2_O (5 mL) was added, and the resulting suspension was filtered through a plug of Celite. The filtrate was concentrated to ca. 1 mL, and *n-*pentane (20 mL) was added. The suspension was filtered, and the pale yellow solid was washed with *n-*pentane (2 × 5 mL) and air-dried to give a mixture of *anti-*/*syn*-**1b** (ratio ca. 1.25:1 by ^1^H NMR). Yield: 155 mg, 0.270 mmol, 63%. Dec pt: 252 °C. Anal. Calcd for C_32_H_32_ClNPd (572.485): C, 67.14; H, 5.63; N, 2.44. Found: C, 67.19; H, 5.60; N, 2.39. IR (cm^–1^): ν(NH) 2295 w, 3238 w.

#### Method B

In a different preparation, it was possible to obtain two different crops by fractional crystallization, each of them enriched in one of the isomers. Styrene (30 μL, 0.262 mmol) was added to a solution of palladacycle **b** (120 mg, 0.128 mmol) in CH_2_Cl_2_ (10 mL). The mixture was stirred for 12 h and filtered through a plug of Celite. The filtrate was concentrated to ca. 3 mL, and Et_2_O (15 mL) was added. The resulting suspension was filtered, and the solid was air-dried to afford 57 mg of an *anti*-enriched mixture of both isomers (*anti*/*syn* = 3/1). The mother liquors were concentrated to ca. 3 mL, and *n*-pentane (20 mL) was added. The resulting suspension was filtered, and the solid was air-dried to afford 25 mg of a *syn*-enriched mixture of both isomers (*anti*/*syn* = 1/3). Almost spectroscopically pure samples of *anti*- and *syn*-**1b** could be obtained by growing single crystals from the enriched mixtures. Data for *anti*-**1b** are as follows. ^1^H NMR (300.1 MHz): δ 1.34 (s, 6 H, CMe_2_), 1.86 (br d, 1 H, NH_2_, ^2^*J*_HH_ = 10.8 Hz), 2.11 (d, 1 H, CH_2_Ar, ^2^*J*_HH_ = 15.0 Hz), 2.38 (br d, 1 H, CH_2_Ar, ^2^*J*_HH_ = 14.7 Hz), 2.59 (dd, 1 H, C*H*_*2*_Ph, ^2^*J*_HH_ = 16.5, ^3^*J*_HH_ = 11.7 Hz), 3.18 (br d, 1 H, NH_2_, ^2^*J*_HH_ = 10.8 Hz), 3.60 (dd, 1 H, C*H*_*2*_Ph, ^2^*J*_HH_ = 16.5, ^3^*J*_HH_ = 4.5 Hz), 5.16 (dd, 1 H, CH, ^3^*J*_HH_ = 11.7, ^3^*J*_HH_ = 4.5 Hz), 6.86–6.91 (m, 3 H, Ph + Ar), 7.04–7.34 (m, 16 H, Ph + Ar). ^13^C{^1^H} NMR (75.5 MHz): δ 27.8 (Me, CMe_2_), 36.0 (Me, CMe_2_), 39.0 (*C*H_2_Ph), 46.8 (CH_2_Ar), 52.3 (*C*Me_2_), 88.3 (CH), 89.4 (C7), 122.9 (C8), 125.9 (CH), 126.3 (CH), 126.7 (CH), 127.0 (CH), 127.5 (CH), 127.8 (CH, Ph), 127.9 (CH, Ph), 128.3 (br s, CH, Ph), 129.2 (CH), 129.4 (CH), 128.5 (CH, Ph), 132.3 (CH), 134.9 (CH3), 137.6 (C2), 139.8 (*i-*C, Ph), 140.4 (C1), 141.0 (*i-*C, Ph), 143.2 (*i-*C, Ph). Data for *syn*-**1b** are as follows. ^1^H NMR (400.9 MHz): δ 1.29 (s, 3 H, Me, CMe_2_), 1.30 (s, 3 H, Me, CMe_2_), 1.85 (br d, 1 H, NH_2_, ^2^*J*_HH_ = 10.0 Hz), 2.09 (d, 1 H, CH_2_Ar, ^2^*J*_HH_ = 14.8 Hz), 2.28 (dd, 1 H, CH_2_Ar, ^2^*J*_HH_ = 14.8, ^4^*J*_HH_ = 1.2 Hz), 3.17 (dd, 1 H, C*H*_*2*_Ph, ^2^*J*_HH_ = 14.8, ^3^*J*_HH_ = 10.6 Hz), 3.29 (br d, 1 H, NH_2_, ^2^*J*_HH_ = 10.4 Hz), 3.40 (dd, 1 H, C*H*_*2*_Ph, ^2^*J*_HH_ = 15.2, ^3^*J*_HH_ = 4.0 Hz), 4.84 (dd, 1 H, CH, ^3^*J*_HH_ = 10.4, ^3^*J*_HH_ = 4.0 Hz), 6.50 (br s, 1 H, Ph), 6.87–6.91 (m, 4 H, Ph), 6.99–7.04 (m, 5 H, H3 + H6 + 3 H of Ph), 7.08 (td, 1 H, H5, ^3^*J*_HH_ = 7.6, ^4^*J*_HH_ = 1.6 Hz), 7.11–7.20 (m, 6 H, Ph), 7.22 (td, 1 H, H4, ^3^*J*_HH_ = 7.2, ^4^*J*_HH_ = 1.6 Hz), 7.36 (br s, 1 H, Ph), 7.84 (br s, 1 H, Ph). ^13^C{^1^H} NMR (100.8 MHz): δ 27.2 (Me, CMe_2_), 35.1 (*C*H_2_Ph), 36.1 (Me, CMe_2_), 46.3 (CH_2_Ar), 53.1 (*C*Me_2_), 84.7 (CH), 88.0 (C7), 124.5 (C8), 125.8 (CH, Ph), 126.2 (CH6), 126.9 (CH5), 127.1 (CH, Ph), 127.28 (CH4 or Ph), 127.29 (CH4 or Ph), 127.5 (CH, Ph), 127.9 (br s, CH, Ph), 128.21 (CH, Ph), 128.25 (CH, Ph), 128.4 (CH, Ph), 128.7 (CH, Ph), 131.4 (CH, Ph), 133.6 (br s, CH, Ph), 134.9 (*i-*C, Ph), 135.1 (CH3), 138.4 (C2), 139.7 (*i-*C, Ph), 140.0 (C1), 142.2 (*i-*C, Ph).

### *anti*/*syn* Isomerization of **1b**

An NMR tube was charged with a 2.5/1 mixture of *anti-*/*syn*-**1b** (20 mg) and CDCl_3_ (0.6 mL), and the solution was heated at 60 °C. The sample was checked by ^1^H NMR periodically, until no change was observed. After 5 days at 60 °C, a 1/10 mixture of *anti-*/*syn*-**1b** was obtained.

### Synthesis of *anti-*/*syn*-**2a**

Ethyl acrylate (40 μL, 0.367 mmol) was added to a solution of palladacycle **a** (200 mg, 0.183 mmol) in CH_2_Cl_2_ (10 mL), and the mixture was stirred for 48 h. The resulting mixture was filtered through a plug of Celite, the filtrate was concentrated to ca. 1 mL, and Et_2_O (20 mL) was added. The suspension was filtered, and the pale yellow solid was washed with Et_2_O (2 × 5 mL) and air-dried to give an *anti-*/*syn*-**2a** mixture (171 mg; ratio ca. 5/1 by ^1^H NMR). The filtrate was concentrated to ca. 1 mL, and *n-*pentane (20 mL) was added. The resulting suspension was filtered, and the solid was air-dried to afford a *anti-*/*syn*-**2a** mixture (30 mg; ratio ca. 1/5 by ^1^H NMR). Yield: 201 mg, 0.312 mmol, 85%. Mp: 169 °C. Anal. Calcd for C_29_H_32_BrNO_4_Pd (644.901): C, 54.01; H, 5.00; N, 2.17. Found: C, 54.25; H, 5.00; N, 2.49. IR (cm^–1^): ν(NH) 3293 w, 3214 br; ν(CO) 1727 s, 1711 s. Data for *anti*-**2a** are as follows. ^1^H NMR (400.9 MHz): δ 1.19 (t, 3 H, *Me*CH_2_, ^3^*J*_HH_ = 7.2 Hz), 1.61 (br d, 1 H, NH_2_, ^2^*J*_HH_ = 12.0 Hz), 1.90 (dd, 1 H, CH_2_Ar, ^2^*J*_HH_ = 10.8, ^3^*J*_HH_ = 4.8 Hz), 2.35 (dd, 1 H, C*H*_*2*_CO_2_Et, ^2^*J*_HH_ = 17.3, ^3^*J*_HH_ = 11.6 Hz), 2.58 (br dd, 1 H, CH_2_Ar, ^2^*J*_HH_ = 15.6, ^4^*J*_HH_ = 6.8 Hz), 2.93 (m, 1 H, CH_2_N), 3.26–3.33 (m, 3 H, 1 H of NH_2_ + 1 H of CH_2_N + 1 H of C*H*_*2*_CO_2_Et), 3.86 (s, 3 H, MeO), 3.91 (s, 3 H, MeO), 4.01 (m, 1 H, CH_2_O), 4.15 (m, 1 H, CH_2_O), 4.85 (dd, 1 H, CH, ^3^*J*_HH_ = 11.6, ^3^*J*_HH_ = 4.8 Hz), 6.68 (s, 1 H, H3), 6.87 (s, 1 H, H6), 6.99–7.10 (m, 8 H, Ph), 7.11–7.25 (m, 2 H, Ph). ^13^C{^1^H} NMR (100.8 MHz): δ 14.1 (*Me*CH_2_), 35.1 (CH_2_Ar), 39.4 (*C*H_2_CO_2_Et), 42.9 (CH_2_N), 56.0 (MeO), 56.0 (MeO), 60.8 (CH_2_O), 79.1 (CH), 93.8 (C7), 112.5 (CH6), 116.4 (CH3), 123.9 (C8), 126.8 (CH), 127.2 (CH), 127.3 (CH), 127.9 (CH), 128.0 (CH), 129.6 (CH), 132.1 (C1), 132.6 (C2), 140.7 (*i-*C, Ph), 143.1 (*i-*C, Ph), 147.0 (C5), 148.6 (C4), 170.0 (CO).

### *anti*/*syn* Isomerization of **2a**

An NMR tube was charged with a 5/1 *anti-*/*syn*-**2a** mixture (20 mg) and CDCl_3_ (0.6 mL), and the solution was heated at 60 °C. The sample was checked by ^1^H NMR periodically, until complete conversion. After 48 h at 60 °C, a spectroscopically pure sample of *syn*-**2a** was obtained. Data for *syn*-**2a** are as follows. ^1^H NMR (300.1 MHz): δ 1.10 (t, 3 H, *Me*CH_2_, ^3^*J*_HH_ = 7.2 Hz), 1.67 (br d, 1 H, NH_2_, ^2^*J*_HH_ = 10.8 Hz), 1.88 (dd, 1 H, CH_2_Ar, ^2^*J*_HH_ = 15.6, ^3^*J*_HH_ = 10.8 Hz), 2.49 (dd, 1 H, CH_2_Ar, ^2^*J*_HH_ = 15.3, ^3^*J*_HH_ = 6.6 Hz), 2.80 (dd, 1 H, C*H*_*2*_CO_2_Et, ^2^*J*_HH_ = 18.0, ^3^*J*_HH_ = 10.2 Hz), 2.90 (m, partially obscured by the resonance of C*H*_*2*_CO_2_Et, 1 H, CH_2_N), 3.25–3.32 (m, 2 H, 1 H of CH_2_N + 1 H of C*H*_*2*_CO_2_Et), 3.37 (br d, 1 H, NH_2_, ^2^*J*_HH_ = 9.9 Hz), 3.89 (s, 3 H, MeO), 3.93–3.96 (m, partially obscured by the resonance of MeO, 1 H, CH_2_O), 3.96–4.05 (m, partially obscured by the resonance of MeO, 1 H, CH_2_O), 3.99 (s, 3 H, MeO), 4.75 (dd, 1 H, CH, ^3^*J*_HH_ = 10.2, ^3^*J*_HH_ = 4.2 Hz), 6.63 (s, 1 H, H3), 6.87–6.91 (m, partially obscured by the resonance of H6, 2 H, Ph), 6.93 (s, 1 H, H6), 7.01–7.17 (m, 6 H, Ph), 7.27–7.34 (m, 1 H, Ph), 7.65 (br d, 1 H, Ph, ^3^*J*_HH_ = 6.0 Hz). ^13^C{^1^H} NMR (75.5 MHz): δ 14.1 (*Me*CH_2_), 34.7 (CH_2_Ar), 34.9 (*C*H_2_CO_2_Et), 43.7 (CH_2_N), 56.0 (MeO), 56.2 (MeO), 60.4 (CH_2_O), 75.2 (CH), 92.2 (C7), 110.1 (CH6), 116.2 (CH3), 125.6 (C8), 127.1 (CH), 127.3 (CH), 127.6 (CH), 127.8 (CH), 128.8 (CH), 131.5 (C1), 133.0 (CH), 133.1 (C2), 134.6 (*i-*C, Ph), 142.2 (*i-*C, Ph), 147.8 (C5), 148.4 (C4), 170.8 (CO).

### Synthesis of *anti*-/*syn*-**2b**

#### Method A

Ethyl acrylate (50 μL, 0.460 mmol) was added to a solution of palladacycle **b** (200 mg, 0.213 mmol) in CH_2_Cl_2_ (10 mL). The solution was stirred for 12 h and then concentrated to ca. 5 mL. Et_2_O (5 mL) was added, and the resulting suspension was filtered through a plug of Celite. The filtrate was concentrated to ca. 1 mL, and *n-*pentane (20 mL) was added. The suspension was filtered, and the pale yellow solid was washed with *n-*pentane (2 × 5 mL) and air-dried to give as *anti-*/*syn*- mixture (ratio ca. 1.25/1 by ^1^H NMR). Yield: 197 mg, 0.346 mmol, 81%. Mp: 127 °C. Anal. Calcd for C_29_H_32_ClNO_2_Pd (568.451): C, 61.27; H, 5.67; N, 2.46. Found: C, 61.38; H, 5.42; N, 2.46. IR (cm^–1^): ν(NH) 3293 w, 3214 br; ν(CO) 1727 s, 1711 s.

#### Method B

In a different preparation, it was possible to obtain two different crops by fractional crystallization, each of them enriched in one of the isomers. Ethyl acrylate (40 μL, 0.262 mmol) was added to a solution of palladacycle **b** (150 mg, 0.160 mmol) in CH_2_Cl_2_ (10 mL). The mixture was stirred for 12 h and then filtered through a plug of Celite. The filtrate was concentrated to ca. 2 mL, and Et_2_O (15 mL) was added. The resulting suspension was filtered, and the solid was washed with Et_2_O (2 × 5 mL) and air-dried to afford 58 mg of a *syn*-enriched mixture of both isomers (*anti*/*syn* = 1/3). The mother liquors were concentrated to ca. 5 mL and cooled to 0 °C. A suspension formed, which was filtered, and the solid was air-dried to afford 60 mg of an *anti*-enriched mixture of both isomers (*anti*/*syn* = 3/1). Almost spectroscopically pure samples of *anti*- and *syn*-**2b** could be obtained by growing single crystals from the enriched mixtures. Data for *anti*-**2b** are as follows. ^1^H NMR (400.9 MHz): δ 1.22 (t, 3 H, *Me*CH_2_, ^3^*J*_HH_ = 7.2 Hz), 1.27 (s, 3 H, Me, CMe_2_), 1.32 (s, 3 H, Me, CMe_2_), 1.81 (br d, 1 H, NH_2_, ^2^*J*_HH_ = 10.0 Hz), 2.09 (d, 1 H, CH_2_Ar, ^2^*J*_HH_ = 14.8 Hz), 2.21 (dd, 1 H, C*H*_*2*_CH, ^2^*J*_HH_ = 17.2, ^3^*J*_HH_ = 11.2 Hz), 2.36 (dd, 1 H, CH_2_Ar, ^2^*J*_HH_ = 14.8, ^4^*J*_HH_ = 1.6 Hz), 3.13 (br d, partially obscured by the resonance of C*H*_*2*_CH, 1 H, NH_2_, ^2^*J*_HH_ = 9.6 Hz), 3.16 (dd, 1 H, C*H*_*2*_CH, ^2^*J*_HH_ = 17.2, ^3^*J*_HH_ = 5.2 Hz), 4.11 (m, 2 H, CH_2_O), 4.94 (dd, 1 H, CH, ^3^*J*_HH_ = 11.2, ^3^*J*_HH_ = 5.2 Hz), 6.86–6.90 (m, 2 H, Ph), 7.03–7.11 (m, 6 H, 5 H of Ph + H6), 7.13 (td, partially obscured by the resonance of H6, 1 H, H4, ^3^*J*_HH_ = 7.6, ^4^*J*_HH_ = 1.2 Hz), 7.21 (m, 2 H, *o*-H, Ph), 7.27 (m, partially obscured by the resonance of CHCl_3_, 1 H, *p*-H, Ph), 7.30 (dd, 1 H, H3, ^3^*J*_HH_ = 7.6, ^4^*J*_HH_ = 1.2 Hz), 7.34 (td, 1 H, H5, ^3^*J*_HH_ = 7.6, ^4^*J*_HH_ = 1.6 Hz). ^13^C{^1^H} NMR (100.8 MHz): δ 14.2 (*Me*CH_2_), 27.7 (Me, CMe_2_), 36.0 (Me, CMe_2_), 39.0 (*C*H_2_CH), 46.6 (CH_2_Ar), 52.3 (*C*Me_2_), 60.7 (CH_2_O), 81.0 (CH), 90.0 (C7), 123.5 (C8), 126.5 (CH5), 126.8 (*p*-CH, Ph), 127.1 (*o*-CH, Ph), 127.6 (*m*-CH, Ph), 127.9 (*m*-CH, Ph), 128.2 (CH4), 128.3 (*m*-CH, Ph), 128.9 (CH6), 129.1 (*o*-CH, Ph), 129.5 (*o*-CH, Ph), 132.2 (*p-*CH, Ph), 135.0 (CH3), 137.8 (C2), 140.49 (C1), 140.55 (*i-*C, Ph), 142.8 (*i-*C, Ph), 169.4 (CO). Data for *syn*-**2b** are as follows. ^1^H NMR (300.1 MHz): δ 1.16 (t, 3 H, *Me*CH_2_, ^3^*J*_HH_ = 7.2 Hz), 1.28 (s, 3 H, Me, CMe_2_), 1.30 (s, 3 H, Me, CMe_2_), 1.82 (br d, 1 H, NH_2_, ^2^*J*_HH_ = 9.9 Hz), 2.09 (d, 1 H, CH_2_Ar, ^2^*J*_HH_ = 14.7 Hz), 2.29 (dd, 1 H, CH_2_Ar, ^2^*J*_HH_ = 14.4, ^4^*J*_HH_ = 1.8 Hz), 2.76 (dd, 1 H, C*H*_*2*_CH, ^2^*J*_HH_ = 18.3, ^3^*J*_HH_ = 9.9 Hz), 3.17 (dd, 1 H, C*H*_*2*_CH, ^2^*J*_HH_ = 18.3, ^3^*J*_HH_ = 4.2 Hz), 3.22 (br d, partially obscured by the resonance of C*H*_*2*_CH, 1 H, NH_2_, ^2^*J*_HH_ = 9.3 Hz), 4.01 (m, 2 H, CH_2_O), 4.79 (dd, 1 H, CH, ^3^*J*_HH_ = 9.6, ^3^*J*_HH_ = 4.2 Hz), 6.87–7.36 (m, 13 H, Ph + Ar), 7.65 (br s, 1 H, CH, Ph or Ar). ^13^C{^1^H} NMR (75.5 MHz): δ 14.1 (*Me*CH_2_), 27.2 (Me, CMe_2_), 34.4 (*C*H_2_CH), 36.1 (Me, CMe_2_), 46.3 (CH_2_Ar), 53.4 (*C*Me_2_), 60.5 (CH_2_O), 76.7 (CH), 88.9 (C7), 125.5 (C8), 126.6 (CH), 127.1 (CH), 127.3 (CH), 127.5 (CH), 127.6 (CH), 127.9 (CH), 128.3 (CH), 128.5 (br s, CH), 128.8 (CH), 129.5 (CH), 131.2 (CH), 133.0 (br s, CH), 134.5 (*i-*C, Ph), 135.1 (CH3), 138.3 (C2), 139.8 (C1), 142.0 (*i-*C, Ph), 170.4 (CO).

### *anti*/*syn* Isomerization of **2b**

An NMR tube was charged with a 1.25/1 *anti-*/*syn*-**1b** mixture (20 mg) and CDCl_3_ (0.6 mL), and the solution was heated at 60 °C. The sample was checked by ^1^H NMR periodically, until no change was observed. After 48 days at 60 °C, a 1/20 *anti-*/*syn*-**2b** mixture was obtained.

### Synthesis of *anti*-/*syn*-**3b**

TlOTf (56 mg, 0.158 mmol) was added to a suspension of complex **1b** (90 mg, 0.157 mmol) in acetone (10 mL), and the resulting mixture was stirred for 2 h. The solvent was removed, and CH_2_Cl_2_ (15 mL) was added. The suspension was filtered through a plug of Celite, *p*-toluidine (17 mg, 0.159 mmol) was added to the filtrate, and the mixture was stirred for another 30 min. The solvent was concentrated to ca. 1 mL, and *n*-pentane was added. The suspension was filtered, and the pale yellow solid was washed with cold *n*-pentane (2 × 5 mL) and air-dried to give an *anti-*/*syn*-**3b** mixture (ratio ca. 1/1.33 by ^1^H NMR). Yield: 60.0 mg, 0.076 mmol, 48%. Mp: 120 °C. Λ_M_ (Ω^–1^ mol^–1^ cm^2^): 106 (*c* = 2.7 × 10^–4^ M). Anal. Calcd for C_40_H_41_F_3_N_2_O_3_PdS (793.237): C, 60.57; H, 5.21; N, 3.53; S, 4.04. Found: C, 60.38; H, 5.17; N, 3.30; S, 4.04. IR (cm^–1^): ν(NH) 3295 w, 3251 m, 3159 w. Data for *anti*-**3b** (minor isomer, extracted from the mixture) are as follows. δ 0.86 (s, 3 H, Me, CMe_2_), 1.27 (s, 3 H, Me, CMe_2_), 1.92 (br d, 1 H, NH_2_, ^2^*J*_HH_ = 12.6 Hz), 1.98 (d, 1 H, CH_2_, ^2^*J*_HH_ = 15.2 Hz), 2.20–2.29 (m, 1 H, CH_2_, overlapped with one H of CH_2_ of the major isomer), 2.29 (s, 3 H, Me, Tol), 2.44–2.55 (m, 1 H, CH_2_, overlapped with one H of CH_2_ of the major isomer), 2.73 (dd, 1 H, CH_2_, ^2^*J*_HH_ = 14.8, ^3^*J*_HH_ = 9.2 Hz), 4.13 (br d, 1 H, NH_2_, ^2^*J*_HH_ = 11.2 Hz), 4.74 (dd, 1 H, CH, ^3^*J*_HH_ = 8.8, ^3^*J*_HH_ = 6.0 Hz), 5.33 (br d, 1 H, NH_2_, ^2^*J*_HH_ = 11.2 Hz), 5.54 (br d, 1 H, NH_2_, ^2^*J*_HH_ = 10.4 Hz), 6.32–7.55 (m, 23 H, Ph + Ar, overlapped with the aromatic protons of the major isomer). Data for *syn*-**3b** (major isomer, extracted from the mixture) are as follows. ^1^H NMR (400.9 MHz, −40 °C): δ 1.45 (s, 3 H, Me, CMe_2_), 1.48 (s, 3 H, Me, CMe_2_), 1.70 (br d, 1 H, NH_2_, ^2^*J*_HH_ = 10.8 Hz), 2.06 (d, 1 H, CH_2_, ^2^*J*_HH_ = 15.2 Hz), 2.20–2.29 (m, 1 H, CH_2_, overlapped with one H of CH_2_ of the minor isomer), 2.33 (s, 3 H, Me, Tol), 2.44–2.55 (m, 1 H, CH_2_, overlapped with one H of CH_2_ of the minor isomer), 2.81 (dd, 1 H, CH_2_Ar, ^2^*J*_HH_ = 16.4, ^3^*J*_HH_ = 4.0 Hz), 3.92 (d, 1 H, NH_2_, ^2^*J*_HH_ = 9.6 Hz), 4.24 (dd, 1 H, CH, ^3^*J*_HH_ = 10.0, ^3^*J*_HH_ = 4.0 Hz), 5.02 (br d, 1 H, NH_2_, ^2^*J*_HH_ = 11.2 Hz), 6.30 (br d, 1 H, NH_2_, ^2^*J*_HH_ = 10.0 Hz), 6.32–7.55 (m, 23 H, Ph + Ar, overlapped with the aromatic protons of the minor isomer). Data for both isomers are as follows. ^13^C{^1^H} NMR (100.8 MHz, −40 °C): δ 20.7 (Me, Tol, major), 20.8 (Me, Tol, minor), 25.9 (Me, CMe_2_, minor), 27.4 (Me, CMe_2_, major), 33.8 (CH_2_, minor), 34.4 (Me, CMe_2_, major), 34.8 (Me, CMe_2_, minor), 41.4 (CH_2_, major), 46.5 (CH_2_, minor), 47.2 (CH_2_, major), 52.6 (*C*Me_2_, major), 53.0 (*C*Me_2_, minor), 84.6 (CH, minor), 86.6 (C, major), 87.1 (C, minor), 93.7 (CH, major), 188.8 (CH, major), 119.8 (CH, minor), 119.9 (q, CF_3_, ^1^*J*_CF_ = 318.8 Hz), 124.1 (C, major), 126.3 (CH), 126.4 (CH), 126.5 (CH), 126.7, 126.8 (C, minor), 127.1 (CH), 127.2 (CH), 127.3 (CH), 127.4 (CH), 127.6 (CH), 127.7 (CH), 127.8 (CH), 127.8 (CH), 127.9 (CH), 128.0 (CH), 128.1 (CH), 128.1 (CH), 128.4 (CH), 128.6 (CH), 128.7 (CH), 129.0 (CH), 129.5 (CH), 129.7 (CH), 129.8 (CH), 130.0 (CH), 131.5 (CH), 131.9 (CH), 132.7 (CH), 133.1 (C, major), 133.6 (C, minor), 134.1 (C, minor), 134.9 (CH), 135.0 (CH), 137.7 (C, major), 138.2 (C, minor), 138.5 (C, minor), 138.9 (C, minor), 139.0 (C, major), 139.4 (C, minor), 139.6 (C, major), 139.7 (C, major), 139.9 (C, major), 140.8 (C, minor), 141.2 (C, major).

### Synthesis of **4a**·1/2H_2_O

In a Carius tube, KO^t^Bu (190 mg, 1.55 mmol) was added to a solution of *anti*-/*syn-***1a** (100 mg, 0.154 mmol) in dry toluene (10 mL), under a nitrogen atmosphere. The mixture was heated at 100 °C for 12 h. Decomposition to metallic palladium was observed. The solvent was removed, and CH_2_Cl_2_ (20 mL) was added to the residue. The resulting suspension was filtered through a plug of Celite, the filtrate was concentrated to ca. 1 mL, *n*-pentane (20 mL) was added, and the mixture was cooled to 0 °C. The resulting suspension was filtered, and the solid was air-dried to give **4a·**1/2H_2_O as a colorless solid. Yield: 55 mg, 0.117 mmol, 76%. Mp: 129 °C. Anal. Calcd for C_32_H_31_NO_2_·1/2H_2_O (470.611): C, 81.67; H, 6.85; N, 2.97. Found: C, 81.84; H, 6.60; N, 3.05. ESI-HRMS (*m*/*z*): exact mass calcd for C_32_H_32_NO_2_, 462.2433 [(M + H)^+^]; found, 462.2433. IR (cm^–1^): ν(NH) 3375 w. ^1^H NMR (300.1 MHz): δ 1.22 (br s, 3 H, NH_2_ + 1 H of H_2_O), 2.50–2.61 (m, 2 H, CH_2_Ar), 2.63–2.70 (m, 1 H, CH_2_N), 2.70–2.83 (m, 1 H, CH_2_N), 3.86 (s, 3 H, MeO), 3.95 (s, 3 H, MeO), 6.23 (d, 1 H, CH = , ^3^*J*_HH_ = 15.9 Hz), 6.80 (s, 1 H, H3), 6.81 (s, 1 H, H6), 6.86–6.90 (m, 2 H, Ph), 6.93 (d, 1 H, CH = , ^3^*J*_HH_ = 15.9 Hz), 6.97–7.00 (m, 3 H, Ph), 7.12–7.24 (m, 7 H, Ph), 7.27–7.35 (m, 3 H, Ph). ^13^C{^1^H} NMR(100.8 MHz): δ 37.4 (CH_2_Ar), 42.8 (CH_2_N), 55.8 (MeO), 56.1 (MeO), 111.9 (CH3), 112.6 (CH6), 126.3 (CH, Ph), 126.4 (CH, Ph), 126.9 (CH, Ph), 127.3 (CH, Ph), 127.4 (CH, Ph), 128.1 (CH, Ph), 128.5 (CH, Ph), 130.5 (CH, Ph), 130.9 (CH = ), 131.2 (C2), 131.3 (CH, Ph), 132.3 (CH = ), 133.8 (C1), 137.5 (*i*-C, Ph), 139.7 (*i*-C, Ph), 139.7 (*i*-C, Ph), 141.3 (C7), 141.8 (C8), 147.0 (C4), 148.2 (C5).

### Synthesis of **5b**·H_2_O

In a Carius tube, KO^t^Bu (250 mg, 2.04 mmol) was added to a solution of *anti*-/*syn-***1b** (120 mg, 0.209 mmol) in dry toluene (10 mL), under a nitrogen atmosphere. The mixture was heated at 100 °C for 12 h. Decomposition to metallic palladium was observed. The solvent was removed, and Et_2_O (20 mL) was added to the residue. The suspension was filtered through a plug of Celite, and the solvent was removed from the filtrate to give crude **4b** as an oily residue, which was was characterized by ^1^H NMR. Data for **4b** are as follows. ^1^H NMR (400.9 MHz): δ 0.99 (br s, 2 H, NH_2_), 1.04 (s, 3 H, Me, CMe_2_), 1.09 (s, 3 H, Me, CMe_2_), 2.34, 2.54 (AB system, 2 H, CH_2_Ar, ^2^*J*_AB_ = 13.6 Hz), 6.20 (d, 1 H, CH = , ^3^*J*_HH_ = 15.6 Hz), 6.81–6.85 (m, 2 H), 6.96–7.00 (m, 3 H), 7.03 (d, 1 H, CH=, ^3^*J*_HH_ = 16.0 Hz), 7.10–7.47 (m, 14 H). Crude **4b** was dissolved in CH_2_Cl_2_ (5 mL), HOTf (0.05 mL, 0.565 mmol) was added, and the resulting solution was stirred for 30 min. The solvent was concentrated to ca. 1 mL, Et_2_O (20 mL) was added, and the mixture was cooled to 0 °C. The resulting suspension was filtered, and the solid was washed with Et_2_O (2 × 5 mL) and air-dried to give the salt **5b·**H_2_O as a colorless solid. Yield: 63 mg, 0.105 mmol, 50%. Mp: 131 °C. Λ_M_ (Ω^–1^ mol^–1^ cm^2^): 108.5 (*c* = 5.09 × 10^–4^ M). Anal. Calcd for C_33_H_32_F_3_NO_3_S·H_2_O (597.696): C, 66.31; H, 5.73; N, 2.34; S, 5.36. Found: C, 66.02; H, 5.59; N, 2.43; S, 5.59. ESI-HRMS (*m*/*z*): exact mass calcd for C_32_H_32_N, 430.2529 [(M – CF_3_SO_3_)^+^]; found, 430.2533. IR (cm^–1^): ν(NH) 3479 w. ^1^H NMR (400.9 MHz): δ 1.23 (s, 3 H, Me, CMe_2_), 1.25 (s, 3 H, Me, CMe_2_), 2.55, 2.62 (AB system, 2 H, CH_2_Ar, ^2^*J*_AB_ = 14.0 Hz), 3.00 (br s, 2 H, H_2_O), 6.22 (d, 1 H, CH=, ^3^*J*_HH_ = 16.0 Hz), 6.75–6.95 (m, 4 H, Ph), 6.94–6.97 (m, 2 H, Ph), 6.99 (d, 1 H, CH = , ^3^*J*_HH_ = 16.0 Hz), 7.09–7.21 (m, 6 H, Ph), 7.22–7.27 (m, 4 H, 3 H of Ph + H3), 7.33 (td, 1 H, H4, ^3^*J*_HH_ = 7.6, ^4^*J*_HH_ = 1.2 Hz), 7.38 (td, 1 H, H5, ^3^*J*_HH_ = 7.2, ^4^*J*_HH_ = 1.2 Hz), 7.50 (dd, 1 H, H6, ^3^*J*_HH_ = 7.2, ^4^*J*_HH_ = 1.2 Hz), 7.70 (br s, 3 H, NH_3_). ^13^C{^1^H} NMR (100.8 MHz): δ 24.7 (Me, CMe_2_), 26.4 (Me, CMe_2_), 42.4 (CH_2_Ar), 57.0 (*C*Me_2_), 119.7 (q, CF_3_, ^1^*J*_CF_ = 318.7 Hz), 126.5 (CH, Ph), 127.0 (CH, Ph), 127.3 (CH5), 127.6 (CH, Ph), 128.1 (CH, Ph), 128.5 (CH, Ph), 128.6 (CH4), 130.3 (CH, Ph), 130.6 (CH = ), 130.9 (CH, Ph), 132.2 (CH3), 133.4 (CH = ), 133.9 (C2), 134.2 (CH6), 137.3 (*i*-C, Ph), 139.4 (*i*-C, Ph), 140.5 (C8), 141.26 (*i*-C, Ph), 141.34 (C7), 142.2 (C1).

### Synthesis of [Pd{*C,N*-C(Ph)=C(CO_2_Me)C_6_H_4_CH_2_CMe_2_NH_2_-2}Cl(C_7_H_10_)] (**6d**)

2-Norbornene (C_7_H_10_; 25 mg, 0.265 mmol) was added to a solution of palladacycle **d** (65 mg, 0.072 mmol) in CH_2_Cl_2_ (10 mL), and the yellow solution was stirred for 2 h. The solvent was removed under vacuum at room temperature (to prevent the evolution of the complex), and Et_2_O (10 mL) was added. The resulting suspension was filtered, and the solid was washed with Et_2_O (2 × 5 mL) and air-dried to afford complex **6d** as a colorless solid. Yield: 58 mg, 0.106 mmol, 73%. Mp: 182 °C dec. Anal. Calcd for C_27_H_32_ClNO_2_Pd (544.429): C, 59.56; H, 5.92; N, 2.57. Found: C, 59.37; H, 6.01; N, 2.41. IR (cm^–1^): ν(NH) 3323 w, 3267 w; *n*(CO) 1725 s. ^1^H NMR (400.9 MHz): δ 0.66 (br d, 1 H, CH, nor, ^3^*J*_HH_ = 8.0 Hz), 0.97 (br d, 1 H, CH_2_, nor, ^2^*J*_HH_ = 9.6 Hz), 1.04 (br m, 1 H, CH_2_, nor), 1.11 (br m, 1 H, CH_2_, nor), 1.35 (s, 3 H, Me, CMe_2_), 1.38 (m, partially obscured by the resonance of Me group, 2 H, CH_2_, nor), 1.40 (s, 3 H, Me, CMe_2_), 1.79 (br s, 1 H, CH, nor), 2.05 (br d, 1 H, CH_2_, nor, ^2^*J*_HH_ = 10.0 Hz), 2.49 (d, 1 H, CH_2_Ar, ^2^*J*_HH_ = 15.6 Hz), 2.67 (d, 1 H, CH_2_Ar, ^2^*J*_HH_ = 15.2 Hz), 3.08 (br s, 1 H, CH, nor), 3.26 (s, 3 H, MeO), 4.36 (d, 1 H, CH, nor, ^3^*J*_HH_ = 7.6 Hz), 7.16 (dd, 1 H, H3, ^3^*J*_HH_ = 7.6, ^4^*J*_HH_ = 1.2 Hz), 7.23–7.35 (m, 6 H, Ar + Ph), 7.83 (d, 1 H, H6, ^3^*J*_HH_ = 7.2 Hz), 7.95 (m, 1 H, *o*-H, Ph). The ^1^H resonance corresponding to the NH_2_ group was not observed. ^13^C{^1^H} NMR (75.5 MHz): δ 18.0 (CH, nor), 27.6 (Me, CMe_2_), 27.8 (CH_2_, nor), 28.9 (CH_2_, nor), 36.5 (Me, CMe_2_), 36.9 (CH_2_, nor), 40.4 (CH, nor), 40.6 (CH, nor), 47.1 (CH_2_Ar), 51.1 (CH, nor), 52.7 (MeO), 54.9 (*C*Me_2_), 99.8 (C7), 123.9 (CH6), 126.8 (CH, Ph), 127.0 (CH, Ph), 127.3 (CH, Ph), 127.4 (CH, Ph), 127.5 (CH5), 127.9 (CH4), 134.0 (C1), 134.1 (*o*-CH, Ph), 135.1 (CH3), 137.2 (*i*-C or C8), 138.4 (C2), 170.0 (CO).

### Synthesis of [Pd{*C,N*-CH(C_5_H_8_)CHC(Ph)=C(Me)C_6_H_4_CH_2_CMe_2_NH_2_-2}Cl]·CHCl_3_ (**7c**·CHCl_3_)

In a Carius tube, 2-norbornene (100 mg, 1.06 mmol) was added to a solution of palladacycle **c** (250 mg, 0.308 mmol) in CHCl_3_ (15 mL), and the mixture was heated at 65 °C for 12 h. The resulting solution was filtered through a plug of Celite, the solvent was removed from the filtrate, and Et_2_O (20 mL) was added. The resulting suspension was filtered, and the solid was washed with Et_2_O (2 × 5 mL) and air-dried to give complex **7c**·CHCl_3_ as a yellow solid. Yield: 258 mg, 0.416 mmol, 67%. Dec pt: 223 °C. Anal. Calcd for C_26_H_32_ClNPd·CHCl_3_ (619.796): C, 52.32; H, 5.37; N, 2.26. Found: C, 52.57; H, 5.48; N, 2.29. IR (cm^–1^): ν(NH) 3313 w, 3250 w. ^1^H NMR (300.1 MHz): δ 0.82 (br d, 1 H, NH_2_, ^2^*J*_HH_ = 9.6 Hz), 0.95 (br d, 1 H, CH_2_, nor, ^2^*J*_HH_ = 10.2 Hz), 1.07 (br d, 1 H, Hα, ^3^*J*_HH_ = 7.5 Hz), 1.12 (s, 3 H, Me, CMe_2_), 1.20–1.27 (m, 2 H, CH_2_, nor), 1.37–1.50 (m, 2 H, CH_2_, nor), 1.64 (s, 3 H, Me, CMe_2_), 2.05 (br d, 1 H, CH_2_, nor, ^2^*J*_HH_ = 9.9 Hz), 2.14 (br s, 1 H, CH, nor), 2.20 (br d, 1 H, NH_2_, ^2^*J*_HH_ = 9.6 Hz), 2.35 (s, 3 H, MeC=), 2.57 (d, 1 H, CH_2_Ar, ^2^*J*_HH_ = 12.9 Hz), 3.09 (br s, 1 H, CH, nor), 3.65 (d, 1 H, Hβ, ^3^*J*_HH_ = 7.8 Hz), 3.96 (d, 1 H, CH_2_Ar, ^2^*J*_HH_ = 12.9 Hz), 6.67 (d, 1 H, H6, ^3^*J*_HH_ = 7.8 Hz), 6.76 (m, 1 H, H5), 7.01 (m, 2 H, H4 + H3), 7.18–7.25 (br m, partially obscured by the resonance of CHCl_3_, 3 H, *p*-H + *m*-H, Ph), 2.26 (s, 1 H, CHCl_3_), 7.42 (br d, 2 H, *o-*H, Ph, ^3^*J*_HH_ = 7.8 Hz). ^13^C{^1^H} NMR (75.5 MHz): δ 21.5 (CHα), 27.9 (*Me*C=), 28.5 (CH_2_, nor), 28.9 (CH_2_, nor), 30.7 (Me, CMe_2_), 30.9 (Me, CMe_2_), 37.3 (CH_2_, nor), 40.0 (CH, nor), 41.1 (CH, nor), 48.1 (CH_2_Ar), 52.3 (*C*Me_2_), 54.6 (CHβ), 77.8 (C8), 107.7 (C7), 126.3 (CH5), 126.7 (CH4), 126.8 (*p-*CH, Ph), 131.0 (br s, CH6 or *o-*CH of Ph), 131.1 (CH6 or *o-*CH of Ph), 132.7 (CH3), 135.8 (C2), 138.6 (*i*-C, Ph), 142.6 (C1). The ^13^C resonance corresponding to *m-*CH of Ph was overlapped with the signal of CH6 or *o*-CH.

### Synthesis of [Pd{*C,N*-CH(C_5_H_8_)CHC(Ph)=C(CO_2_Me)C_6_H_4_CH_2_CMe_2_NH_2_-2}Cl]·1/2CHCl_3_ (**7d**·1/2CHCl_3_)

#### Method A

In a Carius tube, methyl phenylpropiolate (48 μL, 0.324 mmol) was added to a solution of palladacycle **e** (120 mg, 0.156 mmol) in CHCl_3_ (10 mL), and the mixture was heated at 65 °C for 2 h. The yellow solution was concentrated to ca. 1 mL, and Et_2_O was added (20 mL). The suspension was filtered, and the solid was washed with Et_2_O (2 × 5 mL) and air-dried to give complex **7d**·1/2CHCl_3_ as a pale yellow solid. Yield: 37 mg, 0.061 mmol, 20%. The filtrate was contentrated to ca. 2 mL, and *n*-pentane (20 mL) was added. The suspension was filtered, and the yellow solid was air-dried to give a ca. 1/0.7 mixture of complex **7d** and palladacycle **d** (80 mg).

#### Method B

In a Carius tube, 2-norbornene (50 mg, 0.531 mmol) was added to a solution of palladacycle **d** (200 mg, 0.223 mmol) in CHCl_3_ (15 mL), and the mixture was heated at 65 °C for 4 h. The resulting solution was filtered through a plug of Celite, the filtrate was concentrated to ca. 1 mL, and Et_2_O (20 mL) was added. The suspension was filtered, and the solid was washed with Et_2_O (2 × 5 mL) and air-dried to give complex **7d**·1/2CHCl_3_ as a pale yellow solid. Yield: 198 mg, 0.328 mmol, 73%. Dec pt: 204 °C. Anal. Calcd for C_27_H_32_ClNO_2_Pd·1/2CHCl_3_ (604.117): C, 54.68; H, 5.42; N, 2.32. Found: C, 54.64; H, 5.42; N, 2.29. IR (cm^–1^): ν(NH) 3320 w, 3266 w; ν(CO) 1716 s. ^1^H NMR (400.9 MHz): δ 0.83 (br d, 1 H, NH_2_, ^2^*J*_HH_ = 9.6 Hz), 0.95 (br d, 1 H, CH_2_, nor, ^2^*J*_HH_ = 10.0 Hz), 1.05 (br d, 1 H, CH_2_, nor, ^2^*J*_HH_ = 10.4 Hz), 1.13 (s, 3 H, Me, CMe_2_), 1.19–1.23 (m, 2 H, Hα + 1 H of CH_2_ of nor), 1.35–1.45 (m, 2 H, CH_2_, nor), 1.64 (s, 3 H, Me, CMe_2_), 1.93 (d, 1 H, CH_2_, nor, ^2^*J*_HH_ = 9.6 Hz), 2.15 (br s, 1 H, CH, nor), 2.37 (br d, 1 H, NH_2_, ^2^*J*_HH_ = 9.6 Hz), 2.45 (d, 1 H, CH_2_Ar, ^2^*J*_HH_ = 13.2 Hz), 3.18 (br s, 1 H, CH, nor), 3.61 (d, 1 H, Hβ, ^3^*J*_HH_ = 8.0 Hz), 3.76 (s, 3 H, MeO), 4.35 (d, 1 H, CH_2_Ar, ^2^*J*_HH_ = 12.8 Hz), 6.83 (m, 1 H, H5), 6.88 (br d, 1 H, H6, ^3^*J*_HH_ = 7.2 Hz), 7.01 (br d, 1 H, H3, ^3^*J*_HH_ = 7.2 Hz), 7.08 (td, 1 H, H4, ^3^*J*_HH_ = 7.6, ^4^*J*_HH_ = 1.2 Hz), 7.28 (br m, partially obscured by the resonance of CHCl_3_, 3 H, *p*-H + *m*-H, Ph), 7.48 (br s, 2 H, *o-*H, Ph). ^13^C{^1^H} NMR (100.8 MHz): δ 23.8 (CHα), 28.3 (CH_2_, nor), 28.4 (CH_2_, nor), 30.7 (Me, CMe_2_), 30.75 (Me, CMe_2_), 37.0 (CH_2_, nor), 39.8 (CH, nor), 41.2 (CH, nor), 47.4 (CH_2_Ar), 52.2 (*C*Me_2_), 53.3 (MeO), 54.5 (CHβ), 101.5 (C7), 126.6 (CH5), 127.5 (*p-*CH, Ph), 127.7 (br s, *m-*CH, Ph), 127.9 (CH4), 130.5 (*o-*CH, Ph), 132.5 (CH3), 133.2 (CH6), 135.1 (C1), 136.2 (*i*-C, Ph), 137.6 (C2), 169.1 (CO). The resonance corresponding to C8 was not observed.

### Synthesis of [Pd{*C,N*-CH(C_5_H_8_)CHC(CO_2_Me)=C(CO_2_Me)C_6_H_4_CH_2_CMe_2_NH_2_-2}Cl] (**7f**)

#### Method A

In a Carius tube, dimethyl acetylenedicarboxylate (82 μL, 0.667 mmol) was added to a suspension of palladacycle **e** (240 mg, 0.312 mmol) in CHCl_3_ (15 mL), and the mixture was heated at 65 °C for 8 h. The resulting solution was filtered through a plug of Celite, the filtrate was concentrated to ca. 1 mL, and Et_2_O (20 mL) was added. The suspension was filtered, and the solid was washed with Et_2_O (2 × 5 mL) and air-dried to give the complex **7f** as a bright yellow solid. Yield: 247 mg, 0.469 mmol, 75%.

#### Method B

In a Carius tube, a solution of dimethyl acetylenedicarboxylate (65 μL, 0.529 mmol) and 2-norbornene (50 mg, 0.531 mmol) in CHCl_3_ (5 mL) was added to a solution of palladacycle **B** (150 mg, 0.312 mmol) in CHCl_3_ (15 mL), and the mixture was heated at 65 °C for 8 h. The resulting solution was filtered through a plug of Celite, the filtrate was concentrated to ca. 2 mL, and Et_2_O (20 mL) was added. The suspension was filtered, and the solid was washed with Et_2_O (2 × 5 mL) and air-dried to give complex **7f** as a bright yellow solid. Yield: 90 mg, 0.171 mmol, 33%. Dec pt: 188 °C. Anal. Calcd for C_23_H_30_ClNO_4_Pd (526.368): C, 52.48; H, 5.74; N, 2.66. Found: C, 52.21; H, 6.11; N, 2.67. IR (cm^–1^): ν(NH) 3326 m, 3267 m; ν(CO) 1731 s, 1716 s. ^1^H NMR (400.9 MHz): δ 1.08 (br d, partially obscured by the resonance of Me, 1 H, Hα, ^3^*J*_HH_ = 8.0 Hz), 1.10 (s, 3 H, Me, CMe_2_), 1.11 (m, partially obscured by the resonance of Me, 1 H, CH_2_, nor), 1.21 (m, 1 H, CH_2_, nor), 1.33 (br d, 1 H, CH_2_, nor, ^2^*J*_HH_ = 10.4 Hz), 1.44 (m, 1 H, CH_2_, nor), 1.51 (s, 3 H, Me, CMe_2_), 1.58 (m, 1 H, CH_2_, nor), 1.62 (br d, 1 H, NH_2_, ^2^*J*_HH_ = 10.4 Hz), 2.07 (br d, 1 H, NH_2_, ^2^*J*_HH_ = 10.4 Hz), 2.26 (br d, 1 H, CH_2_, nor, ^2^*J*_HH_ = 10.4 Hz), 2.48 (dd, 1 H, CH_2_Ar, ^2^*J*_HH_ = 13.2, ^4^*J*_HH_ = 1.2 Hz), 2.70 (br d, 1 H, CH, nor, ^3^*J*_HH_ = 3.6 Hz), 3.21 (br d, 1 H, CH, nor, ^3^*J*_HH_ = 4.0 Hz), 3.60 (d, partially obscured by the resonance of MeO, 1 H, Hβ, ^3^*J*_HH_ = 8.4 Hz), 3.61 (s, 3 H, MeO), 3.75 (s, 3 H, MeO), 4.12 (d, 1 H, CH_2_Ar, ^2^*J*_HH_ = 13.2 Hz), 7.13 (d, 1 H, H3, ^3^*J*_HH_ = 7.6 Hz), 7.20 (m, 2 H, H6 + H5), 7.28–7.33 (m, 1 H, H4). ^13^C{^1^H} NMR (100.8 MHz): δ 25.6 (CHα), 27.6 (CH_2_, nor), 28.3 (CH_2_, nor), 30.4 (Me, CMe_2_), 30.6 (Me, CMe_2_), 38.1 (CH_2_, nor), 39.2 (CH, nor), 42.4 (CH, nor), 47.7 (CH_2_Ar), 52.2 (*C*Me_2_), 52.5 (MeO), 53.3 (MeO), 53.4 (CHβ), 68.3 (C8), 102.9 (C7), 127.4 (CH5), 128.9 (CH4), 130.4 (CH6), 133.1 (CH3), 135.2 (C1), 137.2 (C2), 166.9 (CO), 167.7 (CO).

### Synthesis of **8f**

In a Carius tube, KO^t^Bu (50 mg, 0.445 mmol) was added to a solution of complex **7f** (120 mg, 0.228 mmol) in dry CH_3_CN, under an N_2_ atmosphere (10 mL), and the mixture was heated at 78 °C for 3 days. Decomposition to metallic palladium was observed. The solvent was removed, *n*-pentane (20 mL) was added, and the mixture was filtered through a plug of Celite. The solvent was removed from the filtrate, the residue was dissolved in Et_2_O (10 mL), and HOTf (0.01 mL, 0.113 mmol) was added. The resulting suspension was filtered, and the solid was washed with Et_2_O (2 × 2 mL) and air-dried to give compound **8f** as a colorless solid. Yield: 23 mg, 0.043 mmol, 19%. Mp: 197 °C. Λ_M_ (Ω^–1^ mol^–1^ cm^2^): 104.0 (*c* = 3.82 × 10^–4^ M). Anal. Calcd for C_24_H_30_F_3_NO_7_S (533.566): C, 54.02; H, 5.67; N, 2.62, S; 6.00. Found: C, 53.88; H, 6.09; N, 2.69; S: 5.97. The hydrogen content found in the elemental analysis was slightly outside the accepted range (6.09 vs 5.67%; Δ = 0.42). This could be attributed to the presence of small traces of Et_2_O in the sample (see the Supporting Information), which remained in spite of the compound being dried under vacuum. ESI-HRMS (*m*/*z*): exact mass calcd for C_23_H_30_NO_4_, 384.2169 [(M – CF_3_SO_3_)^+^]; found, 384.2174. IR (cm^–1^): ν(CO) 1740 (s), 1683 (s). ^1^H NMR (300.1 MHz): δ 0.57 (br d, 1 H, CH_2_, nor, ^2^*J*_HH_ = 12.6 Hz), 0.84 (d, 1 H, CH_2_, nor, ^2^*J*_HH_ = 12.3 Hz), 1.28 (s, 3 H, Me, CMe_2_), 1.29–1.55 (m, 4 H, CH_2_, nor), 1.56 (d, partially obscured by the resonance of CH_2_ of nor, 1 H, Hα or Hβ, ^3^*J*_HH_ = 7.2 Hz), 1.83 (s, 3 H, Me, CMe_2_), 2.01 (d, 1 H, Hα or Hβ, ^3^*J*_HH_ = 7.5 Hz), 2.62 (br s, 1 H, CH, nor), 2.75 (d, partially obscured by the resonance of CH of nor, 1 H, CH_2_Ar, ^2^*J*_HH_ = 16.8 Hz), 2.76 (br s, 1 H, CH, nor), 3.02 (d, 1 H, CH_2_Ar, ^2^*J*_HH_ = 17.1 Hz), 3.10 (s, 3 H, MeO), 3.90 (s, 3 H, MeO), 7.07 (m, 1 H, H5), 7.37 (m, 2 H, H7 + H6), 7.85 (m, 1 H, H8), 7.99 (br d, 1 H, NH_2_, ^2^*J*_HH_ = 11.4 Hz), 9.62 (br d, 1 H, NH_2_, ^2^*J*_HH_ = 11.4 Hz). ^13^C{^1^H} NMR (75.5 MHz): δ 22.4 (Me, CMe_2_), 27.3 (CHα or CHβ), 28.5 (Me, CMe_2_), 28.6 (CH_2_, nor), 29.0 (CH_2_, nor), 29.6 (CHα or CHβ), 30.1 (CH_2_, nor), 35.5 (CH, nor), 35.7 (*C*(CO_2_Me)CH), 37.6 (CH, nor), 40.0 (CH_2_Ar), 52.6 (MeO), 54.6 (MeO), 57.8 (*C*Me_2_), 69.7 (C1), 120.4 (q, CF_3_SO_3_, ^1^*J*_CF_ = 319.5 Hz), 126.2 (C8a), 126.7 (CH7), 128.5 (CH5), 129.7 (CH6), 130.9 (C4a), 131.1 (CH8), 165.3 (CO), 174.1 (CO).

### Synthesis of **9f**

TlOTf (84 mg, 0.237 mmol) was added to a solution of complex **7f** (125 mg, 0.237 mmol) in acetone (15 mL), and the mixture was stirred at room temperature for 12 h under an CO atmosphere, using a toy balloon. Decomposition to metallic palladium was observed. The suspension was filtered through a plug of Celite, the filtrate was concentrated to ca. 1 mL, and Et_2_O (20 mL) was added. The suspension was filtered, and the solid was washed with Et_2_O (2 × 2 mL) and air-dried to give the compound **9f** as a colorless solid. Yield: 103 mg, 0.177 mmol, 75%. Mp: 223 °C. Λ_M_ (Ω^–1^ mol^–1^ cm^2^): 101.4 (*c* = 5.04 × 10^–4^ M). Anal. Calcd for C_25_H_32_F_3_NO_9_S (579.592): C, 51.80; H, 5.56; N, 2.41, S; 5.53. Found: C, 51.56; H, 5.87; N, 2.50; S: 5.37. ESI-HRMS (*m*/*z*): exact mass calcd for C_24_H_32_NO_6_, 430.2230 [(M – CF_3_SO_3_)^+^]; found, 430.2225. IR (cm^–1^): ν(CO) 1771 vs, 1733 vs, 1626 s. ^1^H NMR (400.9 MHz, acetone-*d*_6_): δ 1.10 (m, 1 H, CH_2_, nor), 1.21 (m, 1 H, CH_2_, nor), 1.31 (m, 2 H, CH_2_, nor), 1.48 (s, 3 H, Me, CMe_2_), 1.54 (m, 2 H, CH_2_, nor), 1.58 (s, 3 H, Me, CMe_2_), 2.28 (br s, 1 H, CH, nor), 2.50 (br s, 1 H, CH, nor), 2.73 (d, 1 H, Hα, ^3^*J*_HH_ = 8.0 Hz), 2.88 (d, 1 H, Hβ, ^3^*J*_HH_ = 8.0 Hz), 3.10 (d, 1 H, CH_2_Ar, ^2^*J*_HH_ = 14.4 Hz), 3.51 (s, 3 H, MeO), 3.66 (s, 3 H, MeO), 3.69 (d, 1 H, CH_2_Ar, ^2^*J*_HH_ = 14.4 Hz), 4.99 (s, 1 H, H7), 7.26–7.33 (m, 2 H, H4 + H5), 7.37 (m, 1 H, H3), 7.63 (m, 1 H, H6), 7.70 (br s, 3 H, NH_3_). The resonance corresponding to the OH group was not observed. ^13^C{^1^H} NMR (100.8 MHz, acetone-*d*_6_): δ 25.3 (Me, CMe_2_), 26.9 (Me, CMe_2_), 27.6 (CH_2_, nor), 28.5 (CH_2_, nor), 35.1 (CH_2_, nor), 40.9 (CH, nor), 41.1 (CH, nor), 41.7 (CH_2_Ar), 51.1 (CHα), 51.3 (CHβ), 52.2 (MeO), 53.7 (MeO), 53.0 (CHAr), 57.4 (*C*Me_2_), 90.2 (C8), 128.2 (CH5), 128.7 (CH4), 131.4 (CH6), 133.1 (CH3), 134.5 (C1), 135.0 (C2), 166.3 (*C*O_2_Me), 171.2 (*C*O_2_Me), 177.6 (CO_2_H).

### Synthesis of **10c**

In a Carius tube, TlOTf (72 mg, 0.204 mmol) was added to a suspension of the complex **7c**·CHCl_3_ (100 mg, 0.161 mmol) in acetone (15 mL) and the mixture was stirred for 15 min. CO was bubbled through the suspension for 3 min, the pressure of CO was increased to 1 atm, the tube was sealed, and the mixture was stirred at room temperature for 20 h. Decomposition to metallic palladium was observed. The suspension was filtered through a plug of Celite, the filtrate was concentrated to ca. 1 mL, and Et_2_O (20 mL) was added. The suspension was filtered, the solvent was removed from the filtrate, and the solid residue was stirred in *n-*pentane (20 mL). The resulting suspension was filtered, and the solid was washed with *n-*pentane (2 × 5 mL) and air-dried to give the compound **10c** as a colorless solid. Yield: 84 mg, 0.152 mmol, 94%. Mp: 160 °C. Λ_M_ (Ω^–1^ mol^–1^ cm^2^): 117.1 (*c* = 5.00 × 10^–4^ M). Compound **10c** was hygroscopic, and no satisfactory elemental analysis could be obtained. ESI-HRMS (*m*/*z*): exact mass calcd for C_27_H_34_NO_2_, 404.2590 [(M – CF_3_SO_3_)^+^]; found, 404.2589. IR (cm^–1^): ν(NH) 3452 br; ν(CO) 1704 s. ^1^H NMR (400.9 MHz): δ 0.98 (br d, 1 H, CH_2_, nor, ^2^*J*_HH_ = 10.4 Hz), 1.07 (s, 3 H, Me, CMe_2_), 1.29 (m, 2 H, CH_2_, nor), 1.42 (m, partially obscured by the resonance of Me group, 1 H, CH_2_, nor), 1.45 (s, 3 H, Me, CMe_2_), 1.60 (m, 1 H, CH_2_, nor), 1.81 (br s, 1 H, CH, nor), 1.83 (br d, partially obscured by the resonance of CH of nor, 1 H, CH_2_, nor), 2.19 (s, 3 H, MeC=), 2.22 (d, 1 H, CH_2_Ar, ^2^*J*_HH_ = 13.6 Hz), 2.60 (br d, 1 H, CH, nor, ^3^*J*_HH_ = 4.0 Hz), 3.08 (d, 1 H, Hα, ^3^*J*_HH_ = 10.0 Hz), 3.22 (d, 1 H, Hβ, ^3^*J*_HH_ = 9.6 Hz), 3.52 (d, 1 H, CH_2_Ar, ^2^*J*_HH_ = 13.2 Hz), 6.58 (br s, 1 H), 6.77 (d, 1 H, H3, ^3^*J*_HH_ = 7.6 Hz), 6.90–6.99 (m, 4 H), 7.06–7.12 (m, 6 H), 7.33 (br s, 1 H, CO_2_H). ^13^C{^1^H} NMR (100.8 MHz): δ 22.9 (*Me*C=), 24.1 (Me, CMe_2_), 26.0 (Me, CMe_2_), 29.0 (CH_2_, nor), 30.9 (CH_2_, nor), 36.8 (CH_2_, nor), 39.7 (CH, nor), 40.3 (CH, nor), 41.5 (CH_2_Ar), 50.8 (CHβ), 54.1 (CHα), 57.3 (*C*Me_2_), 125.9 (CH4 or *p-*CH of Ph), 126.0 (CH4 or *p-*CH of Ph), 126.6 (CH5), 126.9 (br s, *o-*CH + *m*-CH, Ph), 129.1 (CH6), 129.4 (CH3), 131.3 (C2), 134.4 (C7), 140.1 (*i*-C, Ph), 140.3 (C8), 146.0 (C1), 177.6 (CO).

### Synthesis of **10d** and **9d**

In a Carius tube, TlOTf (50 mg, 0.141 mmol) was added to a solution of complex **7d·**1/2CHCl_3_ (75 mg, 0.124 mmol) in acetone (15 mL), and the mixture was stirred for 2 h. CO was bubbled through the suspension for 2 min, the pressure of CO was increased to 1 atm, the tube was sealed, and the mixture was stirred at room temperature for 15 h. Decomposition to metallic palladium was observed. The suspension was filtered through a plug of Celite, the solvent was removed from the filtrate, and Et_2_O (20 mL) was added. The mixture was filtered through a plug of Celite, the filtrate was concentrated to ca. 1 mL, and *n*-pentane (20 mL) was added. The resulting suspension was filtered, and the solid was washed with *n*-pentane (2 × 5 mL) and air-dried to give the compound **10d** as a colorless solid. Yield: 60 mg, 0.100 mmol, 81%. Mp: 122 °C. Λ_M_ (Ω^–1^ mol^–1^ cm^2^): 78.3 (*c* = 7.4 × 10^–4^ M). Compound **10d** was hygroscopic, and no satisfactory elemental analysis could be obtained. ESI-HRMS (*m*/*z*): exact mass calcd for C_28_H_34_NO_4_, 448.2482 [(M – CF_3_SO_3_)^+^]; found: 448.2484. IR (cm^–1^): ν(NH) 3486 br; ν(CO) 1715 vs, 1626 s. ^1^H NMR (300.1 MHz): δ 1.02 (br d, 1 H, CH_2_, nor, ^2^*J*_HH_ = 9.9 Hz), 1.1 (s, 3 H, Me, CMe_2_), 1.20–1.32 (m, 2 H, CH_2_, nor), 1.36–1.47 (m, 2 H, CH_2_, nor), 1.51 (s, 3 H, Me, CMe_2_), 1.79 (br d, 1 H, CH, nor, ^3^*J*_HH_ = 2.7 Hz), 1.83 (br d, 1 H, CH_2_, nor, ^2^*J*_HH_ = 9.9 Hz), 2.19 (d, 1 H, CH_2_Ar, ^2^*J*_HH_ = 14.1 Hz), 2.44 (br d, 1 H, CH, nor, ^3^*J*_HH_ = 3.6 Hz), 3.21 (d, 1 H, Hα, ^3^*J*_HH_ = 9.3 Hz), 3.45 (d, 1 H, CH_2_Ar, ^2^*J*_HH_ = 13.8 Hz), 3.57 (d, 1 H, Hβ, ^3^*J*_HH_ = 8.4 Hz), 3.79 (s, 3 H, OMe), 6.59 (br s, 1 H), 6.80 (d, 1 H, H3, ^3^*J*_HH_ = 7.8 Hz), 6.90–7.09 (m, 6 H), 7.11–7.20 (m, 3 H), 7.40 (br s, 1 H, CO_2_H). ^13^C{^1^H} NMR (75.5 MHz): δ 23.9 (Me, CMe_2_), 26.8 (Me, CMe_2_), 29.5 (CH_2_, nor), 30.3 (CH_2_, nor), 36.6 (CH_2_, nor), 39.7 (CH, nor), 41.3 (CH, nor), 42.1 (CH_2_Ar), 50.9 (CHβ), 52.6 (OMe), 55.2 (CHα), 56.8 (*C*Me_2_), 118.3 (C), 121.4 (C), 126.7 (CH), 126.9 (CH5), 128.2 (CH), 130.2 (C7 or C8), 131.1 (CH), 131.4 (CH3), 132.1 (C1 or C2), 138.3 (C1 or C2), 138.6 (C), 156.3 (C7 or C8), 169.5 (*C*O_2_Me), 177.7 (CO_2_H).

In a Carius tube, a solution of amino acid **10d** (45 mg, 0.075 mmol) in CHCl_3_ (2 mL) was heated at 65 °C for 7 days. The solvent was removed from the resulting white suspension to ca. 0.5 mL, and Et_2_O (10 mL) was added. The suspension was filtered, and the solid was washed with Et_2_O (2 × 3 mL) and air-dried to afford the cyclic lactone **9d** as a colorless solid. Yield: 30 mg, 0.048 mmol, 64%. Mp: 251 °C. Λ_M_ (Ω^–1^ mol^–1^ cm^2^): 88.8 (*c* = 4.1 × 10^–4^ M). Anal. Calcd for C_29_H_34_F_3_NO_7_S (597.643): C, 58.28; H, 5.73; N, 2.34, S; 5.37. Found: C, 58.15; H, 5.56; N, 2.12; S: 5.42. ESI-HRMS (*m*/*z*): exact mass calcd for C_28_H_34_NO_4_, 448.2482 [(M – CF_3_SO_3_)^+^]; found, 448.2493. IR (cm^–1^): ν(NH) 3167 m; ν(CO) 1749 s, 1733 s. ^1^H NMR (400.9 MHz, DMSO-*d*_6_): δ 0.69 (m, 2 H, CH_2_, nor), 0.91–0.94 (m, 2 H, 1 H of CH_2_ + 1 H of CH_2_, nor), 1.19 (s, 4 H, Me of CMe_2_ + CH of nor), 1.23 (s, 3 H, Me, CMe_2_), 1.26–1.28 (m, 2 H, 1 H of CH_2_ + 1 H of CH_2_, nor), 1.69 (s, 1 H, CH, nor), 2.17 (s, 1 H, CH, nor), 2.19 (s, 1 H, CH, nor), 2.88 (d, 1 H, CH_2_Ar, ^2^*J*_HH_ = 14.0 Hz), 3.21 (s, 3 H, MeO), 3.41 (d, 1 H, CH_2_Ar, ^2^*J*_HH_ = 13.6 Hz), 4.86 (s, 1 H, H7), 7.28 (br t, 1 H, H5, ^3^*J*_HH_ = 7.2 Hz), 7.32–7.50 (m, 6 H, H3 + H4 + CH of Ph), 7.51–7.60 (br s, 1 H, CH, Ph), 7.63 (d, 1 H, H6, ^3^*J*_HH_ = 8.0 H), 7.87 (br s, 3 H, NH_3_). The resonance corresponding to the OH group was not observed. ^13^C{^1^H} NMR (100.8 MHz, DMSO-*d*_6_): δ 24.8 (Me, CMe_2_), 24.9 (Me, CMe_2_), 27.4 (CH_2_, nor), 27.8 (CH_2_, nor), 33.5 (CH_2_, nor), 39.7 (CH, nor), 39.8 (CH, nor), 41.0 (CH_2_Ar), 49.5 (CH, nor), 50.4 (CH, nor), 51.5 (MeO), 54.7 (*C*Me_2_), 55.6 (CH7), 89.7 (C8), 127.2 (CH5), 127.6 (CH, Ph), 127.8 (br s, CH, Ph), 128.4 (CH4), 131.1 (CH6), 131.5 (C1), 132.2 (CH3), 135.1 (C2), 138.4 (*i*-C, Ph), 169.4 (*C*O_2_Me), 177.4 (CO_2_H).

### Single-Crystal X-ray Structure Determinations

Single crystals were obtained as follows: complex *anti*-**1a**, slow diffusion of *n*-pentane into a solution of a 2.5/1 mixture of *anti*/*syn*-**1a** in CH_2_Cl_2_; complex *anti*-**1b**, slow diffusion of Et_2_O into a solution of a 3/1 mixture of *anti*-/*syn*-**1b** in CHCl_3_; complex *syn*-**1b**, slow diffusion of *n*-pentane into a solution of a 1/3 mixture of *anti*-/*syn*-**1b** in CHCl_3_; complex *anti*-**2a**·CH_2_Cl_2_, slow diffusion of *n*-pentane into a solution of a 5/1 mixture of *anti*-/*syn*-**2a** in CH_2_Cl_2_; complex *syn*-**2a**, slow diffusion of *n*-pentane into a solution of the complex in CHCl_3_, complex *anti*-**2b**·CHCl_3_, slow diffusion of *n*-pentane into a solution of a 3/1 mixture of *anti*-/*syn*-**2b** in CHCl_3_; complex *syn*-**2b**: slow diffusion of *n*-pentane into a solution of a 1/3 mixture of *anti*-/*syn*-**2b** in CHCl_3_; complex *syn*-**3b**·CH_2_Cl_2_: ,slow diffusion of *n*-pentane into a solution of *anti*-/*syn*-**3b** in CH_2_Cl_2_; compound **5b·**H_2_O, slow diffusion of *n*-pentane into a solution of the complex in CH_2_Cl_2_, compounds **7c**·CHCl_3_, **7d**·1/2CHCl_3_, **7f**, and **8f**, slow diffusion of *n*-pentane into a solution of the corresponding compound in CHCl_3_; compound **9d**, slow diffusion of Et_2_O into a solution of the compound in acetone; compound **9f**·Et_2_O, slow diffusion of Et_2_O into a solution of **9f** in acetone; compound **10c**·H_2_O, slow diffusion of *n*-pentane into a solution of **10c** in CHCl_3_.

#### Data Collection

Crystals suitable for X-ray diffraction were mounted in inert oil on a glass fiber and transferred to a Bruker diffractometer. Data were recorded at 100(2) K, using graphite-monochromated Mo Kα radiation (λ = 0.71073 Å) and the ω-scan mode. Multiscan absorption corrections were applied for all complexes.

#### Structure Solution and Refinements

Crystal structures were solved by Patterson (*syn***-1b**, *anti***-2b**·CHCl_3_, and **7c**·CHCl_3_) or direct methods (*anti***-1a**, *anti***-1b**, *anti***-2a**·CH_2_Cl_2_, *syn***-2a**, *syn***-2b**, *syn*-**3b**·CH_2_Cl_2_, **6b**·H_2_O, **7d**·1/2CHCl_3_, **7f**, **8f**, **9d**, **9f**·Et_2_O, and **10c·**H_2_O), and all non-hydrogen atoms were refined anisotropically on *F*^2^ using the program SHELXL-2018/3.^42^ Hydrogen atoms were refined as follows: complex *anti***-1a**, NH_2_, free with SADI; ordered methyl, rigid group; all others, riding; compounds *anti***-1b**, *syn***-1b**, *anti***-2a**·CH_2_Cl_2_, *syn***-2a**, *syn***-2b**, **7c**·CHCl_3_, **7d**·1/2CHCl_3_, **7f**, **8f**, and **9d**, NH_2_ or NH_3_, free with SADI; methyl, rigid group; all others, riding; complex *anti*-**2b**·CHCl_3_, NH_2_, free with DFIX; methyl, rigid group; all others, riding; compounds *syn*-**3b**·CH_2_Cl_2_ and **9f**·Et_2_O, NH_2_ or NH_3_, free; methyl, rigid group; all others, riding; compound **6b**·H_2_O, NH_3_ and H_2_O, free with SADI; methyl, rigid group; all others, riding; compound **10c**·H_2_O, NH_3_, free with SADI; H_2_O (crystallization water), free with DFIX; methyl, rigid group; all others, riding.

#### Special Features

For *anti*-**1a**, one methyl group was disordered over two positions, with a ca. 95/5 occupancy distribution. For *syn***-1b**, one phenyl ring was disordered over two positions, with a ca. 57/43 occupancy distribution. For *syn*-**2a**, the CO_2_CH_2_CH_3_ fragment was disordered over two positions, with a ca. 75/25 occupancy distribution. The structure was refined as an inversion twin. Absolute structure (Flack) parameter:^43^ 0.027(8). For *anti*-**2b**·CHCl_3_, the chloroform molecule was disordered over two positions, with a ca. 81/19 occupancy distribution. For **7d**·1/2CHCl_3_, the crystallization solvent (half a molecule of chloroform), located on a crystallographic inversion center, was disordered over two positions, with a ca. 62/38 occupancy distribution.

Relevant crystallographic data, details of the refinements, and details (including symmetry operators) of hydrogen bonds for the compounds *anti***-1a**, *anti***-1b**, *syn***-1b**, *anti***-2a**·CH_2_Cl_2_, *syn***-2a**, *anti***-2b**·CHCl_3_, *syn***-2b**, *syn*-**3b**·CH_2_Cl_2_, **6b**·H_2_O, **7c**·CHCl_3_, **7d**·1/2CHCl_3_, **7f**, **8f**, **9d**, **9f**·Et_2_O, and **10c·**H_2_O are given in the Supporting Information.

## Supporting Information Available

For *anti*-**1a**, *anti*-1b, *syn*-**1b**, *anti*-**2a**·CH_2_Cl_2_, *syn*-**2a**, *anti*-**2b**·CHCl_3_, *syn*-**2b**, *syn*-**3b**·CH_2_Cl_2_, **6b**·H_2_O, **7c**·CHCl_3_, **7d**·1/2CHCl_3_, **7f**, **8f**, **9d**, **9f**·Et_2_O, and **10c**·H_2_O, relevant crystallographic data, details of the refinements, details of hydrogen bonds (including symmetry operators), and NMR spectra (PDF)

## Accession Codes

CCDC 2047166–2047181 contain the supplementary crystallographic data for this paper. These data can be obtained free of charge via www.ccdc.cam.ac.uk/data_request/cif, or by emailing data_request@ccdc.cam.ac.uk, or by contacting The Cambridge Crystallographic Data Centre, 12 Union Road, Cambridge CB2 1EZ, UK; fax: +44 1223 336033.
