# Supplementary material for: Sequential Insertion of Alkynes, Alkenes, and CO into the Pd–C Bond of ortho-Palladated Primary Phenethylamines: from η3-Allyl Complexes and Enlarged Palladacycles to Functionalized Arylalkylamines
Source: Organometallics. 2021 Jan 29;40(4):539–56. doi: 10.1021/acs.organomet.0c00787 (PMC8895685; doi:10.1021/acs.organomet.0c00787)
Supplement: Supplementary file 1 — om0c00787_si_001.pdf [file om0c00787_si_001.pdf]

SUPPORTING INFORMATION FOR

Sequential Insertion of Alkynes, Alkenes and CO into the Pd–C Bond of *ortho*-Palladated Primary Phenethylamines: from η^3-Allyl Complexes and Enlarged Palladacycles to Functionalized Arylalkylamines

José-Antonio García-López,*[a] María-José Oliva-Madrid,[a] Delia Bautista,[b] José Vicente,[a] and Isabel Saura-Llamas*[a]

[a]Grupo de Química Organometálica, Departamento de Química Inorgánica, Facultad de Química, Universidad de Murcia, E–30100 Murcia, Spain. [b]ACTI, Universidad de Murcia, E–30100 Murcia, Spain

Table S1. Crystal data and structure refinement details for complexes
anti-**1a**, *anti*-**1b**, *syn*-**1b**, and *anti*-**2a**·CH_2Cl_2 .... S5
Table S2. Crystal data and structure refinement details for complexes
syn-**2a**, *anti*-**2b**·$CHCl_3$, *syn*-**2b**, and **3b**·CH_2Cl_2 .... S6
Table S3. Crystal data and structure refinement details for compounds
6b·H_2O, **7c**·$CHCl_3$, **7d**·1/2$CHCl_3$, and **7f**........ S7
Table S4. Crystal data and structure refinement details for compounds
8f, **9d**, **9f**·Et_2O, and **10c**·H_2O S8
Figure S1. X-ray thermal ellipsoid plot of complex *anti*-**1b** S9
Figure S2. X-ray thermal ellipsoid plot of complex *syn*-**1b** S10
Figure S3. X-ray thermal ellipsoid plot of complex *anti*-**2a**·CH_2Cl_2 .... S11
Figure S4. X-ray thermal ellipsoid plot of complex *syn*-**2a** S12
Figure S5. X-ray thermal ellipsoid plot of complex *anti*-**2b**·$CHCl_3$........ S13
Figure S6. X-ray thermal ellipsoid plot of one (A') of the two independent molecules
of complex *syn*-**3b**·CH_2Cl_2 .... S14
Figure S7. X-ray thermal ellipsoid plot of complex **7d**·1/2$CHCl_3$........ S15
Figure S8. X-ray thermal ellipsoid plot of complex **7f**........ S16
Figure S9. X-ray thermal ellipsoid plot of the cation of compound **9d**........ S17
Hydrogen bonds data for compounds
anti-**1b** (Table S5 and Figure S10) S18
syn-**1b** (Table S6 and Figure S11) S19
anti-**2a**·CH_2Cl_2 (Table S7 and Figure S12) S20
syn-**2a** (Table S8 and Figure S13) S21
anti-**2b**·$CHCl_3$ (Table S9 and Figure S14)........ S22
syn-**2b** (Table S10 and Figure S15) S23
syn-**3b**·CH_2Cl_2 (Table S11 and Figures S16 and S17)........ S24
6b·H_2O (Table S12 and Figure S18)........ S25
7c·$CHCl_3$ (Table S13 and Figure S19)........ S26
7d·1/2$CHCl_3$ (Table S14 and Figure S20) S27
7f (Table S15 and Figure S21)........ S28
8f (Table S16 and Figure S22)........ S29
9d (Table S17 and Figure S23)........ S30
9f·Et_2O (Table S18 and Figure S24)........ S31
10c·H_2O (Table S19 and Figure S25) S32

^{1}H and $^{13}C\{^{1}H\}$ APT NMR spectra of compounds

anti/syn-**1a** (Figures S26 and S27) S33

syn-**1a** (Figures S28 and S29) S35

anti-**1b** (Figures S30 and S31) S37

syn-**1b** (Figures S32 and S33) S39

anti-**2a** (Figures S34 and S35) S41

syn-**2a** (Figures S36 and S37) S43

anti-**2b** (Figures S38 and S39) S45

syn-**2b** (Figures S40 and S41) S47

anti/syn-**3b** (Figures S42, S43, S44 and S45) S49

4a·$1/2H_2O$ (Figures S46 and S47) S53

4b (crude) (Figure S48) S55

5b·H_2O (Figures S49 and S50) S56

6d (Figures S51 and S52) S58

7c·$CHCl_3$ (Figures S53 and S54) S60

7d·$1/2CHCl_3$ (Figures S55 and S56) S62

7f (Figures S57 and S58) S64

8f (Figures S59 and S60) S66

9d (Figures S61 and S62) S68

9f (Figures S63 and S64) S70

10c (Figures S65 and S66) S72

10d (Figures S67 and S68) S74

Table S1. Crystal Data and Structure Refinement Details for Complexes *anti*-**1a**, *anti*-**1b**, *syn*-**1b**, and *anti*-**2a**·CH_2Cl_2.

	anti-**1a**	*anti*-**1b**	*syn*-**1b**	*anti*-**2a**·CH_2Cl_2
formula	$C_{32}H_{32}BrNO_2Pd$	$C_{32}H_{32}ClNPd$	$C_{32}H_{32}ClNPd$	$C_{30}H_{34}BrCl_2NO_4Pd$
fw	648.90	572.43	572.43	729.79
temp (K)	100(2)	100(2)	100(2)	100(2)
cryst habit	yellow block	colorless block	colorless prism	colorless prism
cryst size (mm)	0.20 x 0.18 x 0.10	0.38 x 0.26 x 0.17	0.26 x 0.19 x 0.07	0.230 x 0.080 x 0.030
cryst syst	triclinic	monoclinic	triclinic	monoclinic
space group	$P\bar{1}$	$P2_1/c$	$P\bar{1}$	$P2_1/n$
a (Å)	8.9631(8)	10.2233(9)	10.6754(9)	16.9537(8)
b (Å)	11.7417(11)	17.5358(16)	11.0500(11)	8.8151(4)
c (Å)	14.2801(12)	15.6984(14)	14.3048(11)	20.9006(9)
α (deg)	110.003(2)	90	70.041(2)	90
β (deg)	104.351(2)	106.430(2)	68.606(3)	107.9949(16)
γ (deg)	92.262(2)	90	61.474(2)	90
V (Å^3)	1355.3(2)	2699.4(4)	1351.3(2)	2970.8(2)
Z	2	4	2	4
ρ_{calcd} (Mg m^{-3})	1.590	1.409	1.407	1.632
μ(Mo, Kα) (mm^{-1})	2.189	0.807	0.806	2.186
F(000)	656	1176	588	1472
θ range (deg)	1.86–28.73	1.78–28.72	1.56–28.72	2.05–28.80
no. rflns collected	16 682	32 642	16 608	96 802
no. indep rflns	6365	6573	6351	7710
R_{int}	0.0173	0.0305	0.0170	0.0705
max, min transmsn	0.811, 0.674	0.875, 0.764	0.946, 0.786	0.937, 0.633
no. of restraints/params	2/347	1/326	5/375	1/355
goodness of fit on F^2	1.045	1.101	1.100	1.054
R1 ($I > 2\sigma(I)$)	0.0207	0.0346	0.0234	0.0370
wR2 (all rflns)	0.0536	0.0758	0.0578	0.0800
largest diff peak, hole (e·Å^{-3})	0.481, –0.579	0.665, –0.435	0.511, –0.611	1.315, –1.403

Table S2. Crystal Data and Structure Refinement Details for Complexes *syn*-**2a**, *anti*-**2b**·$CHCl_3$, *syn*-**2b**, and *syn*-**3b**·CH_2Cl_2.

	syn-**2a**	*anti*-**2b**·$CHCl_3$	*syn*-**2b**	*syn*-**3b**·CH_2Cl_2
formula	$C_{29}H_{32}BrNO_4Pd$	$C_{30}H_{33}Cl_4NO_2Pd$	$C_{29}H_{32}ClNO_2Pd$	$C_{41}H_{43}Cl_2F_3N_2O_3PdS$
fw	644.86	687.77	568.40	878.13
temp (K)	100(2)	100(2)	100(2)	100(2)
cryst habit	yellow lath	colorless prism	yellow prism	colorless block
cryst size (mm)	0.21 x 0.16 x 0.016	0.13 x 0.09 x 0.08	0.20 x 0.09 x 0.08	0.37 x 0.12 x 0.09
cryst syst	orthorhombic	triclinic	triclinic	triclinic
space group	$P2_12_12_1$	$P\bar{1}$	$P\bar{1}$	$P\bar{1}$
a (Å)	8.9693(5)	10.6530(8)	9.0931(6)	11.4056(8)
b (Å)	9.1468(5)	12.4688(9)	11.9585(8)	18.0651(13)
c (Å)	32.5761(18)	13.3325(11)	12.0592(8)	20.4085(15)
α (deg)	90	116.035(2)	80.987(2)	76.531(2)
β (deg)	90	93.499(3)	83.351(3)	83.006(3)
γ (deg)	90	103.312(2)	85.948(2)	80.422(2)
V (Å^3)	2672.6(3)	1521.7(2)	1284.57(15)	4017.2(5)
Z	4	2	2	4
ρ_{calcd} (Mg m^{-3})	1.603	1.501	1.470	1.452
μ(Mo, Kα) (mm^{-1})	2.225	0.989	0.853	0.701
F(000)	1304	700	584	1800
θ range (deg)	2.31–28.37	1.73–28.68	1.72–28.59	1.72–28.74
no. rflns collected	65 022	18 867	15 851	49 595
no. indep rflns	6654	7173	6030	18 882
R_{int}	0.0729	0.0266	0.0211	0.0260
max, min transmsn	0.862, 0.738	0.925, 0.815	0.935, 0.786	0.940, 0.850
no. of restraints/params	9/359	103/383	1/318	0/993
goodness of fit on F^2	1.032	0.937	1.091	1.204
R1 ($I > 2\sigma(I)$)	0.0309	0.0387	0.0286	0.0418
wR2 (all rflns)	0.0531	0.0883	0.0666	0.0880
largest diff peak, hole (e·Å^{-3})	0.496, –0.616	0.803, –0.785	1.061, –0.525	0.755, –0.628

Table S3. Crystal Data and Structure Refinement Details for Compounds **6b**·H_2O, **7c**·$CHCl_3$, **7d**·1/2$CHCl_3$, and **7f**.

	**6b**·H_2O	**7c**·$CHCl_3$	**7d**·1/2$CHCl_3$	**7f**
formula	$C_{33}H_{34}F_3NO_4S$	$C_{27}H_{33}Cl_4NPd$	$C_{27.5}H_{32.5}Cl_{2.5}NO_2Pd$	$C_{23}H_{30}ClNO_4Pd$
fw	597.67	619.74	604.07	526.33
temp (K)	100(2)	100(2)	100(2)	100(2)
cryst habit	pale yellow prism	colorless prism	colorless prism	yellow prism
cryst size (mm)	0.24 x 0.16 x 0.06	0.25 x 0.11 x 0.05	0.16 x 0.10 x 0.02	0.25 x 0.07 x 0.07
cryst syst	monoclinic	monoclinic	monoclinic	monoclinic
space group	$P2_1/c$	$P2_1/n$	$C2/c$	$P2_1/n$
a (Å)	22.3424(17)	11.4685(9)	23.7231(19)	12.0005(11)
b (Å)	10.7595(8)	10.8204(9)	14.2275(12)	13.1557(13)
c (Å)	12.6718(11)	21.5690(17)	17.3927(14)	14.2244(14)
α (deg)	90	90	90	90
β (deg)	91.435(2)	99.6340(10)	118.388(2)	105.146(2)
γ (deg)	90	90	90	90
V (Å^3)	3045.3(4)	2638.8(4)	5164.5(7)	2167.7(4)
Z	4	4	8	4
ρ_{calcd} (Mg m^{-3})	1.304	1.560	1.554	1.613
μ(Mo, Kα) (mm^{-1})	0.162	1.125	1.003	1.010
F(000)	1256	1264	2472	1080
θ range (deg)	1.82–28.76	1.89–28.70	1.73–27.88	1.98–28.67
no. rflns collected	36 514	31 637	30 962	26 555
no. indep rflns	7451	6410	6116	5278
R_{int}	0.0347	0.0293	0.0478	0.0306
max, min transmsn	0.990, 0.904	0.946, 0.837	0.980, 0.847	0.933, 0.831
no. of restraints/params	2/401	0/309	316/333	1/283
goodness of fit on F^2	1.187	1.135	1.107	1.061
R1 ($I > 2\sigma(I)$)	0.0627	0.0319	0.0435	0.0263
wR2 (all rflns)	0.1380	0.0692	0.0939	0.0614
largest diff peak, hole (e·Å^{-3})	0.627, –0.321	0.750, –0.507	1.144, –0.860	0.659, –0.322

Table S4. Crystal Data and Structure Refinement Details for Compounds **8f**, **9d**, **9f**·Et_2O, and **10c**·H_2O.

	**8f**	**9d**	**9f**·Et_2O	**10c**·H_2O
formula	$C_{24}H_{30}F_3NO_7S$	$C_{29}H_{34}F_3NO_7S$	$C_{29}H_{42}F_3NO_{10}S$	$C_{28}H_{36}F_3NO_6S$
fw	533.55	597.63	653.69	571.64
temp (K)	100(2)	100(2)	100(2)	100(2)
cryst habit	colorless prism	colorless needle	colorless lath	colorless prism
cryst size (mm)	0.23 x 0.14 x 0.11	0.13 x 0.03 x 0.03	0.20 x 0.15 x 0.03	0.09 x 0.07 x 0.014
cryst syst	triclinic	orthorhombic	monoclinic	triclinic
space group	$P\bar{1}$	*Pbca*	$P2_1/n$	$P\bar{1}$
a (Å)	9.4903(7)	18.0389(9)	8.7895(6)	8.9366(8)
b (Å)	9.9546(7)	15.3736(6)	25.4271(17)	12.0889(11)
c (Å)	13.3238(13)	19.6769(9)	13.8814(9)	13.8916(12)
α (deg)	105.493(2)	90	90	83.450(3)°
β (deg)	92.836(3)	90	96.550(2)	88.543(3)°
γ (deg)	96.262(2)	90	90	68.801(3)°
V (Å^3)	1201.68(17)	5456.9(4)	3082.1(4)	1389.9(2)
Z	2	8	4	2
ρ_{calcd} (Mg m^{-3})	1.475	1.455	1.409	1.366
μ(Mo, Kα) (mm^{-1})	0.204	0.189	0.181	0.179
F(000)	560	2512	1384	604
θ range (deg)	2.14–28.67	2.02–26.41	2.18–28.79	2.21–26.44
no. rflns collected	14 821	58 213	63 048	36 550
no. indep rflns	5647	5590	7999	5717
R_{int}	0.0184	0.1128	0.0584	0.0546
max, min transmsn	0.978, 0.849	0.862, 0.806	0.862, 0.799	0.862, 0.821
no. of restraints/params	1/337	3/385	0/415	6/376
goodness of fit on F^2	1.078	1.068	1.058	1.025
R1 ($I > 2\sigma(I)$)	0.0404	0.0496	0.0456	0.0437
wR2 (all rflns)	0.1012	0.1115	0.1034	0.1073
largest diff peak, hole (e·Å^{-3})	0.422, –0.430	0.745, –0.505	0.431, –0.399	0.517, –0.418

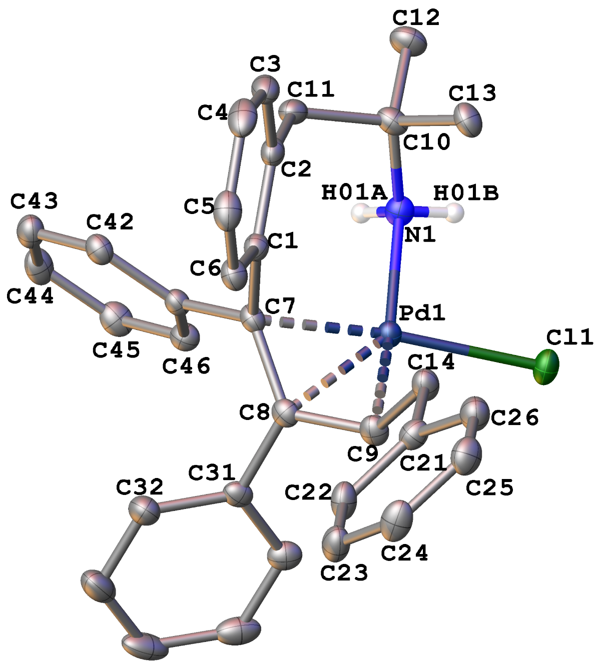

Figure S1. Thermal ellipsoid plot (50 % probability) of *anti*-**1b** along with the labeling scheme. The hydrogen atoms bonded to carbon have been omitted for clarity. Selected bond lengths (Å) and angles (deg): Pd(1)–Cl(1) = 2.3644(6), Pd(1)–N(1) = 2.1086(19), Pd(1)–C(7) = 2.118(2), Pd(1)–C(8) = 2.119(2), Pd(1)–C(9) = 2.114(2), C(7)–C(8) = 1.452(3), C(8)–C(9) = 1.430(3); Cl(1)–Pd(1)–N(1) = 88.46(6), C(7)–C(8)–C(9) = 121.24(19), C(8)–C(9)–C(14) = 132.3(2).

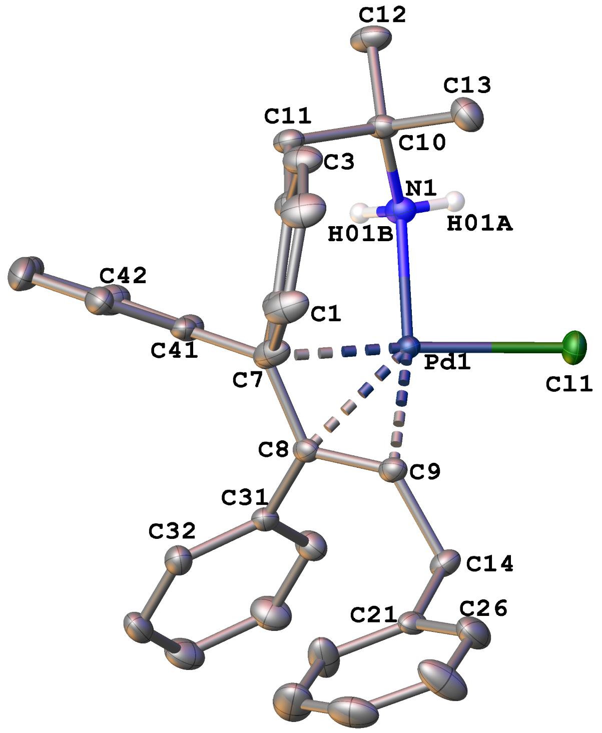

Figure S2. Thermal ellipsoid plot (50 % probability) of *syn*-**1b** along with the labeling scheme. The hydrogen atoms bonded to carbon have been omitted for clarity. Selected bond lengths (Å) and angles (deg): Pd(1)–Cl(1) = 2.3670(5), Pd(1)–N(1) = 2.1231(14), Pd(1)–C(7) = 2.1306(15), Pd(1)–C(8) = 2.1232(15), Pd(1)–C(9) = 2.1290(15), C(7)–C(8) = 1.445(2), C(8)–C(9) = 1.419(2); Cl(1)–Pd(1)–N(1) = 89.70(4), C(7)–C(8)–C(9) = 117.90(14), C(8)–C(9)–C(14) = 124.64(14).

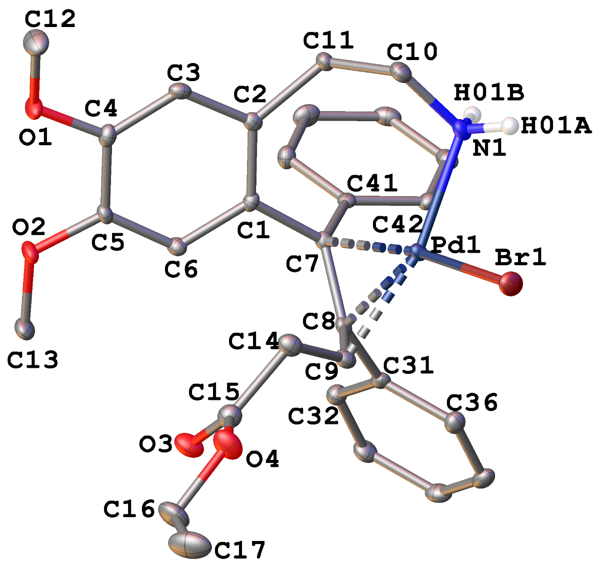

Figure S3. Thermal ellipsoid plot (50 % probability) of *anti*-**2a**·CH_2Cl_2 along with the labeling scheme. The solvent molecule and the hydrogen atoms bonded to carbon have been omitted for clarity. Selected bond lengths (Å) and angles (deg): Pd(1)–Br(1) = 2.4850(3), Pd(1)–N(1) = 2.105(2), Pd(1)–C(7) = 2.132(2), Pd(1)–C(8) = 2.108(3), Pd(1)–C(9) = 2.101(3), C(7)–C(8) = 1.447(4), C(8)–C(9) = 1.430(4); Br(1)–Pd(1)–N(1) = 91.03(6), C(7)–C(8)–C(9) = 120.4(2), C(8)–C(9)–C(14) = 132.1(3).

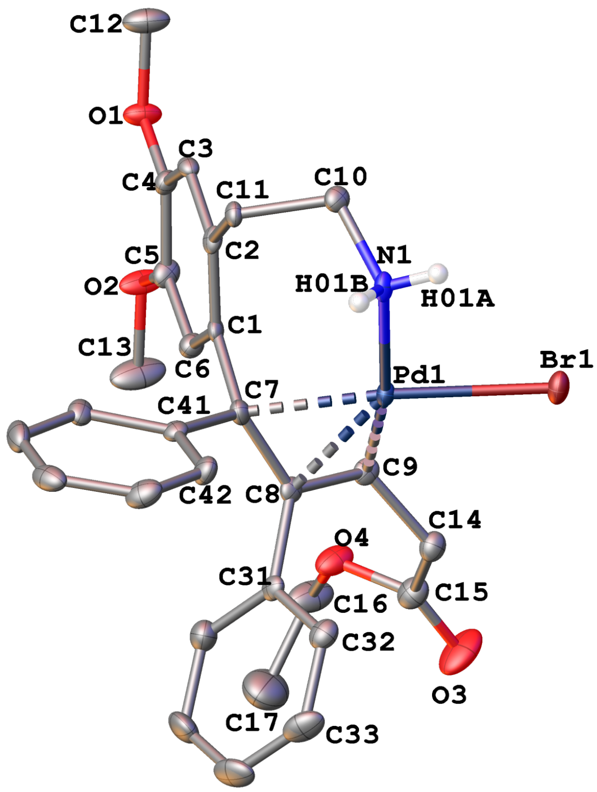

Figure S4. Thermal ellipsoid plot (50 % probability) of *syn*-**2a** along with the labeling scheme. The hydrogen atoms bonded to carbon have been omitted for clarity. Selected bond lengths (Å) and angles (deg): Pd(1)–Br(1) = 2.4878(5), Pd(1)–N(1) = 2.126(3), Pd(1)–C(7) = 2.132(4), Pd(1)–C(8) = 2.125(4), Pd(1)–C(9) = 2.115(3), C(7)–C(8) = 1.435(5), C(8)–C(9) = 1.418(5); Br(1)–Pd(1)–N(1) = 90.86(9), C(7)–C(8)–C(9) = 118.5(3), C(8)–C(9)–C(14) = 122.5(4).

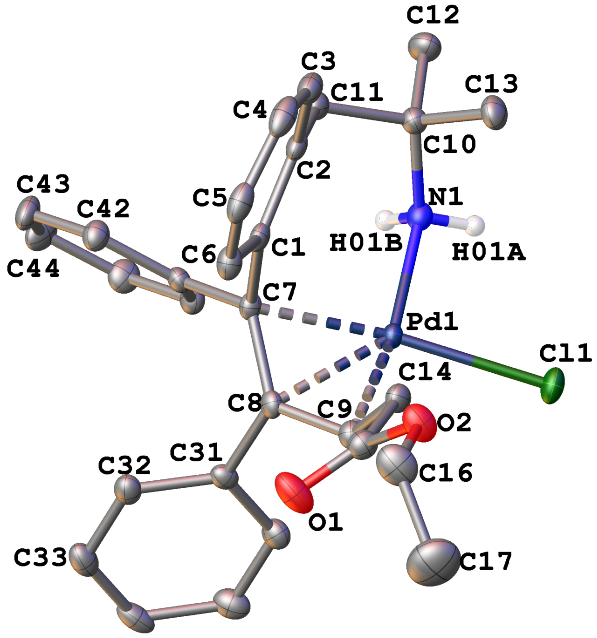

Figure S5. Thermal ellipsoid plot (50 % probability) of *anti*-**2b**·$CHCl_3$ along with the labeling scheme. The solvent molecule and the hydrogen atoms bonded to carbon have been omitted for clarity. Selected bond lengths (Å) and angles (deg): Pd(1)–Cl(1) = 2.3861(7), Pd(1)–N(1) = 2.119(2), Pd(1)–C(7) = 2.138(3), Pd(1)–C(8) = 2.114(3), Pd(1)–C(9) = 2.123(3), C(7)–C(8) = 1.439(4), C(8)–C(9) = 1.425(4); Cl(1)–Pd(1)–N(1) = 91.82(7), C(7)–C(8)–C(9) = 122.1(2), C(8)–C(9)–C(14) = 132.5(2).

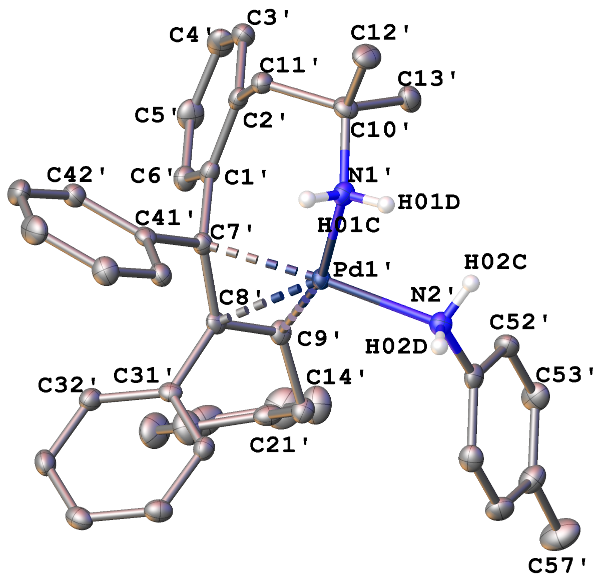

Figure S6. Thermal ellipsoid plot (50 % probability) of the cation of one (A') of the two independent molecules of complex *syn*-**3b**·CH_2Cl_2 along with the labeling scheme. The solvent molecule and the hydrogen atoms bonded to carbon have been omitted for clarity. Selected bond lengths (Å) and angles (deg): Pd(1')–N(1') = 2.130(2), Pd(1')–N(2') = 2.163(2), Pd(1')–C(7') = 2.117(2), Pd(1')–C(8') = 2.134(2), Pd(1')–C(9') = 2.165(2), C(7')–C(8') = 1.452(3), C(8')–C(9') = 1.413(3); N(1')–Pd(1')–N(2') = 88.94(8), C(7')–C(8')–C(9') = 117.5(2), C(8')–C(9')–C(14') = 124.0(2).

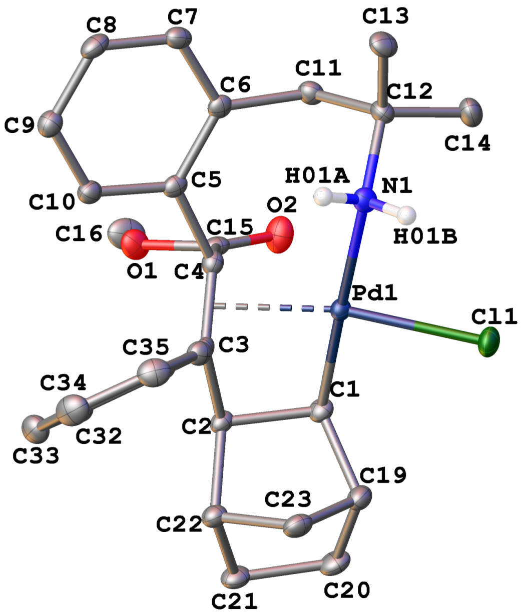

Figure S7. Thermal ellipsoid plot (50 % probability) of **7d**·1/2CH_2Cl_2 along with the labeling scheme. The solvent molecule and the hydrogen atoms bonded to carbon have been omitted for clarity. Selected bond lengths (Å) and angles (deg): Pd(1)–N(1) = 2.219(3), Pd(1)–Cl(1) = 2.3439(8), Pd(1)–C(1) = 2.041(3), Pd(1)–C(3) = 2.177(3), Pd(1)–C(4) = 2.170(3), Pd(1)–X = 2.058, C(1)–C(2) = 1.544(4), C(2)–C(3) = 1.534(4), C(3)–C(4) = 1.400(4), C(4)–C(5) = 1.511(4); N(1)–Pd(1)–Cl(1) = 86.31(7), Cl(1)–Pd(1)–C(1) = 92.68(9), C(1)–Pd(1)–X = 76.7, X–Pd(1)–N(1) = 104.3, C(2)–C(3)–C(4) = 118.3(3), C(3)–C(4)–C(5) = 123.3(3). X represents the midpoint of the double bond C(3)–C(4).

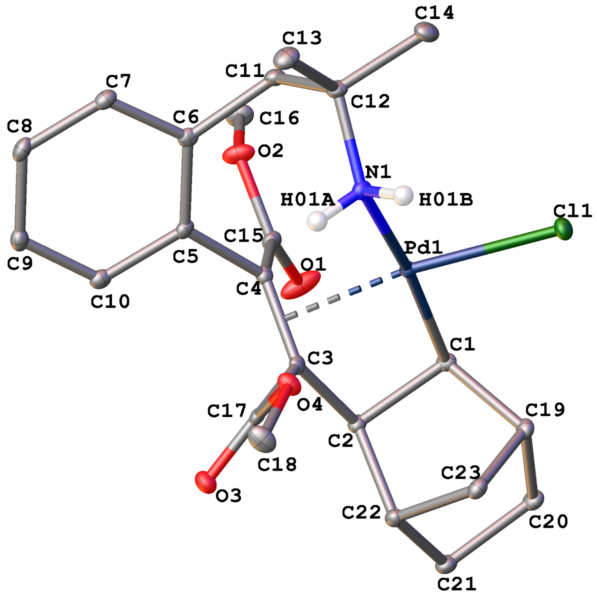

Figure S8. Thermal ellipsoid plot (30 % probability) of **7f** along with the labeling scheme. The hydrogen atoms bonded to carbon have been omitted for clarity. Selected bond lengths (Å) and angles (deg): Pd(1)–N(1) = 2.2240(16), Pd(1)–Cl(1) = 2.3363(5), Pd(1)–C(1) = 2.0583(18), Pd(1)–C(3) = 2.1355(18), Pd(1)–C(4) = 2.2042(18), Pd(1)–X = 2.053, C(1)–C(2) = 1.547(3), C(2)–C(3) = 1.534(2), C(3)–C(4) = 1.405(3), C(4)–C(5) = 1.515(3); N(1)–Pd(1)–Cl(1) = 89.49(5), Cl(1)–Pd(1)–C(1) = 92.99(5), C(1)–Pd(1)–X = 77.6, X–Pd(1)–N(1) = 99.7, C(2)–C(3)–C(4) = 121.82(16), C(3)–C(4)–C(5) = 121.20(17). X represents the midpoint of the double bond C(3)–C(4).

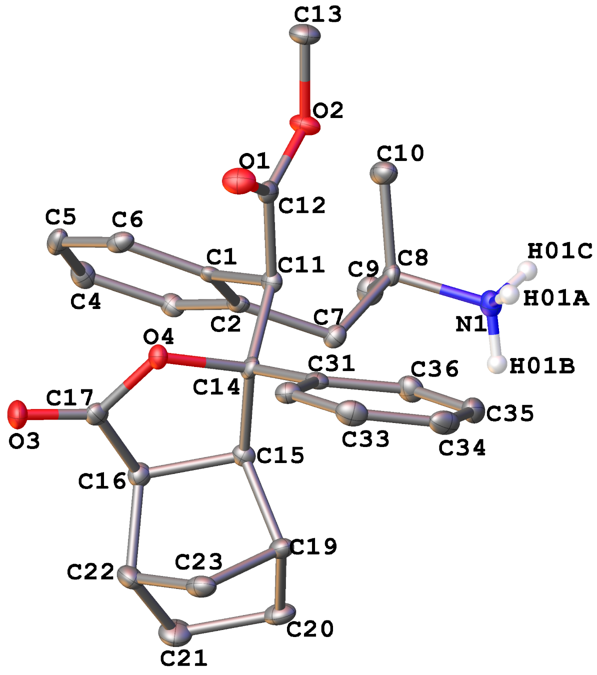

Figure S9. Thermal ellipsoid plot (50 % probability) of the cation of **9d** along with the labeling scheme. The hydrogen atoms bonded to carbon have been omitted for clarity. Selected bond lengths (Å) and angles (deg): N(1)–C(8) = 1.517(3), C(11)–C(12) = 1.523(3), C(11)–C(14) = 1.554(3), C(14)–O(4) = 1.461(3), C(17)–O(3) = 1.213(3), C(17)–O(4) = 1.338(3), C(16)–C(17) = 1.505(3); C(1)–C(11)–C(12) = 110.01(19), C(11)–C(14)–O(4) = 108.07(18), C(14)–O(4)–C(17) = 112.77(17), O(4)–C(17)–C(16) = 111.8(2), C(17)–C(16)–C(15) = 104.35(19), C(16)–C(15)–C(14) = 104.38(18), C(15)–C(14)–O(4) = 105.06(17).

Table S5. Hydrogen bonds for complex *anti*-**1b** (Å and deg).

D–H···A	d(D–H)	d(H···A)	d(D···A)	<(DHA)
N(1)–H(01A)···Cl(1)#1	0.85(2)	2.57(2)	3.339(2)	151(3)

Symmetry transformations used to generate equivalent atoms: #1 -x+1,-y+2,-z+1

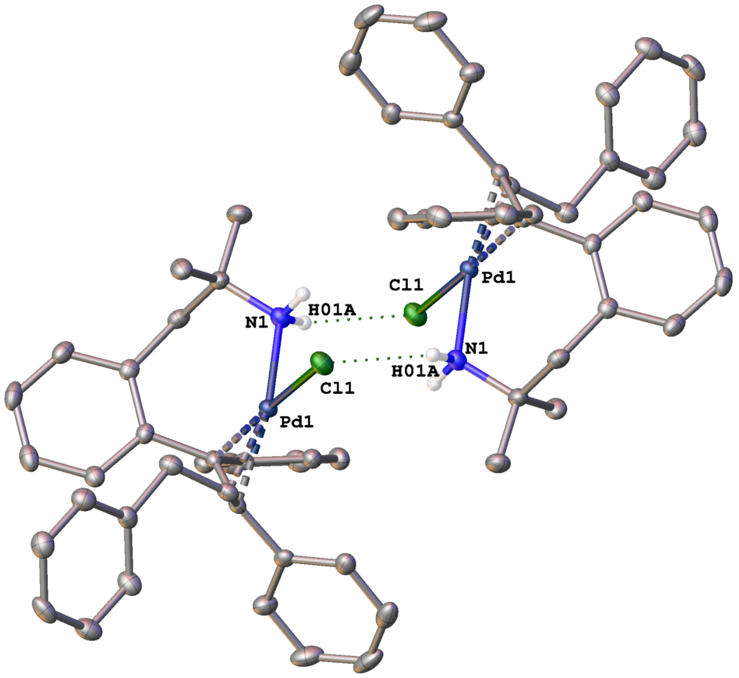

Figure S10. Thermal ellipsoid plot (50 % probability) of complex *anti*-**1b** showing the dimers formed through hydrogen bonds.

Table S6. Hydrogen bonds for complex *syn*-**1b** (Å and deg).

D–H···A	d(D–H)	d(H···A)	d(D···A)	<(DHA)
N(1)–H(01B)···Cl(1)#1	0.845(17)	2.71(2)	3.3336(15)	131.3(18)

Symmetry transformations used to generate equivalent atoms: #1 -x,-y+2,-z+1

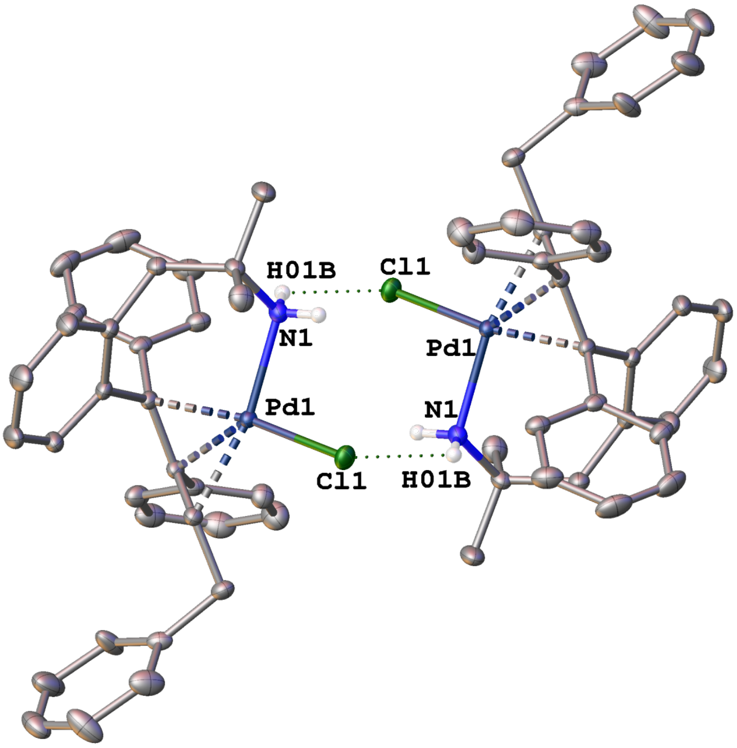

Figure S11. Thermal ellipsoid plot (50 % probability) of complex *syn*-**1b** showing the dimers formed through hydrogen bonds.

Table S7. Hydrogen bonds for complex *anti*-**2a**·CH_2Cl_2 (Å and deg).

D–H...A	d(D–H)	d(H···A)	d(D···A)	<(DHA)
C(99)–H(99B)···Br(1)#1	0.99	2.79	3.725(4)	157.6
C(99)–H(99A)···O(4)#1	0.99	2.53	3.343(4)	139.4
C(3)–H(3)···O(3)#2	0.95	2.44	3.344(3)	160.0
N(1)–H(01A)···Br(1)#1	0.91	2.72	3.472(2)	140.2

Symmetry transformations used to generate equivalent atoms:

#1 -x+3/2,y+1/2,-z+1/2   #2 x,y+1,z

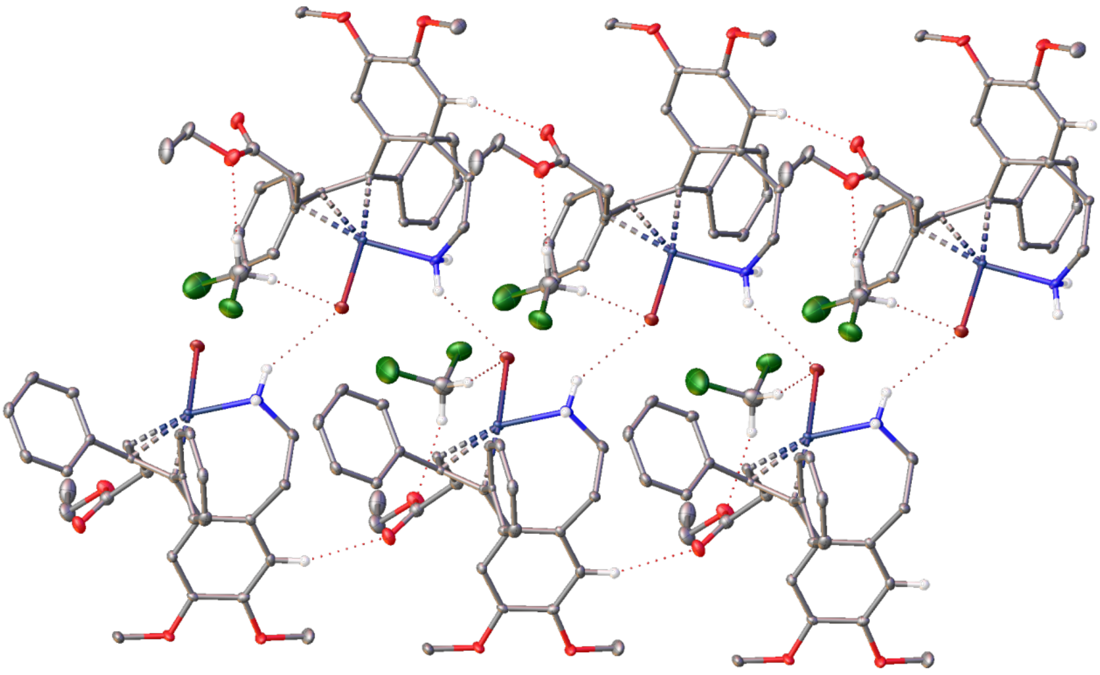

Figure S12. X-ray packing view (50 % probability) of *anti*-**2a**·CH_2Cl_2 showing the double chains parallel to the *b* axis formed through hydrogen bonds.

Table S8. Hydrogen bonds for complex *syn*-**2a** (Å and deg).

D–H···A	d(D–H)	d(H···A)	d(D···A)	<(DHA)
N(1)–H(01A)···Br(1)	0.82(3)	2.93(4)	3.297(3)	110(3)
N(1)–H(01B)···Br(1)#1	0.81(3)	2.71(3)	3.481(4)	160(4)

Symmetry transformations used to generate equivalent atoms: #1 x-1/2,-y+3/2,-z+2

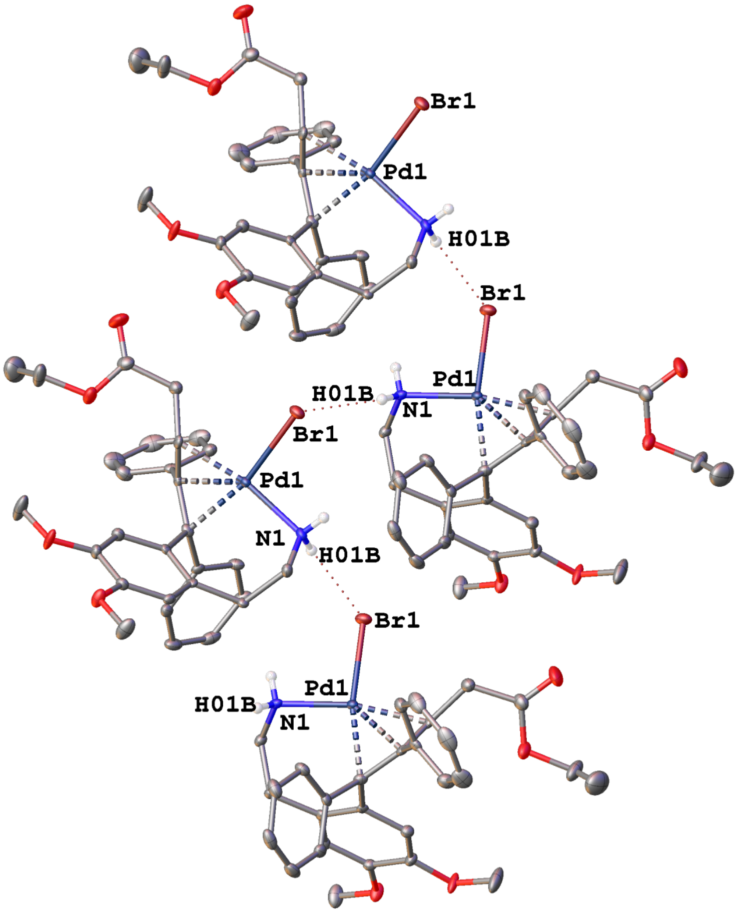

Figure S13. X-ray packing view (50 % probability) of *syn*-**2a** showing the *zigzag* chains parallel to the *a* axis formed through hydrogen bonds.

Table S9. Hydrogen bonds for complex *anti*-**2b**·$CHCl_3$ (Å and deg).

D–H···A	d(D–H)	d(H···A)	d(D···A)	<(DHA)
N(1)–H(01B)···Cl(1)#1	0.837(18)	2.69(2)	3.402(2)	144(3)
C(34)–H(34)···O(1)#2	0.95	2.42	3.350(4)	168.1

Symmetry transformations used to generate equivalent atoms:

#1 -x+1,-y+1,-z+1 #2 -x+1,-y+1,-z+2

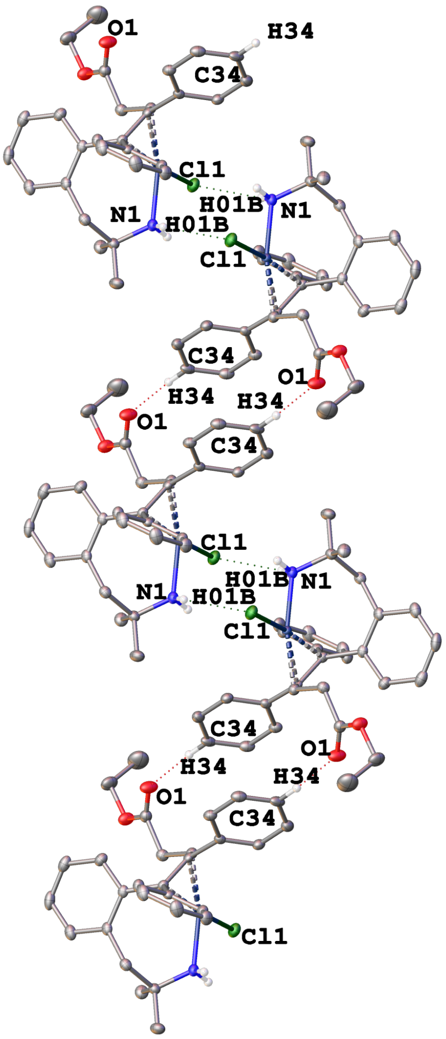

Figure S14. X-ray packing view (50 % probability) of *anti*-**2b**·$CHCl_3$ showing the *zigzag* chains parallel to the *c* axis formed through hydrogen bonds.

Table S10. Hydrogen bonds for complex *syn*-**2b** (Å and deg).

D–H...A	d(D–H)	d(H···A)	d(D···A)	<(DHA)
N(1)–H(01A)···Cl(1)#1	0.844(19)	2.61(2)	3.3340(19)	145(2)
C(42)–H(42)···O(2)#2	0.95	2.57	3.462(3)	155.9

Symmetry transformations used to generate equivalent atoms: #1 -x+2,-y,-z+1 #2 x-1,y,z

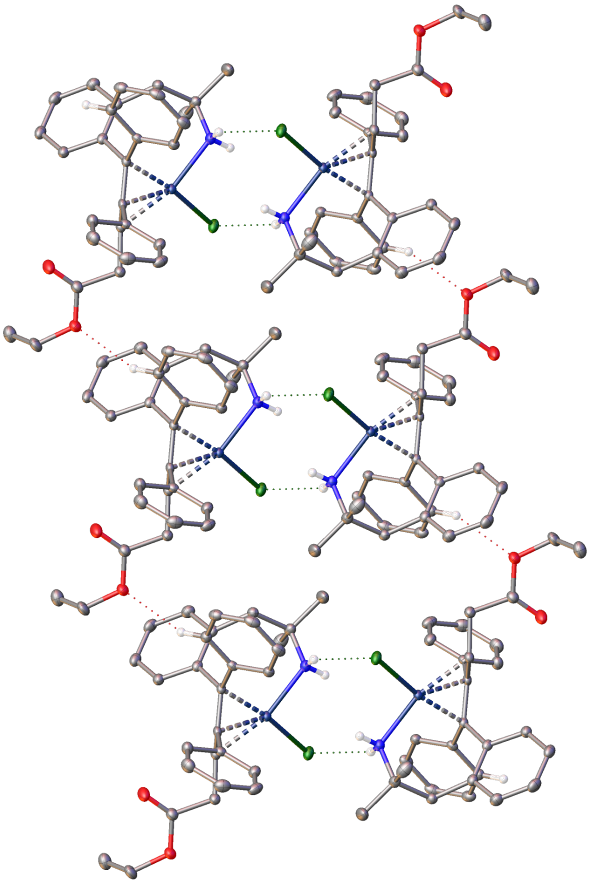

Figure S15. X-ray packing view (50 % probability) of *syn*-**2b** showing the double chains parallel to the *a* axis formed through hydrogen bonds.

Table S11. Hydrogen bonds for complex *syn*-**3b**·CH_2Cl_2 (Å and deg).

D–H···A	d(D–H)	d(H···A)	d(D···A)	<(DHA)
N(1)–H(01A)···O(2)	0.86(3)	2.30(3)	3.143(3)	170(3)
N(2)–H(02A)···O(1)	0.89(3)	2.09(4)	2.925(3)	155(3)
N(1')–H(01D)···O(6)#1	0.81(3)	2.35(3)	3.153(3)	172(3)
N(2')–H(02C)···O(5)#1	0.86(3)	2.16(3)	2.942(3)	153(3)
N(2')–H(02D)···O(6)#2	0.80(3)	2.42(3)	3.200(3)	165(3)

Symmetry transformations used to generate equivalent atoms: #1 -x+1,-y,-z #2 x+1,y,z

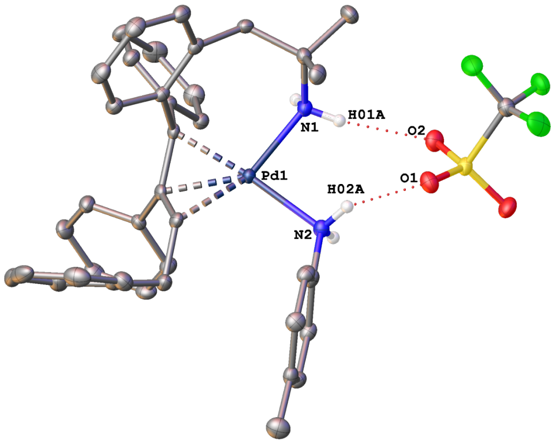

Figure S16. Thermal ellipsoid plot (50 % probability) of *syn*-**3b**·CH_2Cl_2 (molecule A) showing the hydrogen bonds between the cation and the triflate group.

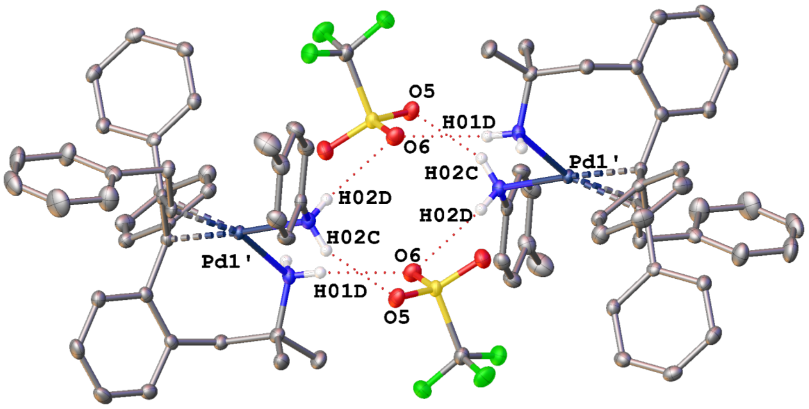

Figure S17. Thermal ellipsoid plot (50 % probability) of **3b**·CH_2Cl_2 (molecule A') showing the dimers formed through hydrogen bonds.

Table S12. Hydrogen bonds for compound **6b**·H_2O (Å and deg).

D–H···A	d(D–H)	d(H···A)	d(D···A)	<(DHA)
O(4)–H(04A)···O(3)#1	0.87(2)	1.89(2)	2.760(2)	177(2)
N(1)–H(01B)···O(4)#2	0.907(19)	1.85(2)	2.753(2)	171(2)
N(1)–H(01A)···O(1)#3	0.905(19)	1.95(2)	2.854(2)	176(2)
O(4)–H(04B)···O(1)	0.86(2)	1.99(2)	2.788(2)	154(3)
N(1)–H(01C)···O(2)	0.93(3)	1.94(3)	2.855(2)	164(2)

Symmetry transformations used to generate equivalent atoms:

#1 -x,y+1/2,-z+1/2 #2 x,-y+3/2,z-1/2 #3 -x,y-1/2,-z+1/2

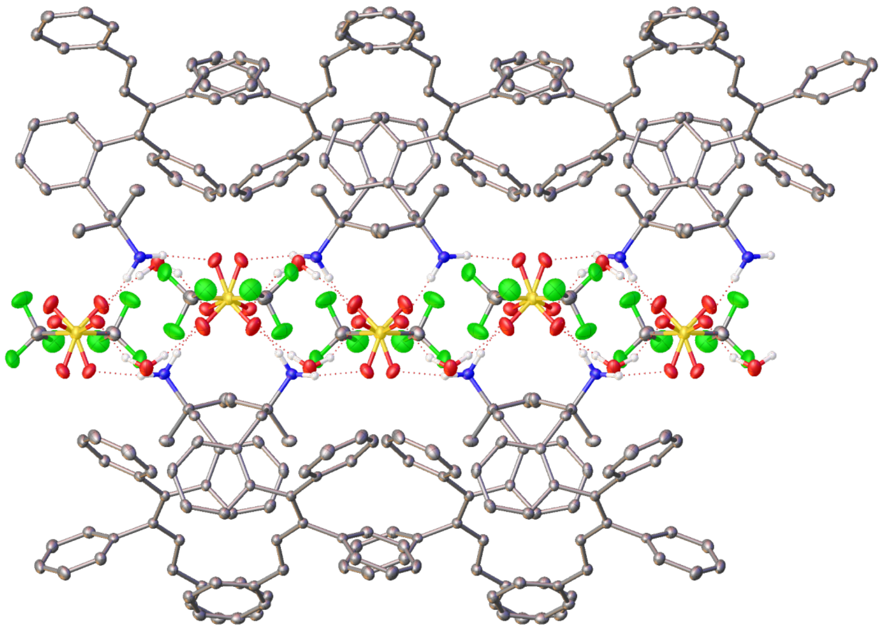

Figure S18. X-ray packing view (50 % probability) of **6b**·H_2O showing the double chains parallel to the *b* axis formed through hydrogen bonds.

Table S13. Hydrogen bonds for compound **7c**·$CHCl_3$ (Å and deg).

D–H···A	d(D–H)	d(H···A)	d(D···A)	<(DHA)
C(99)–H(99)···Cl(1)#1	1.00	2.43	3.409(2)	167.4

Symmetry transformations used to generate equivalent atoms: #1 x+1/2,-y+1/2,z-1/2

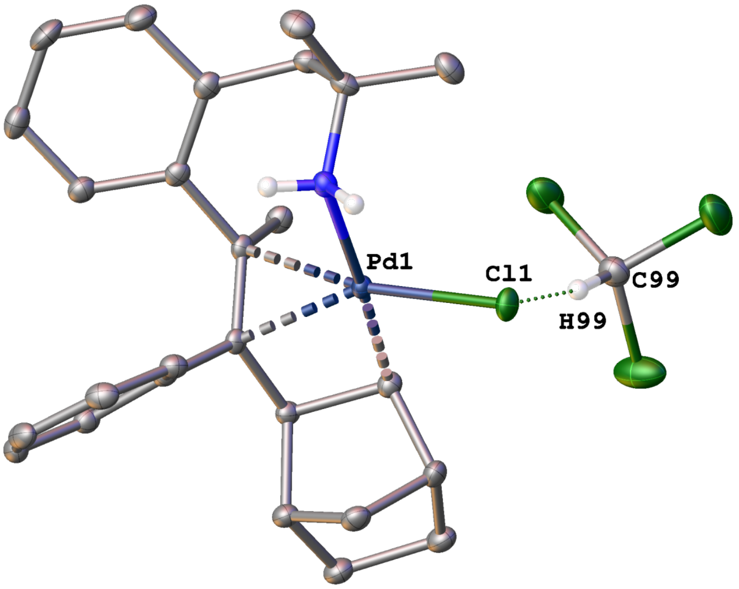

Figure S19. Thermal ellipsoid plot (50 % probability) of **7c**·$CHCl_3$ showing the hydrogen bond between complex **7c** and the crystallization solvent.

Table S14. Hydrogen bonds for compound **7d**·1/2$CHCl_3$ (Å and deg).

D–H···A	d(D–H)	d(H···A)	d(D···A)	<(DHA)
C(11)–H(11B)···O(2)	0.99	2.46	3.391(4)	156.7

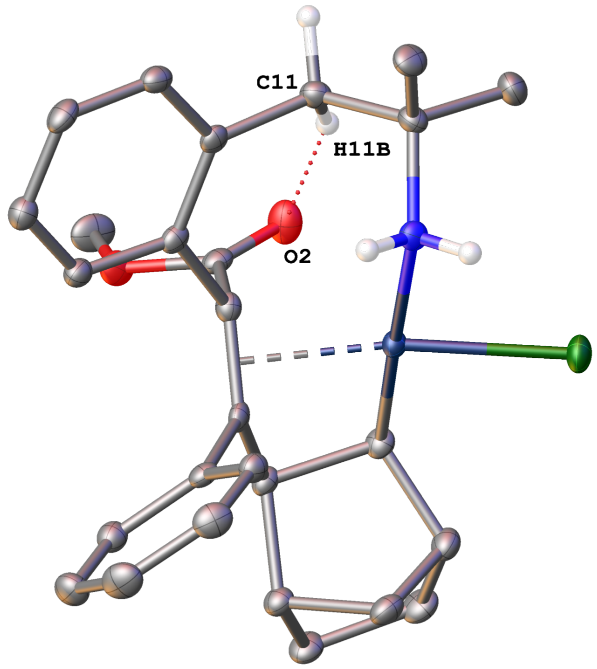

Figure S20. Thermal ellipsoid plot (50 % probability) of **7d**·1/2$CHCl_3$ showing the intramolecular hydrogen bond.

Table S15. Hydrogen bonds for compound **7f** (Å and deg).

D–H···A	d(D–H)	d(H···A)	d(D···A)	<(DHA)
N(1)–H(01A)···O(4)	0.827(18)	2.36(2)	2.956(2)	129(2)
N(1)–H(01B)···Cl(1)#1	0.833(17)	2.80(2)	3.4396(18)	134.7(18)
C(11)–H(11A)···O(3)#2	0.99	2.59	3.495(2)	151.4
C(16)–H(16C)···O(3)#3	0.98	2.59	3.401(3)	140.3

Symmetry transformations used to generate equivalent atoms:

#1 -x+2,-y+2,-z #2 -x+3/2,y-1/2,-z+1/2 #3 -x+2,-y+2,-z+1

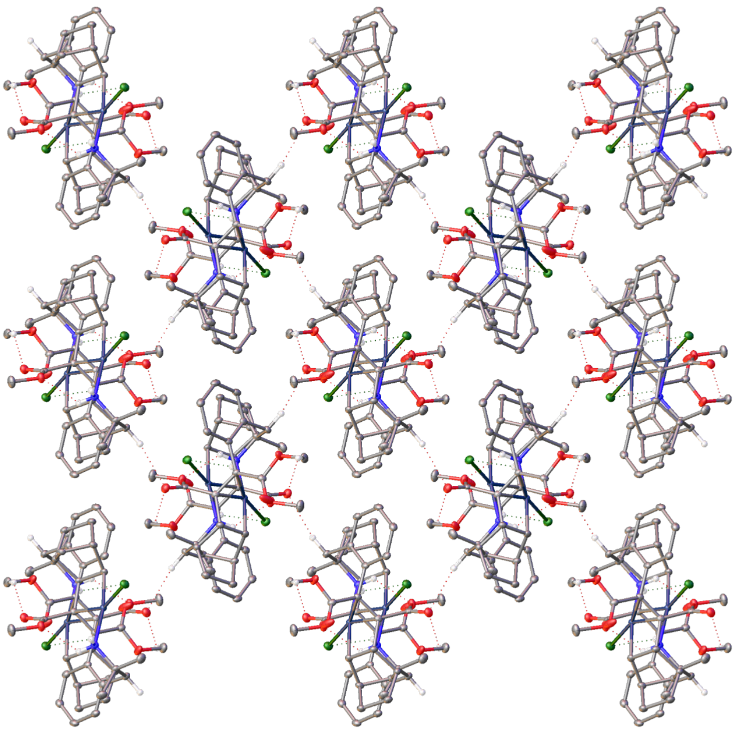

Figure S21. X-ray packing view (30 % probability) of **7f** showing the three-dimensional net, formed through hydrogen bonds, viewed down the crystallographic *c* axis.

Table S16. Hydrogen bonds for compound **8f** (Å and deg).

D–H···A	d(D–H)	d(H···A)	d(D···A)	<(DHA)
N(1)–H(01A)···O(2)	0.908(15)	2.005(16)	2.7930(16)	144.3(15)
N(1)–H(01B)···O(6)#1	0.897(16)	2.007(17)	2.7834(15)	144.0(17)
C(12)–H(12A)···O(6)#2	0.98	2.53	3.4215(19)	151.8
C(23)–H(23B)···O(5)#3	0.98	2.60	3.4925(18)	151.8
C(6)–H(6)···O(7)	0.95	2.56	3.4679(17)	159.3
C(21)–H(21B)···O(5)	0.98	2.46	3.2902(18)	141.8

Symmetry transformations used to generate equivalent atoms:

#1 x,y-1,z   #2 -x,-y+1,-z   #3 -x,-y+1,-z+1

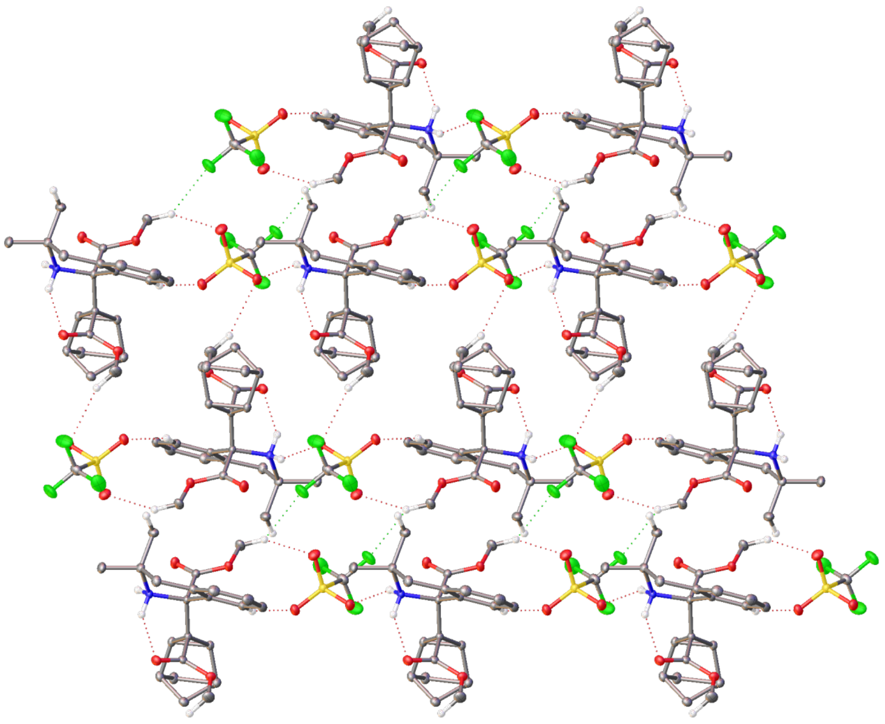

Figure S22. X-ray packing view (50 % probability) of **8f** showing the three-dimensional net, formed through hydrogen bonds, viewed down the crystallographic *a* axis.

Table S17. Hydrogen bonds for compound **9d** (Å and deg).

D–H···A	d(D–H)	d(H···A)	d(D···A)	<(DHA)
N(1)–H(01A)···O(3)#1	0.893(18)	2.09(2)	2.921(3)	154(3)
N(1)–H(01B)···O(1)#2	0.891(19)	2.28(2)	3.085(3)	150(3)
N(1)–H(01C)···O(6)#3	0.892(19)	1.913(19)	2.797(3)	171(3)
C(9)–H(9A)···F(3)#4	0.98	2.43	3.354(3)	158.0
C(9)–H(9B)···O(1)#2	0.98	2.57	3.384(3)	140.5
C(10)–H(10A)···O(3)#1	0.98	2.43	3.209(3)	136.6

Symmetry transformations used to generate equivalent atoms:

#1 -x+3/2,y+1/2,z #2 x+1/2,-y+1/2,-z #3 -x+2,-y+1,-z #4 x,-y+1/2,z-1/2

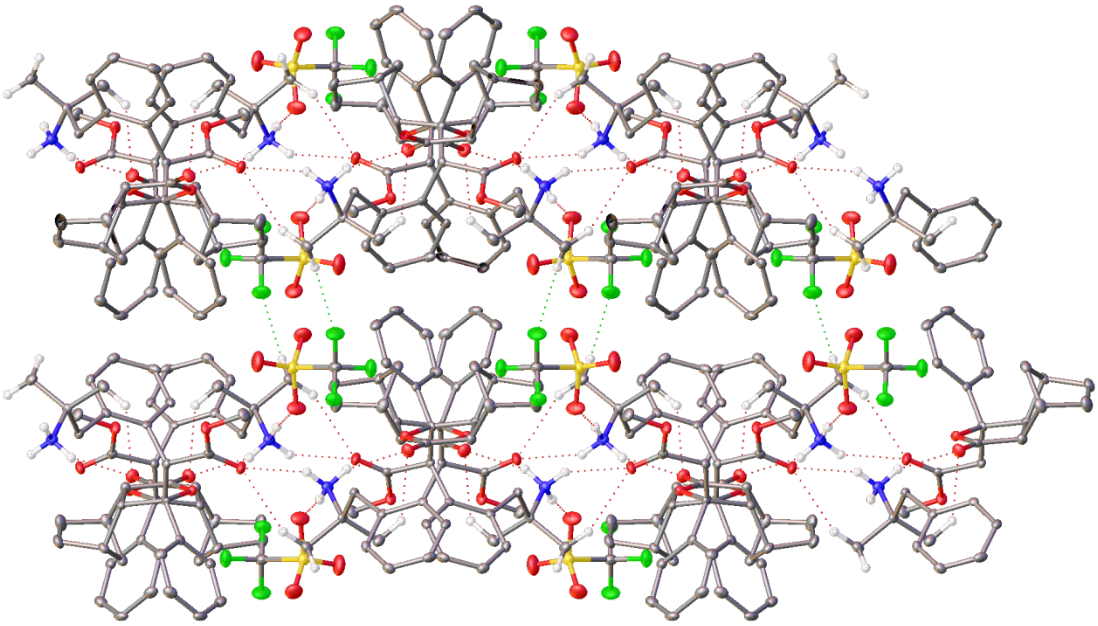

Figure S23. X-ray packing view (50 % probability) of **9d** showing showing the three-dimensional net, formed through hydrogen bonds, viewed down the crystallographic *b* axis.

Table S18. Hydrogen bonds for compound **9f**·Et_2O (Å and deg).

D–H···A	d(D–H)	d(H···A)	d(D···A)	<(DHA)
N(1)–H(01B)···O(9)	0.87(2)	2.06(2)	2.916(2)	167.1(19)
C(7)–H(7A)···O(6)	0.99	2.37	3.1974(19)	140.1
N(1)–H(01A)···O(3)#1	0.91(2)	1.94(2)	2.8063(19)	158.7(16)
N(1)–H(01C)···O(83)#2	0.90(2)	2.19(2)	2.9997(18)	150.0(18)
C(9)–H(9A)···O(7)#3	0.98	2.52	3.398(2)	148.3
C(81)–H(81C)···O(8)#4	0.98	2.57	3.468(3)	153.1

Symmetry transformations used to generate equivalent atoms:

#1 x+1/2,-y+1/2,z-1/2 #2 x,y,z-1 #3 x-1,y,z #4 x,y,z+1

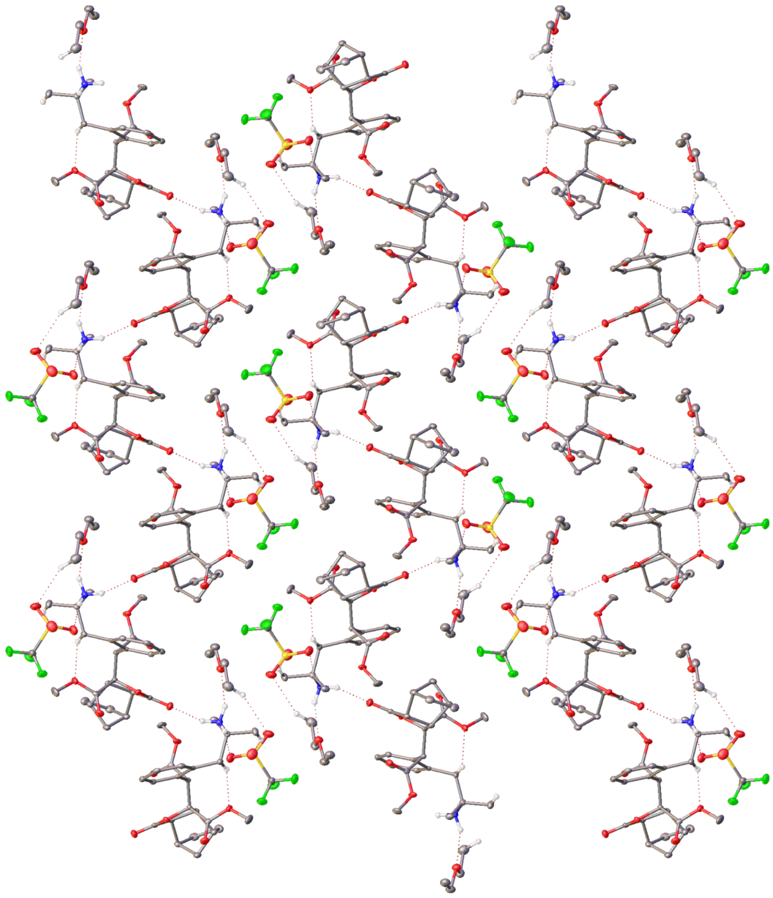

Figure S24. X-ray packing view (50 % probability) of **9f**·Et_2O showing the layers paralel to the ab plane, viewed down the crystallographic *c* axis.

Table S19. Hydrogen bonds for compound **10c**·H_2O (Å and deg).

D–H···A	d(D-H)	d(H···A)	d(D···A)	<(DHA)
C(7)–H(7A)···O(1)	0.99	2.49	3.389(2)	150.7
N(1)–H(01A)···O(1)	0.922(16)	2.226(18)	3.064(2)	150.8(19)
N(1)–H(01A)···O(4)	0.922(16)	2.53(2)	3.104(2)	120.8(17)
N(1)–H(01B)···O(99)#1	0.918(16)	1.867(17)	2.774(2)	169(2)
N(1)–H(01C)···O(3)#1	0.926(16)	2.014(17)	2.912(2)	163(2)
O(2)–H(2)···O(4)	0.84	1.87	2.710(2)	173.1
O(99)–H(99A)···O(5)#2	0.842(10)	2.158(15)	2.890(2)	145(2)
O(99)–H(99B)···O(1)	0.843(9)	2.114(14)	2.881(2)	151(2)

Symmetry transformations used to generate equivalent atoms:

#1 -x+1,-y,-z+1   #2 x+1,y,z

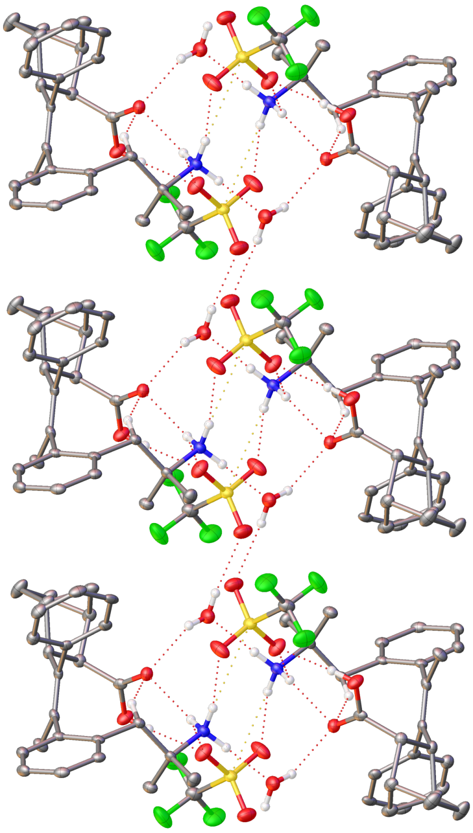

Figure S25. X-ray packing view (50 % probability) of **10c**·H_2O showing the double chains paralel to the *a* axis formed through hydrogen bonds.

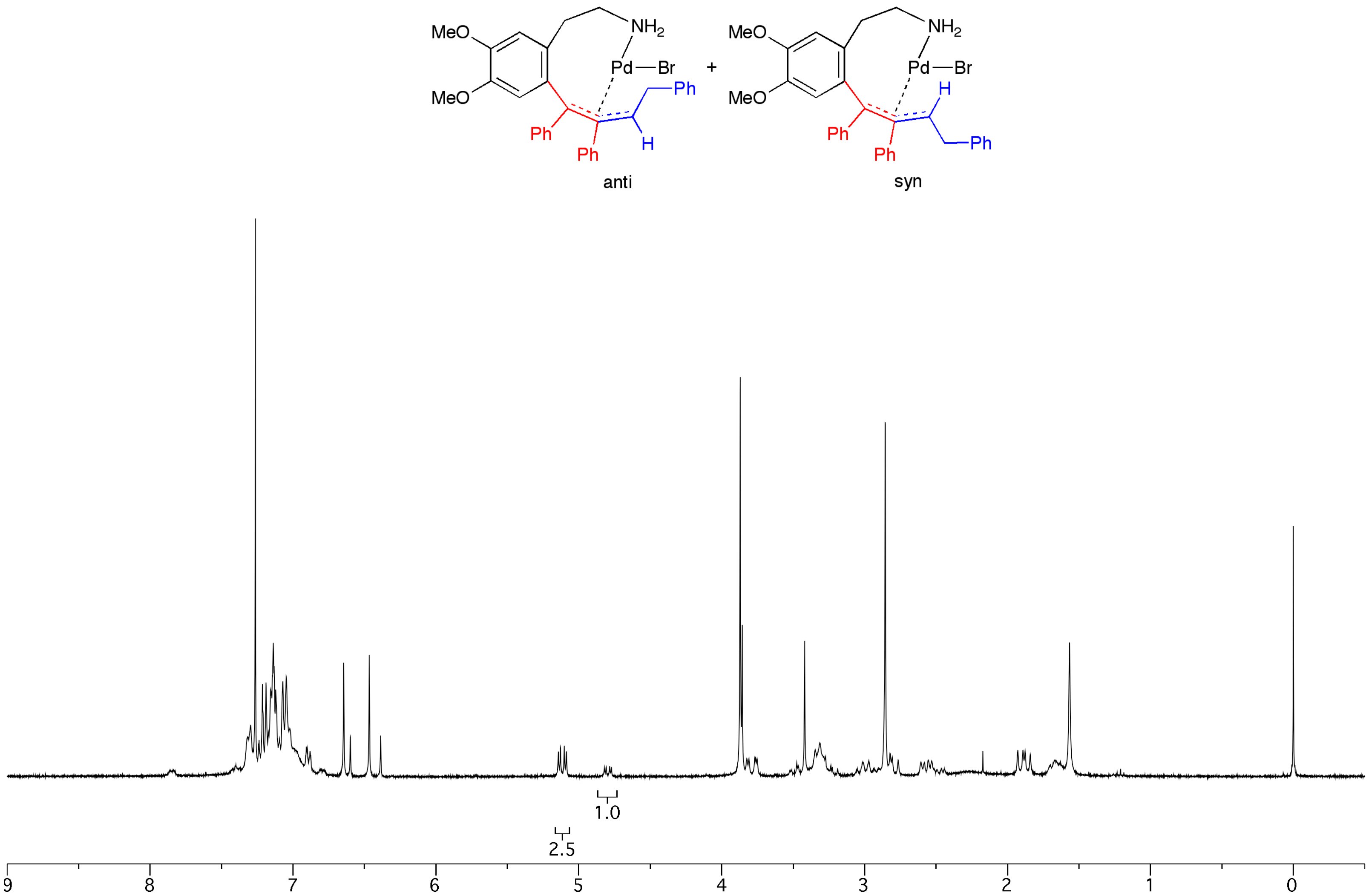

Figure S26. ^{1}H NMR spectrum of a 2.5:1 mixture of *anti/syn*-**1a** (300.1 MHz, $CDCl_3$, 25 ºC)

(From left to right, the asteriks indicate the signals corresponding to $CHCl_3$, and H_2O)

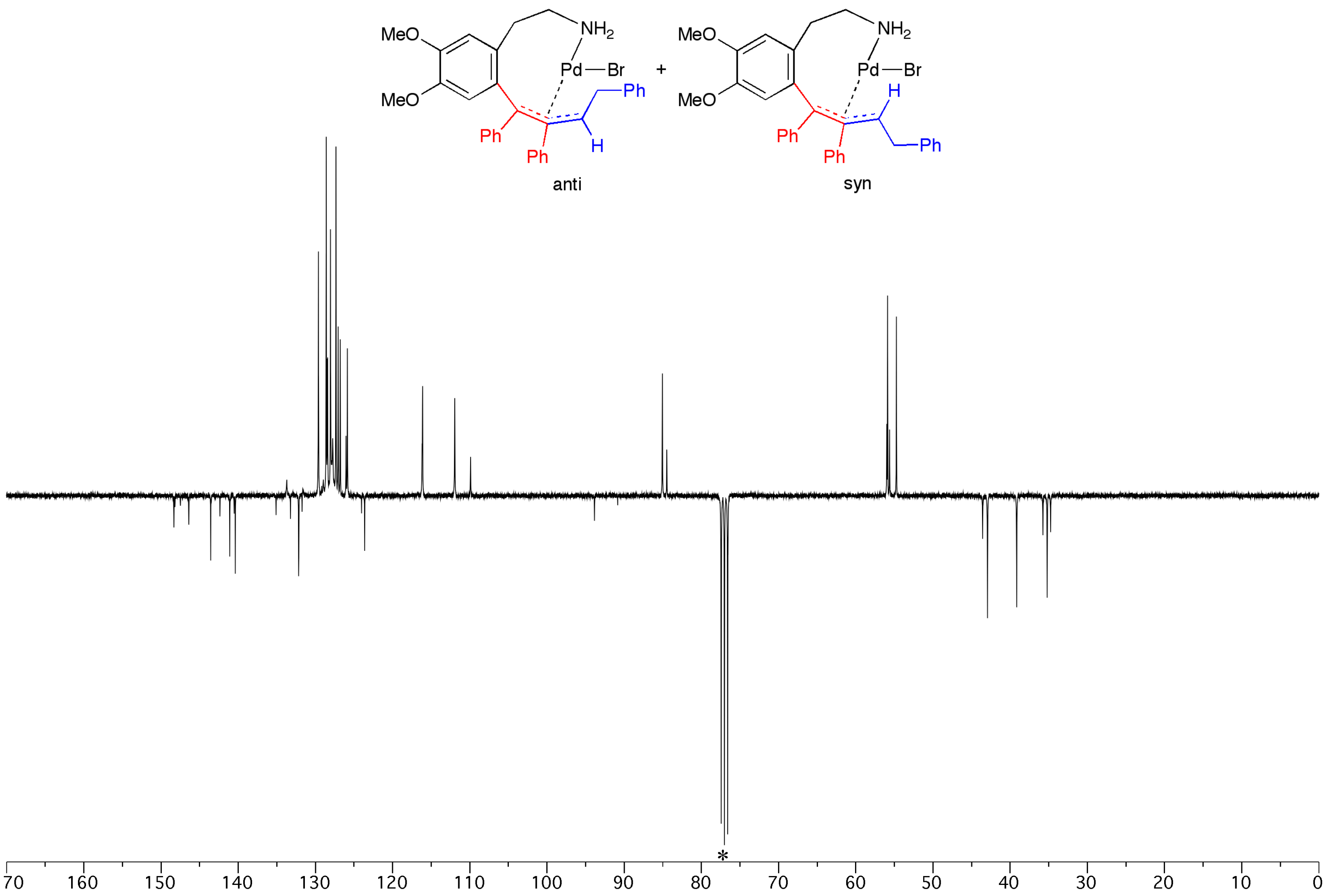

Figure S27. $^{13}C\{^{1}H\}$ APT NMR spectrum of 2.5:1 mixture of *anti/syn*-**1a** (75.5 MHz, $CDCl_3$, 25 ºC)

(The asterik indicates the signal corresponding to $CDCl_3$)

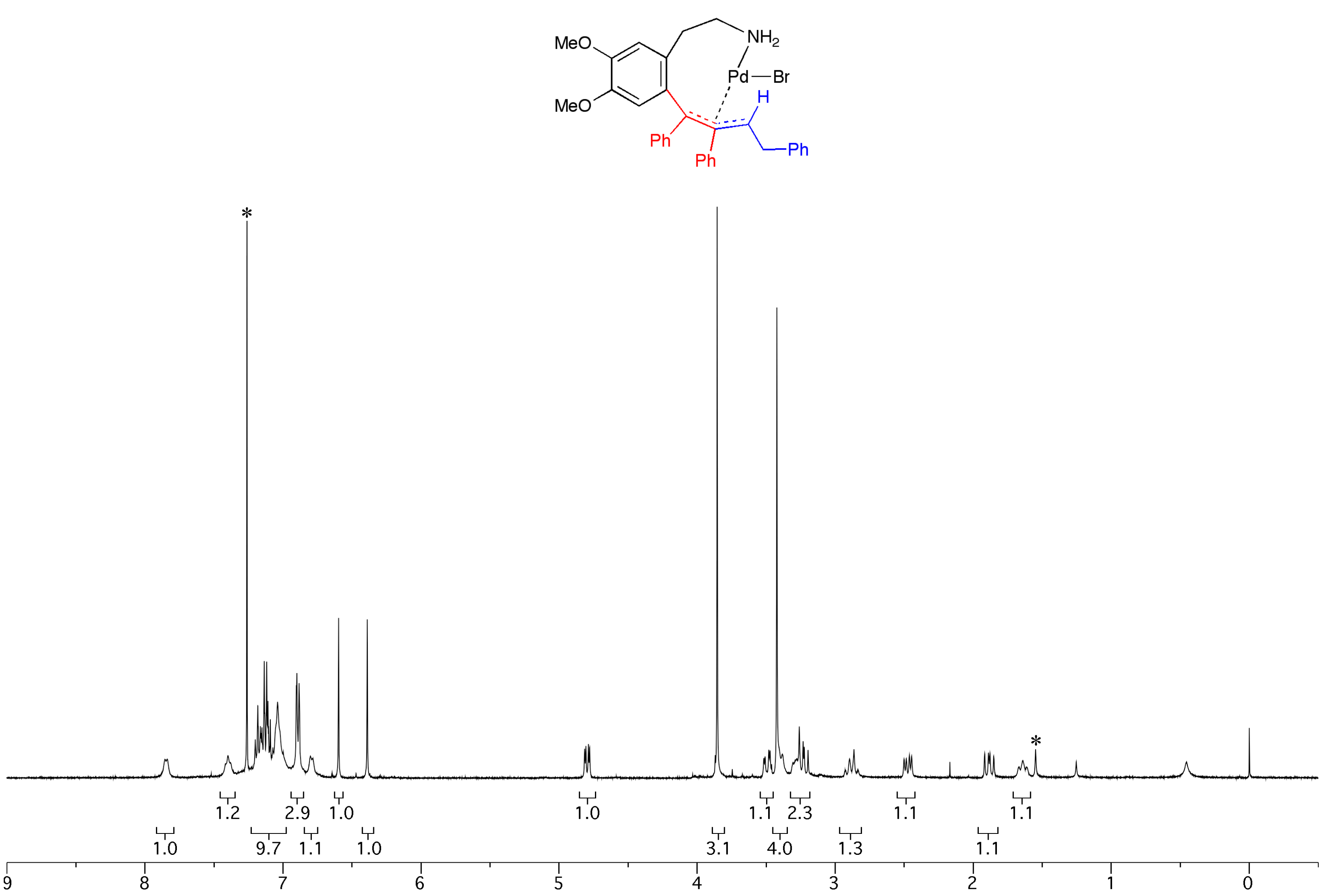

Figure S28. ^1H NMR spectrum of *syn*-**1a** (400.9 MHz, $CDCl_3$, 25 ºC)

(From left to right, the asteriks indicate the signals corresponding to $CHCl_3$, and H_2O)

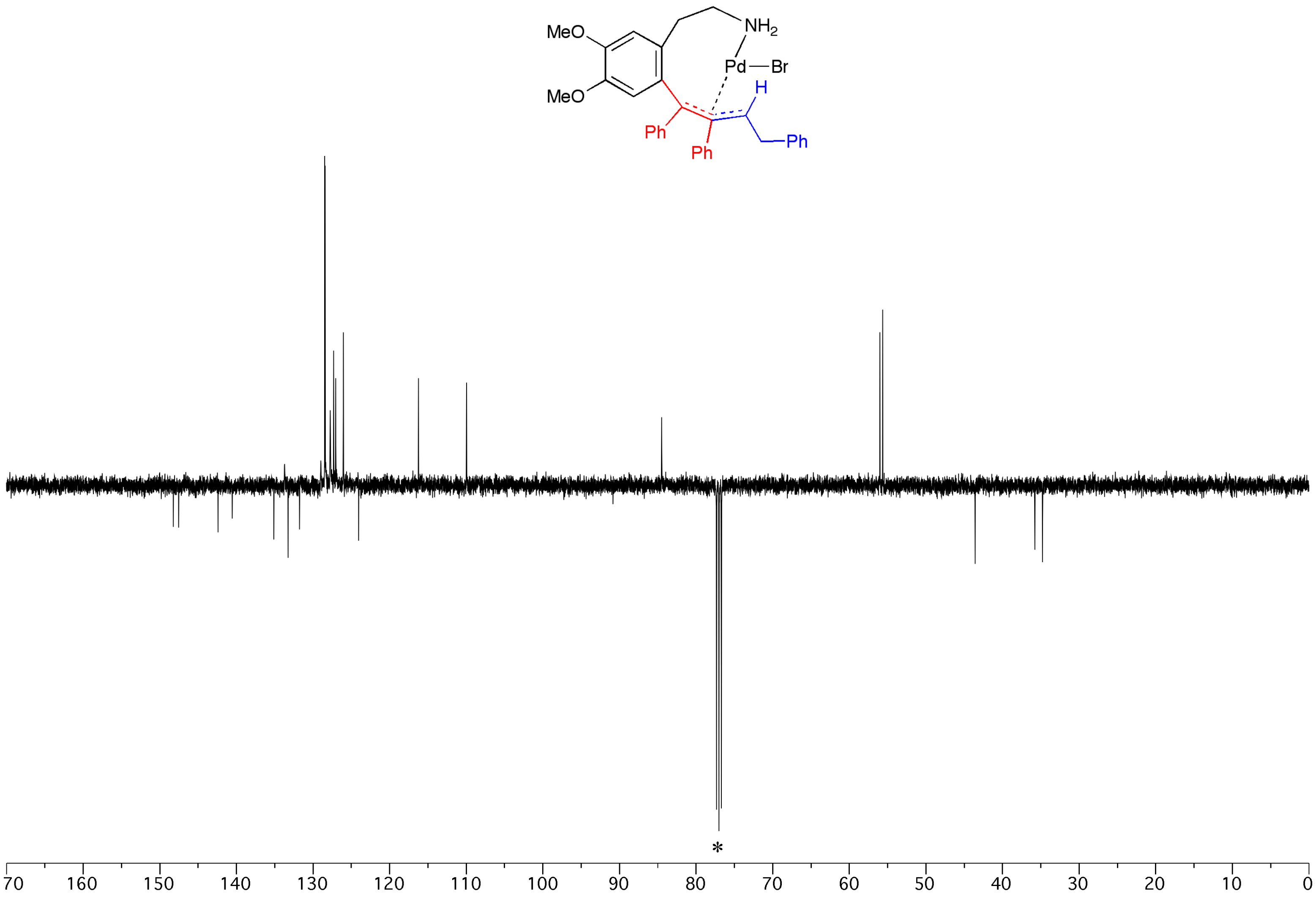

Figure S29. $^{13}C\{^1H\}$ APT NMR spectrum of *syn*-**1a** (100.8 MHz, $CDCl_3$, 25 ºC)

(The asterik indicates the signal corresponding to $CDCl_3$)

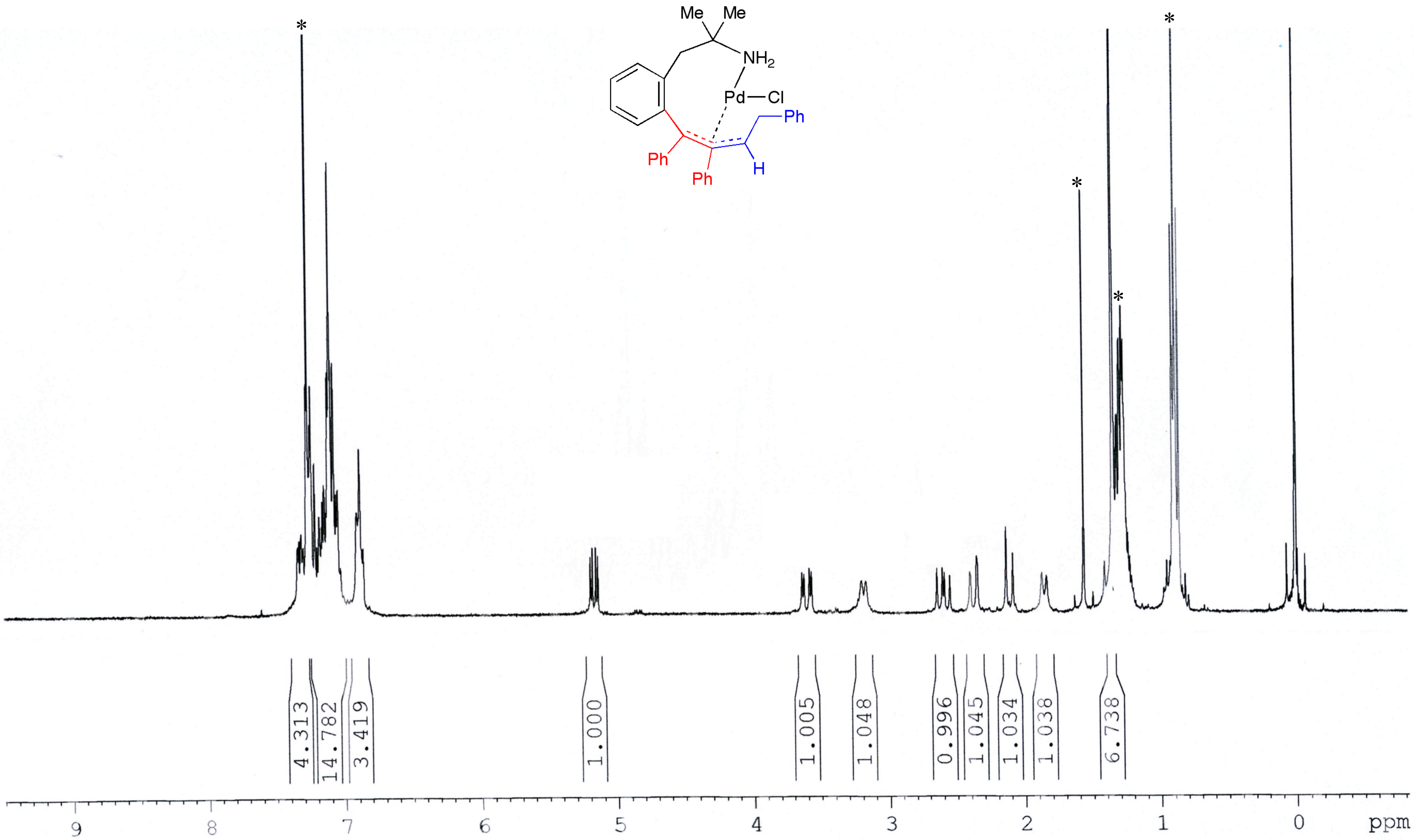

Figure S30. ^{1}H NMR spectrum of *anti*-**1b** (300.1 MHz, $CDCl_3$, 25 ºC)

(From left to right, the asteriks indicate the signals corresponding to $CHCl_3$, H_2O, and *n*-pentane)

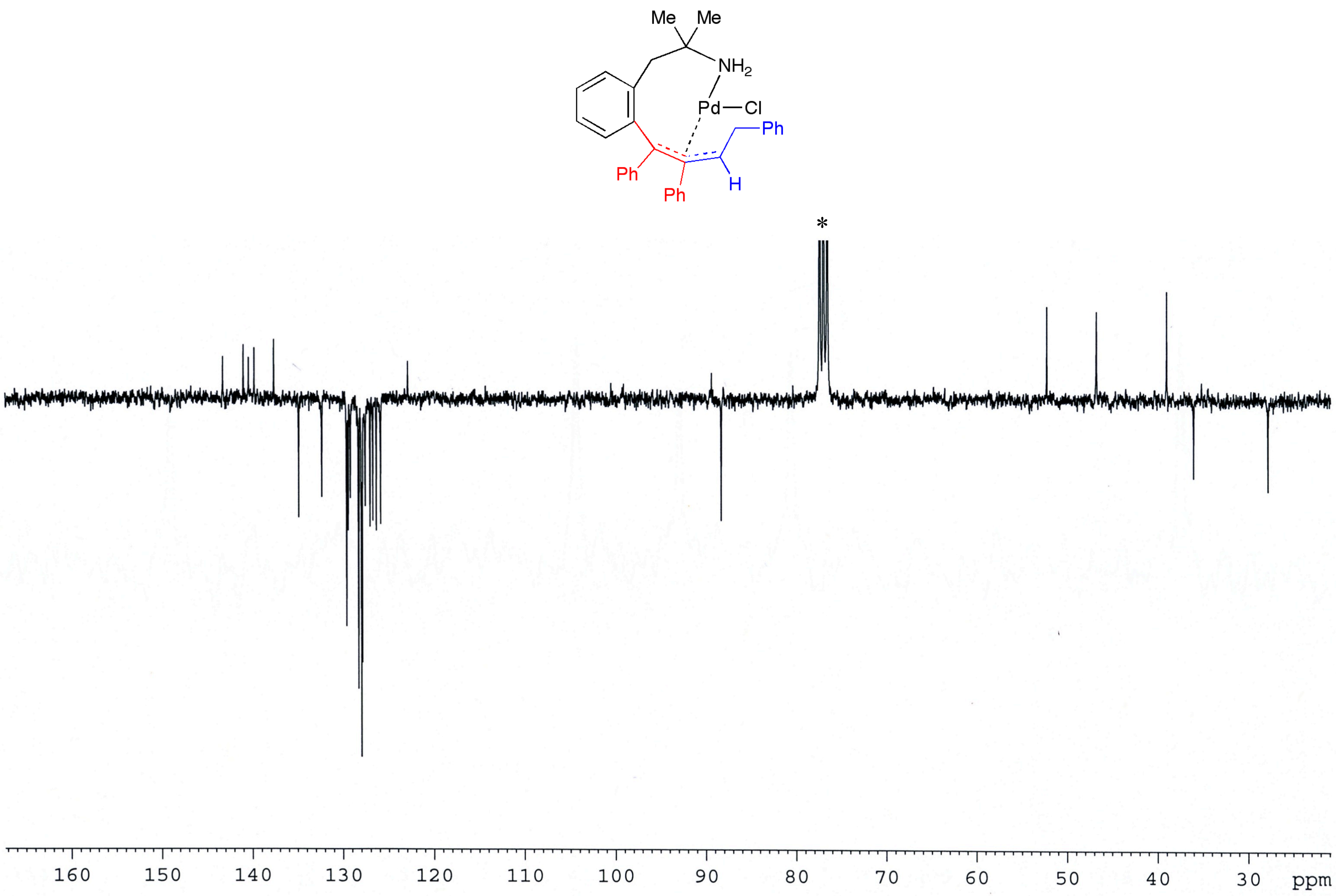

Figure S31. $^{13}C\{^{1}H\}$ APT NMR spectrum of *anti*-**1b** (75.5 MHz, $CDCl_3$, 25 ºC)

(The asterik indicates the signal corresponding to $CDCl_3$)

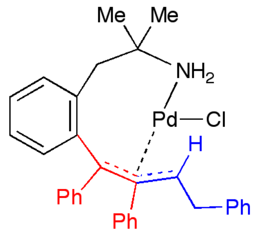

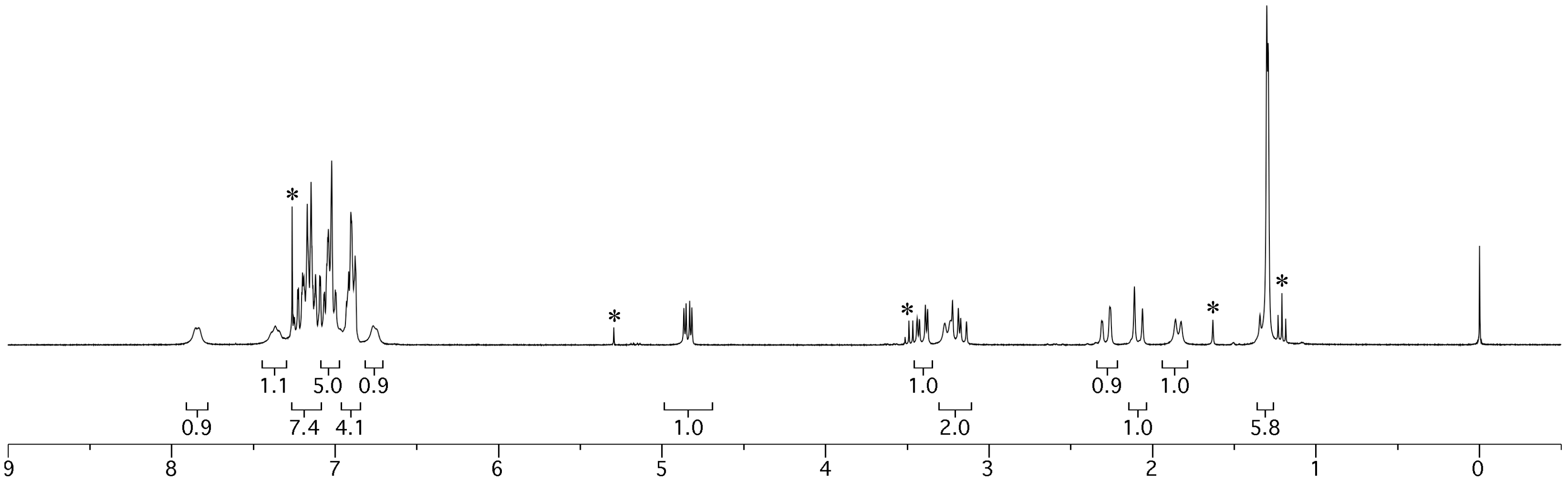

Figure S32. ^{1}H NMR spectrum of *syn*-**1b** (300.1 MHz, $CDCl_3$, 25 ºC)

(From left to right, the asteriks indicate the signals corresponding to $CHCl_3$, CH_2Cl_2, Et_2O, H_2O, and Et_2O)

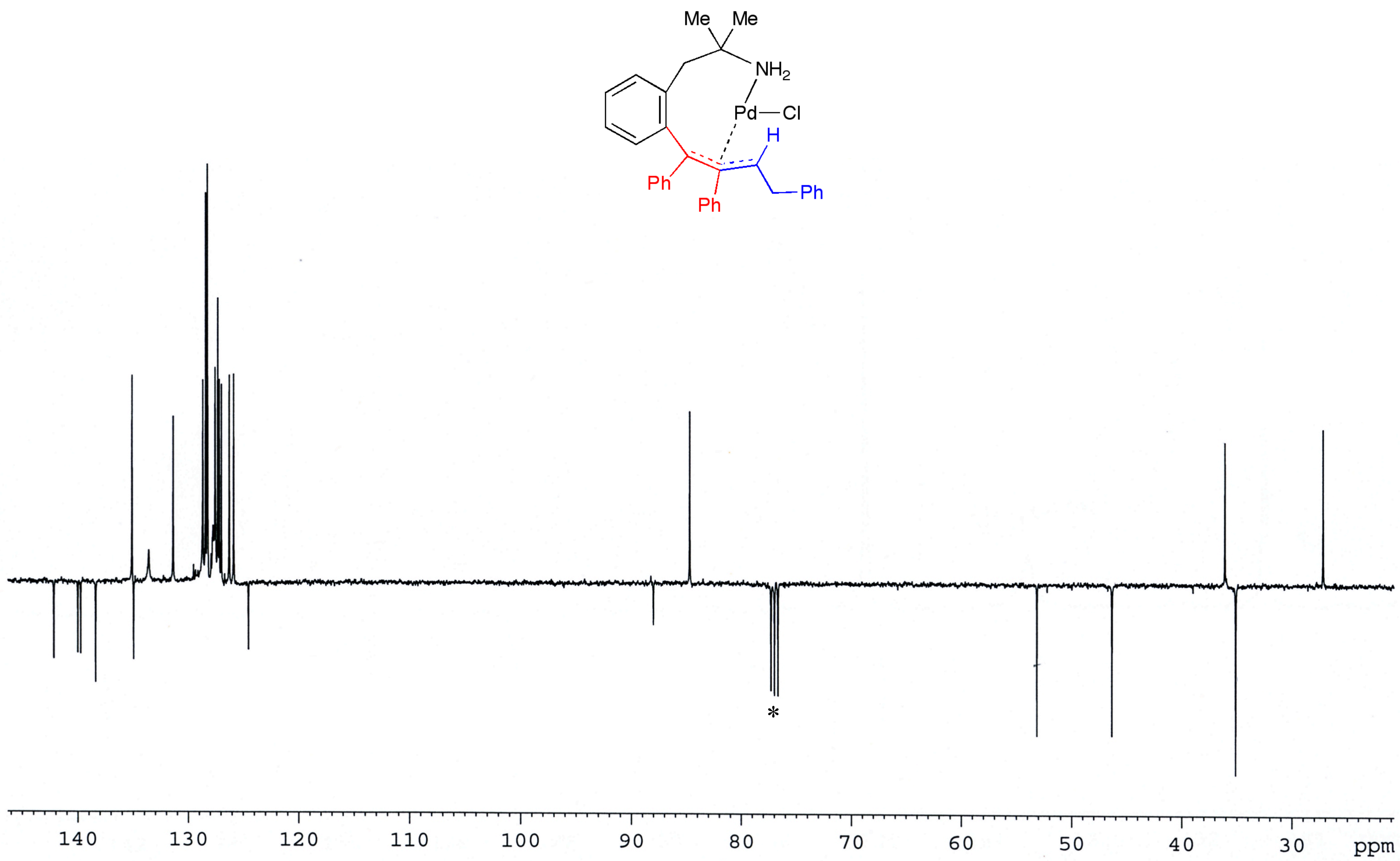

Figure S33. $^{13}C\{^{1}H\}$ APT NMR spectrum of *syn*-**1b** (100.8 MHz, $CDCl_3$, 25 ºC)

(The asterik indicates the signal corresponding to $CDCl_3$)

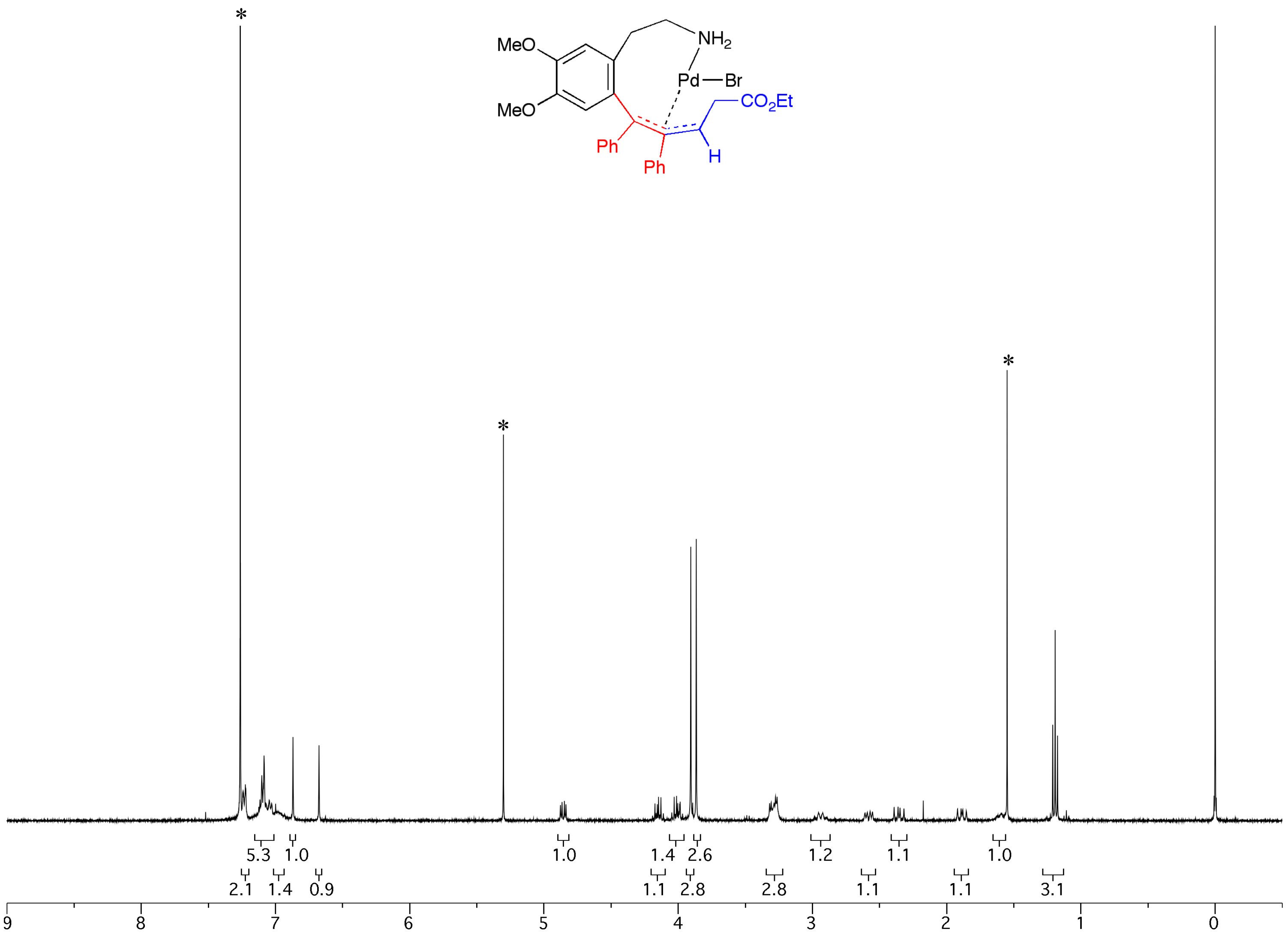

Figure S34. ^{1}H NMR spectrum of *anti*-**2a** (400.9 MHz, $CDCl_3$, 25 ºC)

(From left to right, the asteriks indicate the signals corresponding to $CHCl_3$, CH_2Cl_2, and H_2O)

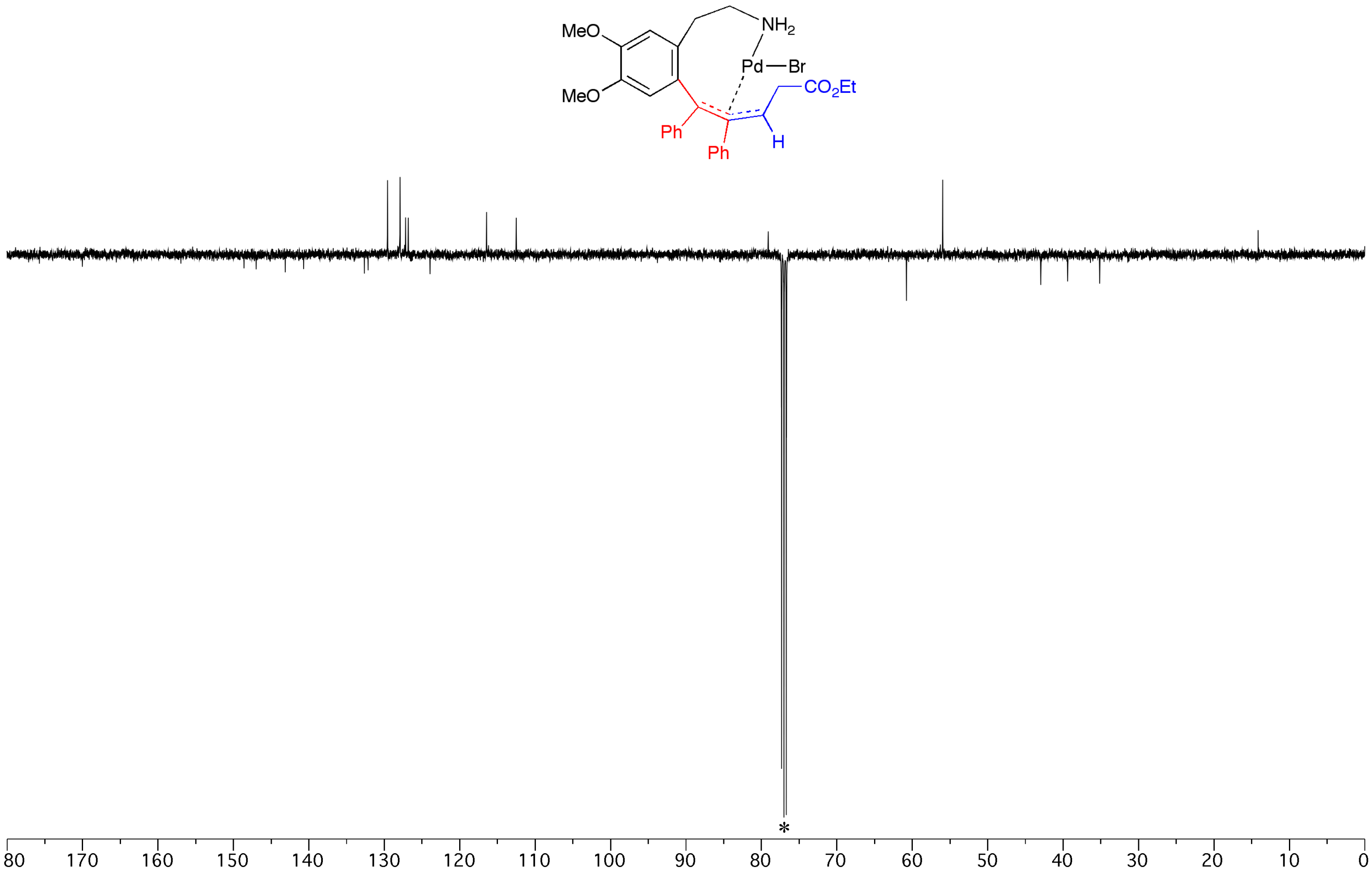

Figure S35. $^{13}C\{^{1}H\}$ APT NMR spectrum of *anti*-**2a** (100.8 MHz, $CDCl_3$, 25 ºC)

(The asterik indicates the signal corresponding to $CDCl_3$)

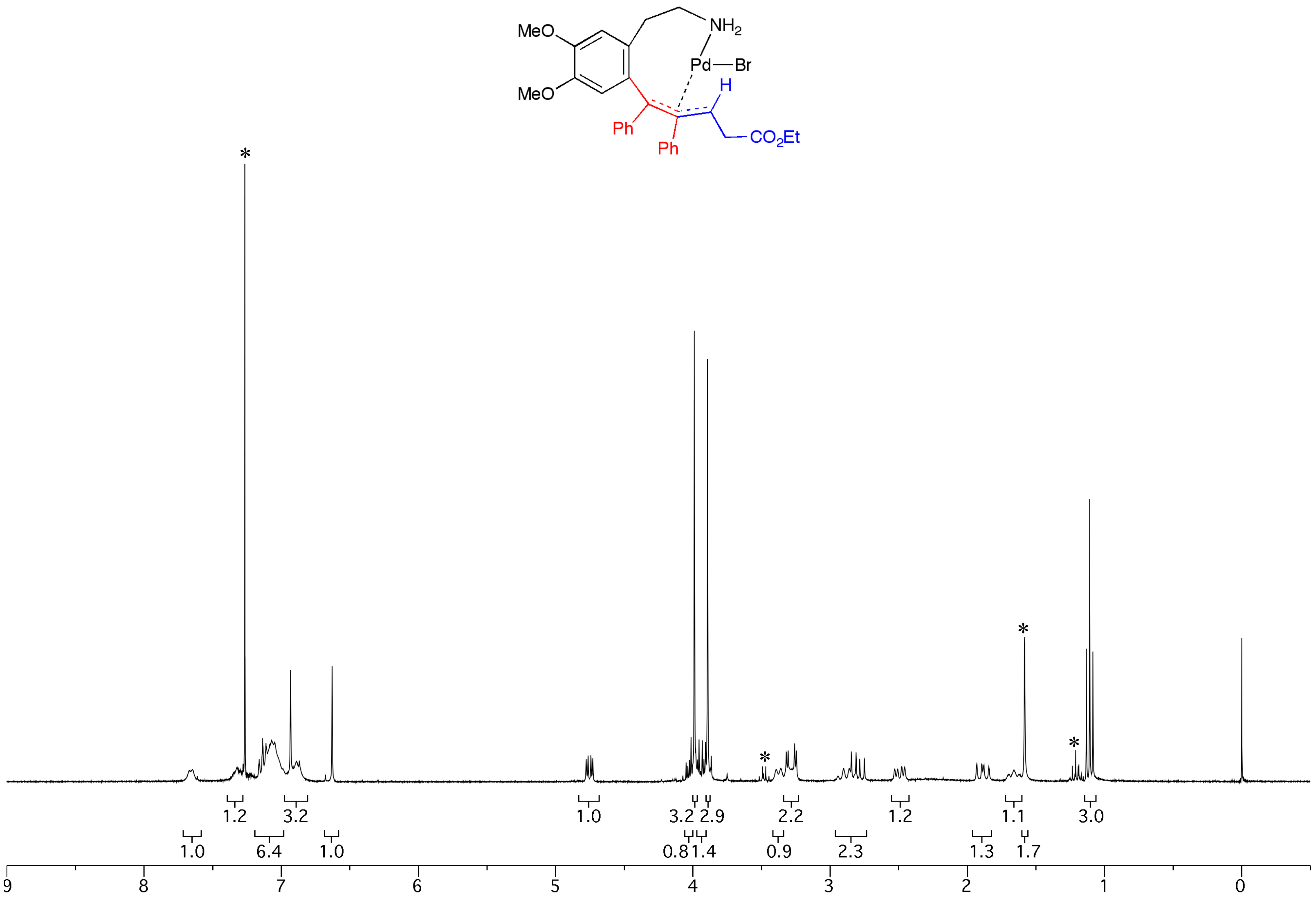

Figure S36. ^{1}H NMR spectrum of *syn*-**2a** (300.1 MHz, $CDCl_3$, 25 ºC)

(From left to right, the asteriks indicate the signals corresponding to $CHCl_3$, Et_2O, H_2O, and Et_2O)

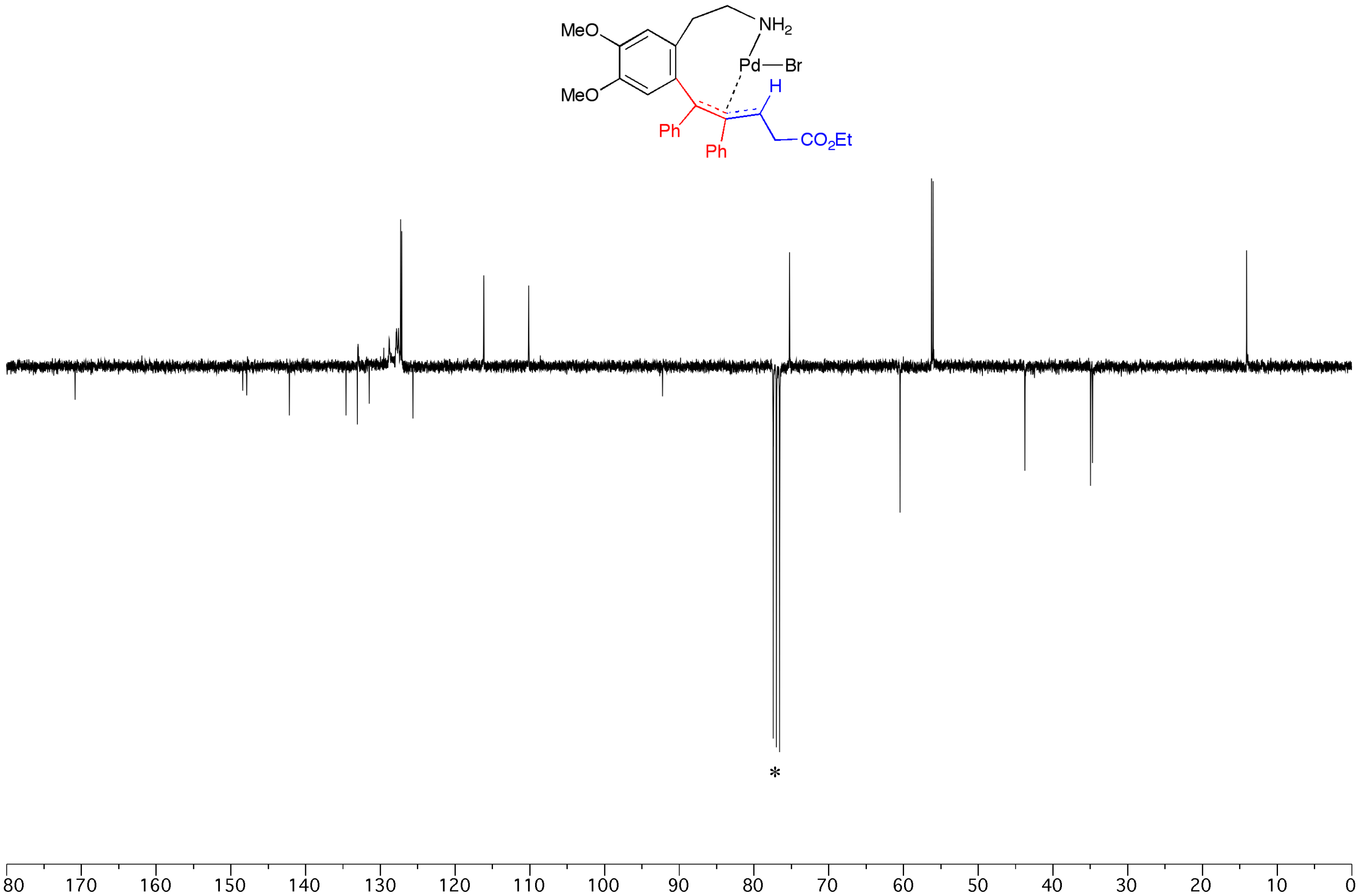

Figure S37. $^{13}C\{^{1}H\}$ APT NMR spectrum of *syn*-**2a** (75.5 MHz, $CDCl_3$, 25 ºC)

(The asterik indicates the signal corresponding to $CDCl_3$)

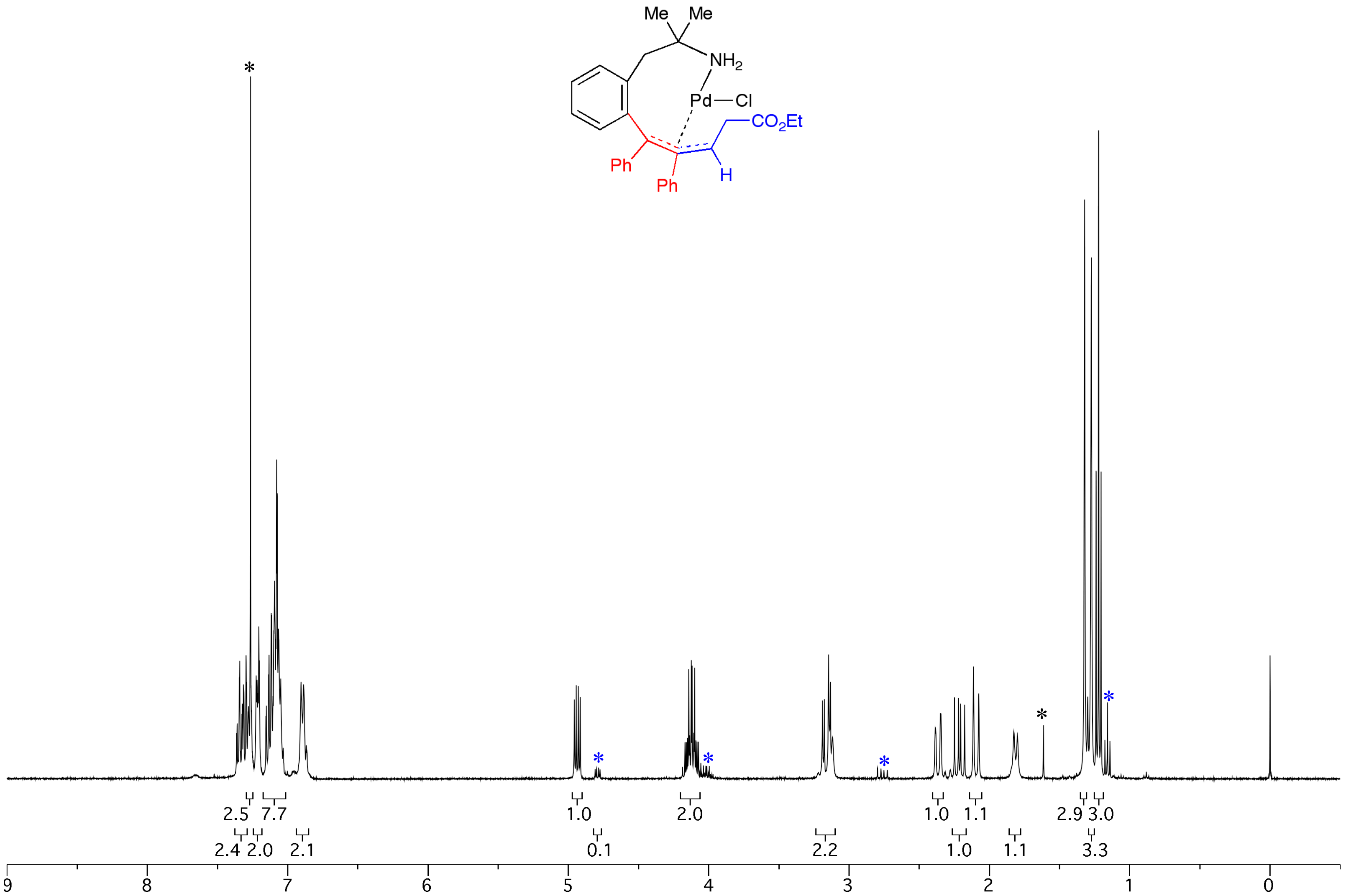

Figure S38. ^{1}H NMR spectrum of *anti*-**2b** (400.9 MHz, $CDCl_3$, 25 ºC)

(From left to right, the black asteriks indicate the signals corresponding to $CHCl_3$, and H_2O; the blue asteriks correspond to the *syn* isomer)

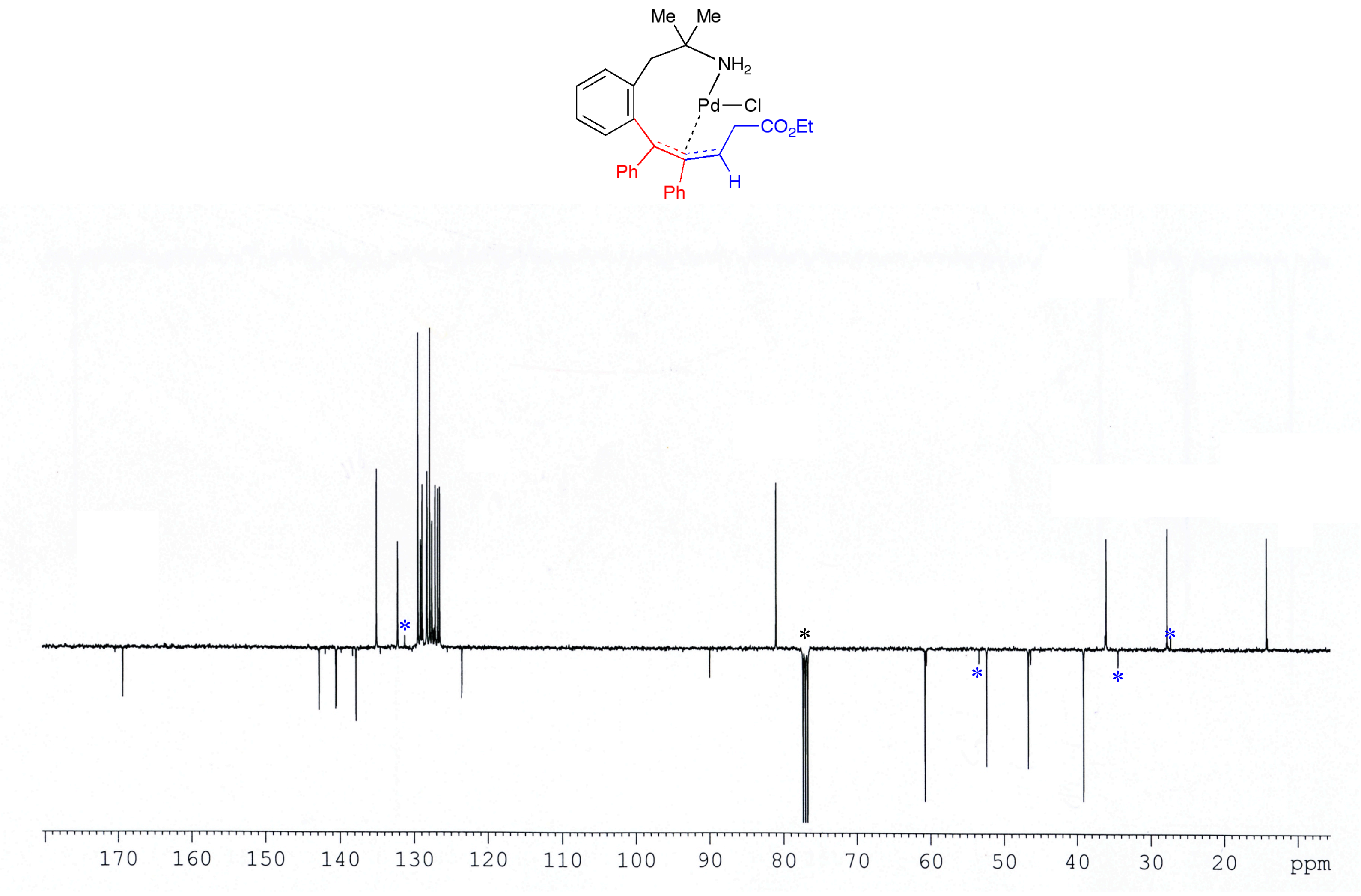

Figure S39. $^{13}C\{^{1}H\}$ APT NMR spectrum of *anti*-**2b** (100.8 MHz, $CDCl_3$, 25 ºC)

(The black asterik indicates the signal corresponding to $CDCl_3$; the blue asteriks correspond to the *syn* isomer)

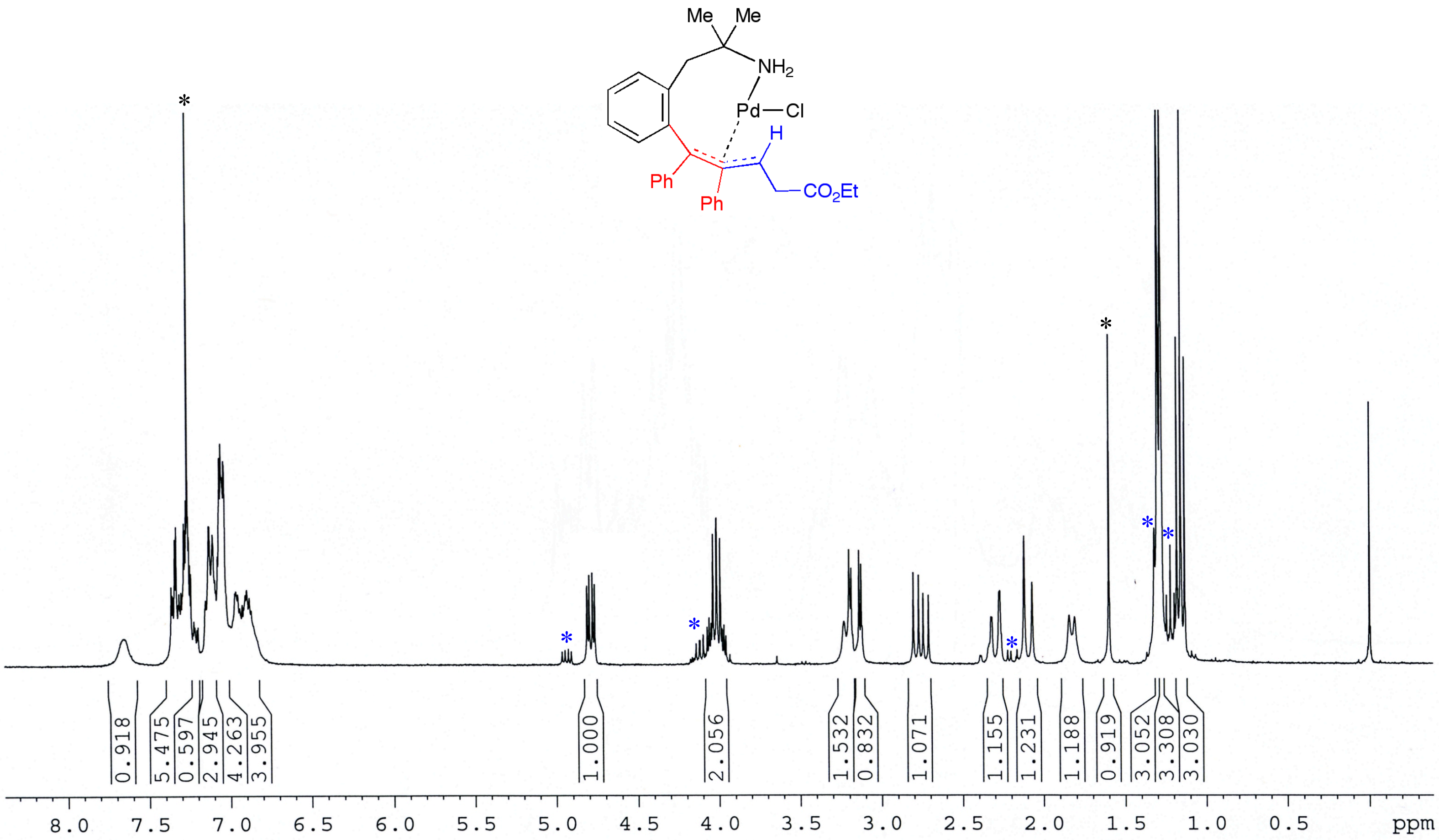

Figure S40. ^{1}H NMR spectrum of *syn*-**2b** (300.1 MHz, $CDCl_3$, 25 ºC)

(From left to right, the black asteriks indicate the signals corresponding to $CHCl_3$, and H_2O; the blue asteriks correspond to the *anti* isomer)

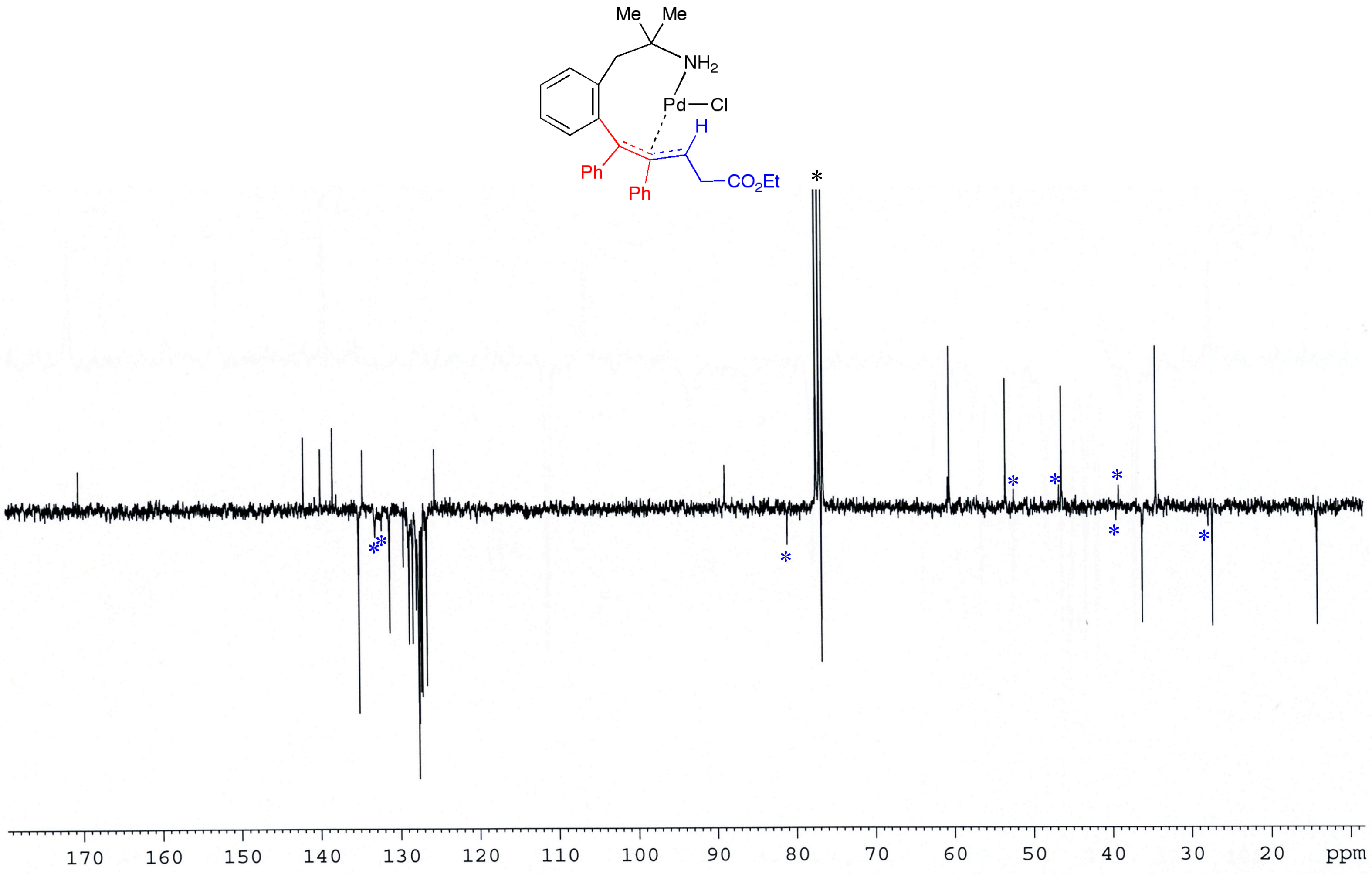

Figure S41. $^{13}C\{^{1}H\}$ APT NMR spectrum of *syn*-**2b** (75.5 MHz, $CDCl_3$, 25 ºC)

(The black asterik indicates the signal corresponding to $CDCl_3$; the blue asteriks correspond to the *anti* isomer)

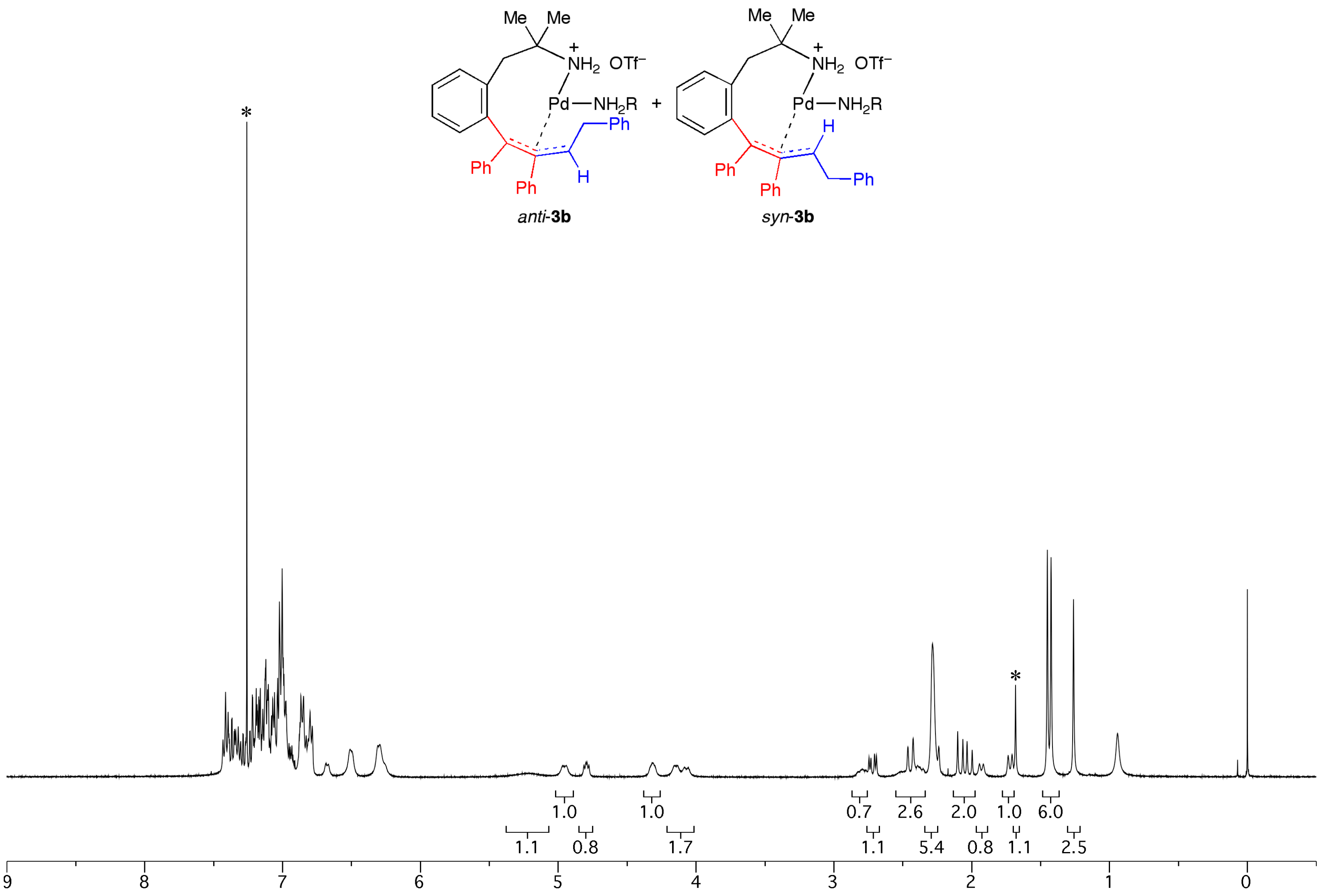

Figure S42. ^{1}H NMR spectrum of a 1:1.33 mixture of *anti*/*syn*-**3b** (400.9 MHz, $CDCl_3$, 25 ºC)

(From left to right, the black asteriks indicate the signals corresponding to $CHCl_3$, and H_2O)

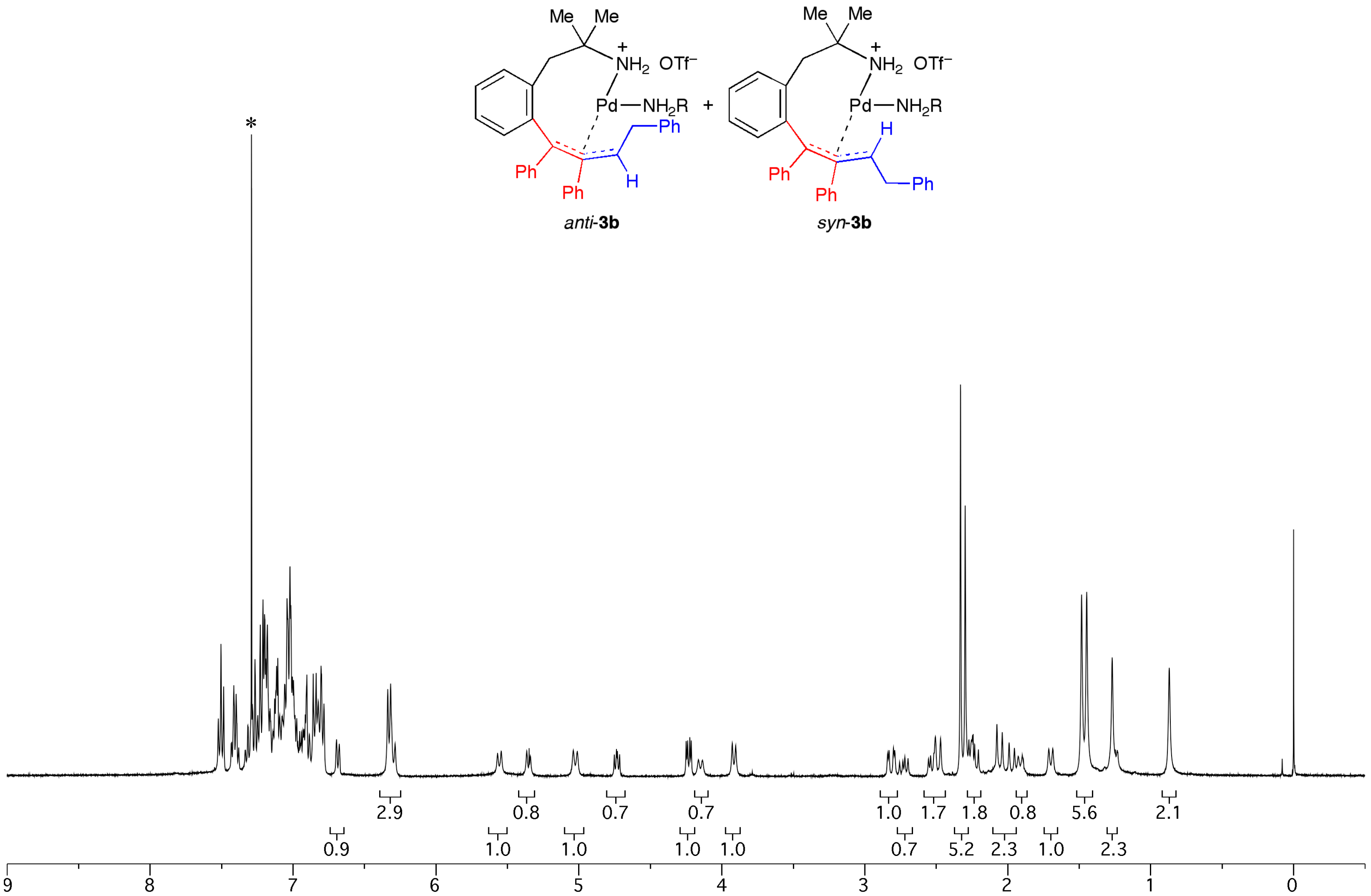

Figure S43. ^{1}H NMR spectrum of a 1:1.33 mixture of *anti*/*syn*-**3b** (400.9 MHz, $CDCl_3$, –40 ºC)

(From left to right, the black asterik indicates the signal corresponding to $CHCl_3$.)

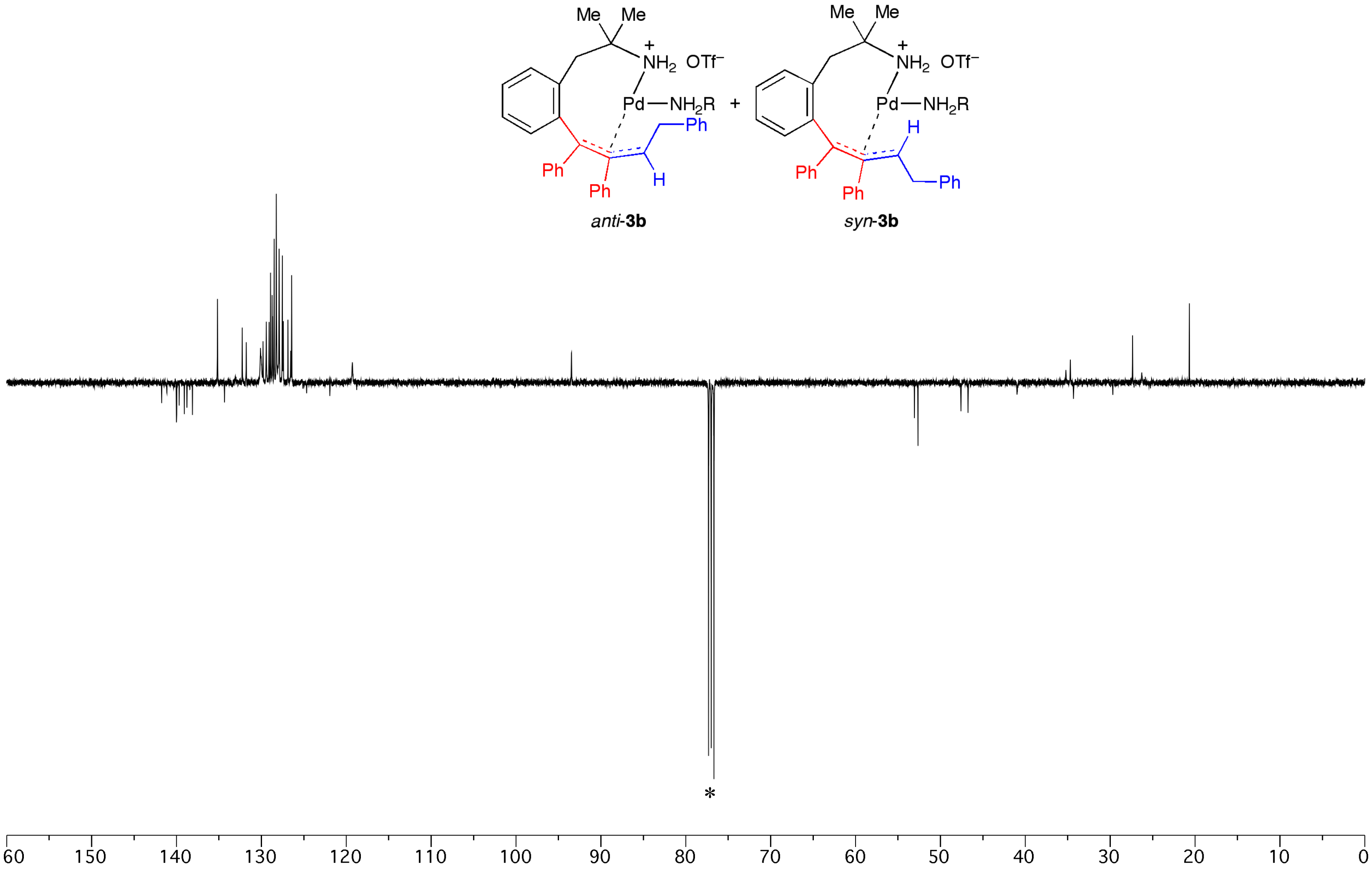

Figure S44. $^{13}C\{^1H\}$ APT NMR spectrum of a 1:1.33 mixture of *anti*/*syn*-**3b** (100.8 MHz, $CDCl_3$, 25 ºC)

(The black asterik indicates the signal corresponding to $CDCl_3$)

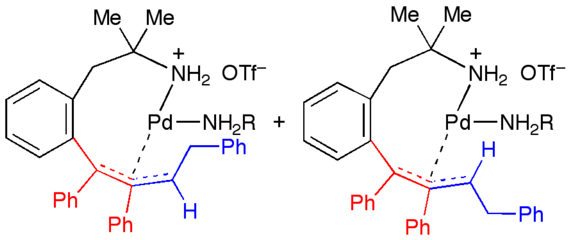

anti-**3b** + *syn*-**3b**

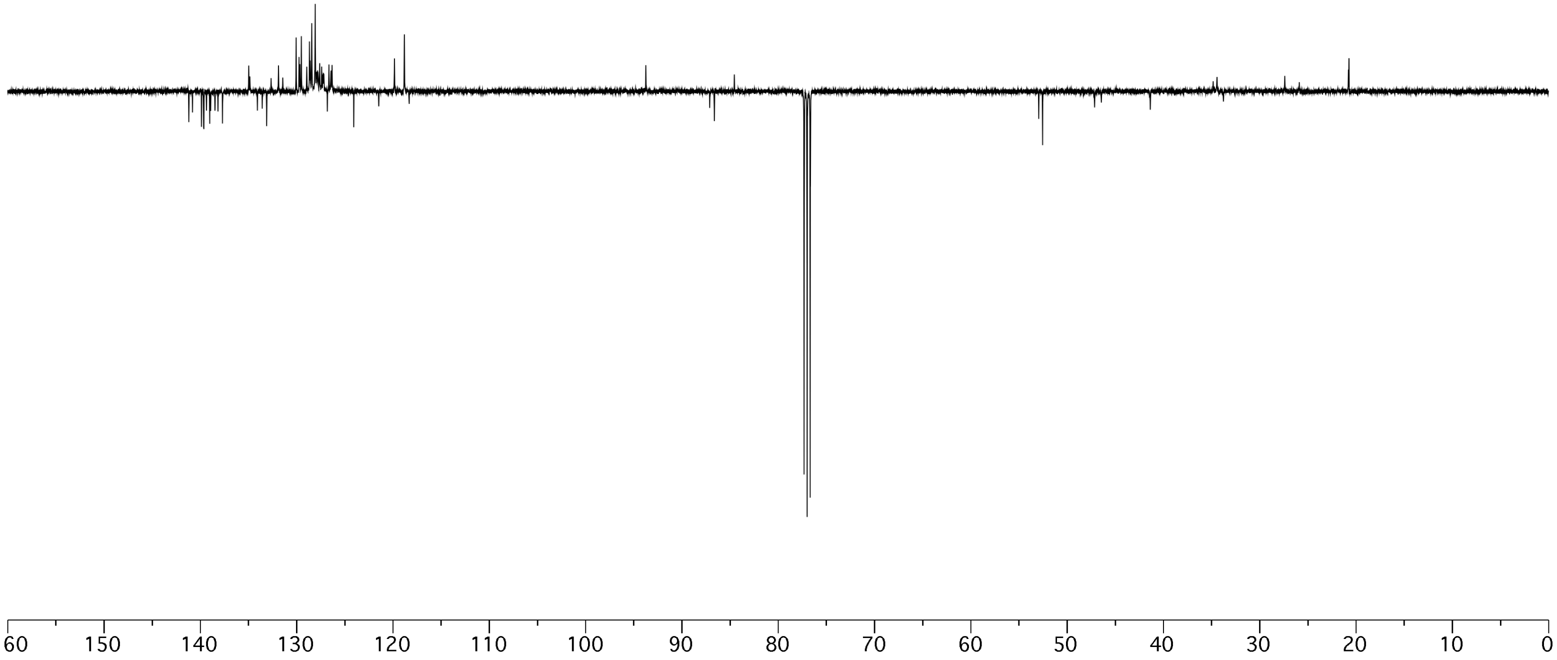

Figure S45. $^{13}C\{^{1}H\}$ APT NMR spectrum of a 1:1.33 mixture of *anti*/*syn*-**3b** (100.8 MHz, $CDCl_3$, –40 ºC)

(The black asterik indicates the signal corresponding to $CDCl_3$)

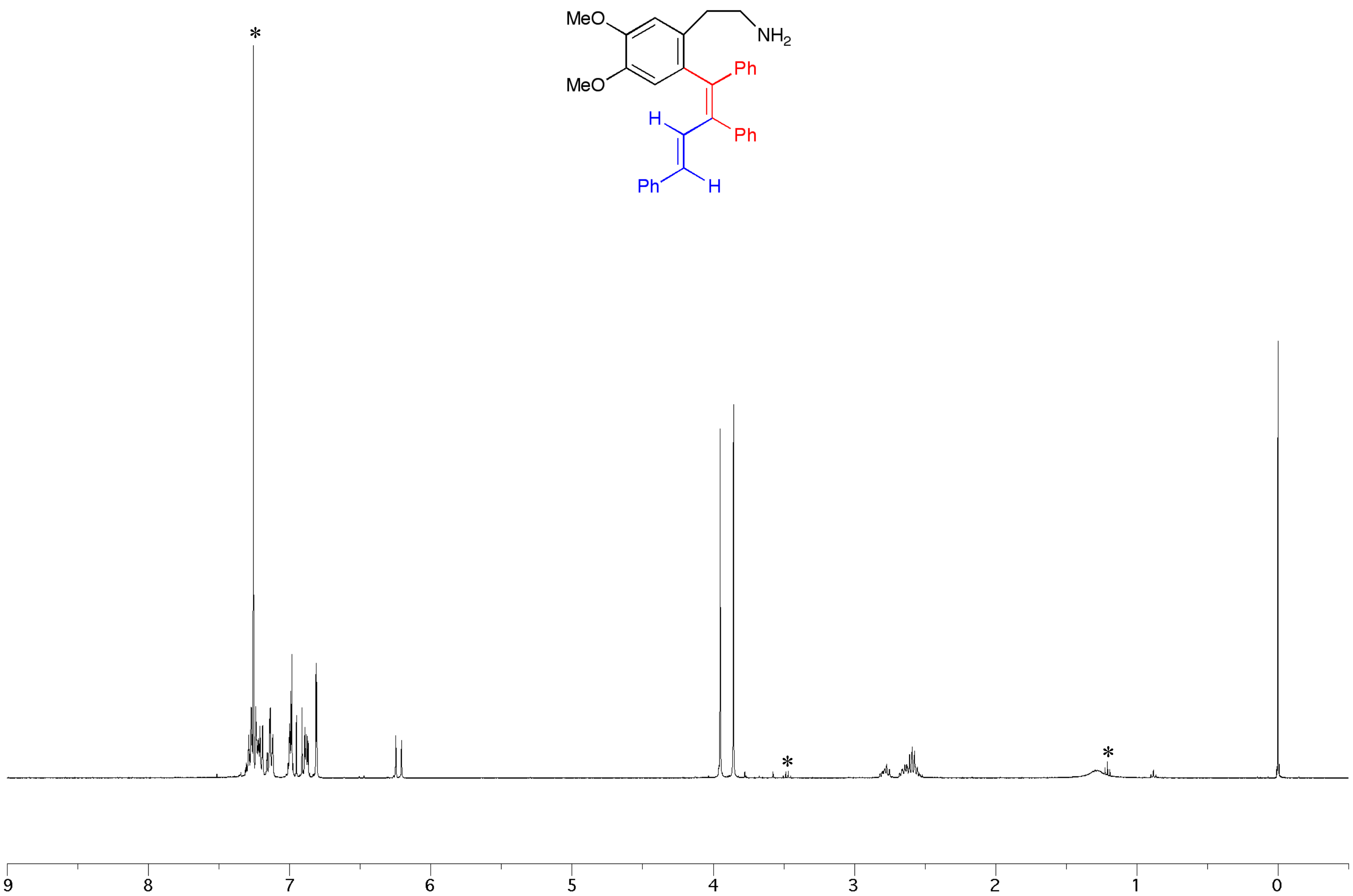

Figure S46. ^{1}H NMR spectrum of **4a**·1/2H_2O (400.9 MHz, $CDCl_3$, 25 ºC)

(From left to right, the asteriks indicate the signals corresponding to $CHCl_3$, and traces of Et_2O)

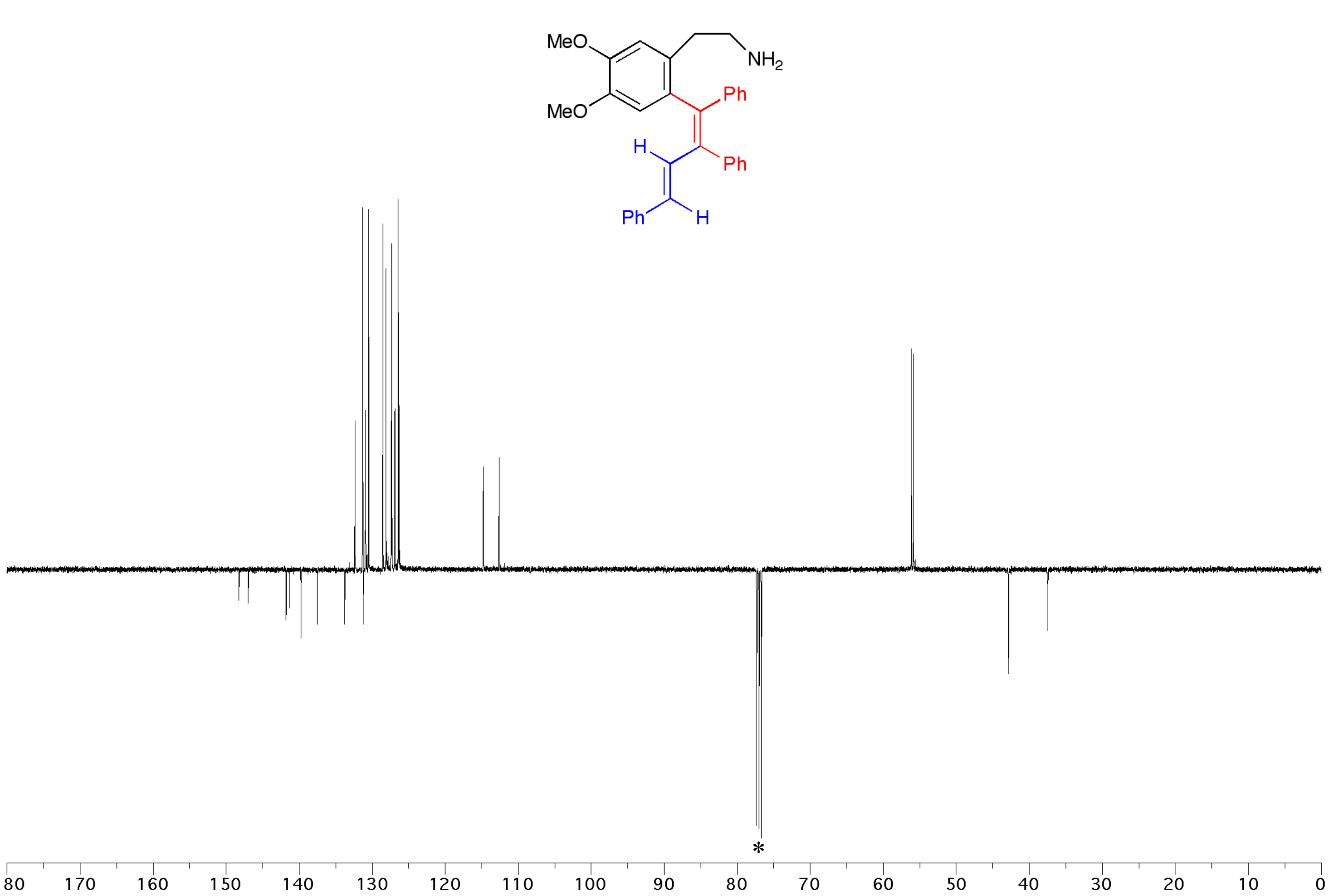

Figure S47. $^{13}C\{^{1}H\}$ APT NMR spectrum of **4a**·1/2H_2O (100.8 MHz, $CDCl_3$, 25 ºC)

(The asterik indicates the signal corresponding to $CDCl_3$)

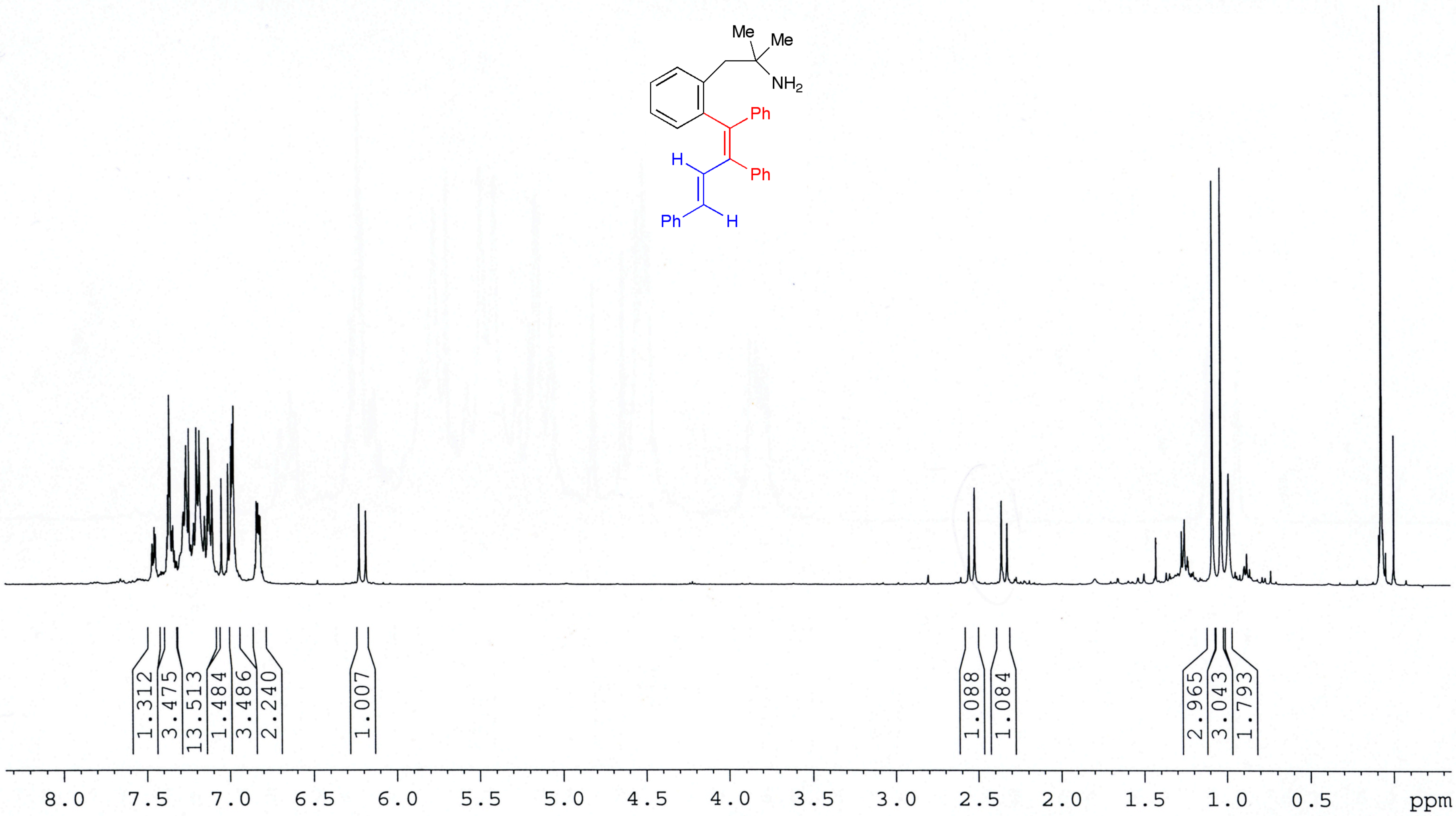

Figure S48. ^{1}H NMR spectrum of crude **4b** (400.9 MHz, $CDCl_3$, 25 ºC)

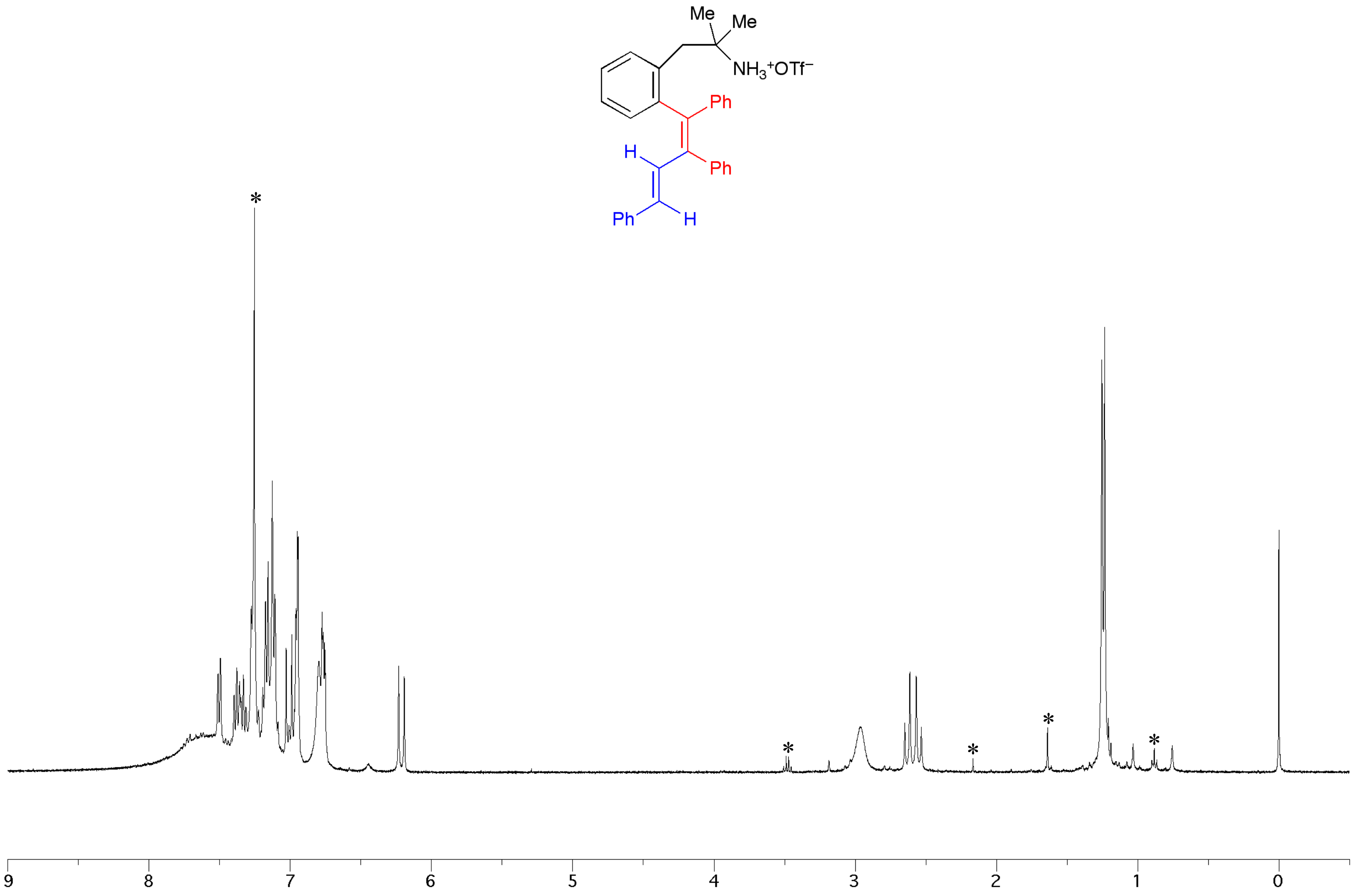

Figure S49. ^{1}H NMR spectrum of **5b**·H_2O (400.9 MHz, $CDCl_3$, 25 ºC)

(From left to right, the asteriks indicate the signals corresponding to $CHCl_3$, and traces of Et_2O, acetone, H_2O, and *n*-pentane)

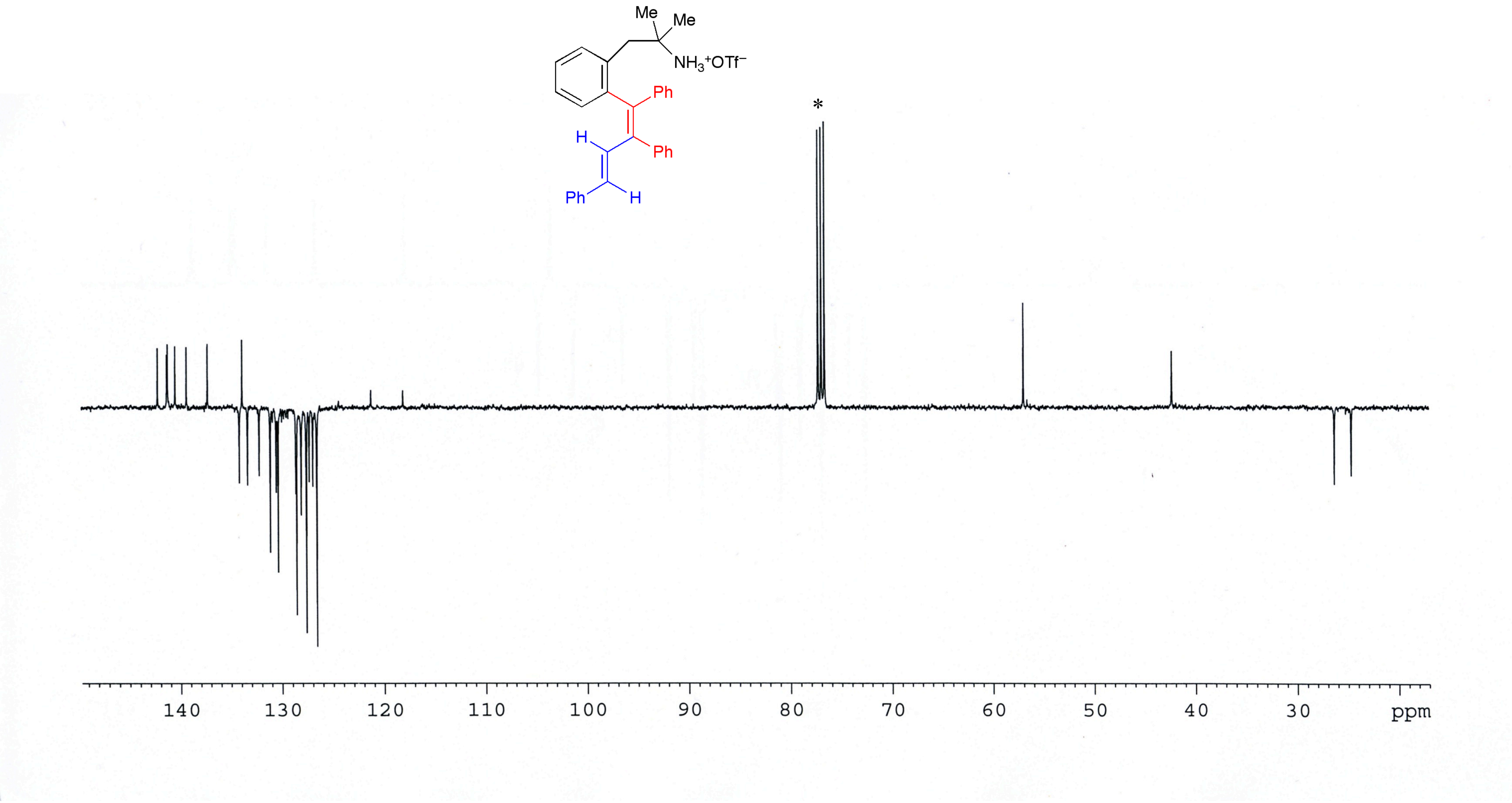

Figure S50. $^{13}C\{^1H\}$ APT NMR spectrum of **5b**·H_2O (100.8 MHz, $CDCl_3$, 25 ºC)

(The asterik indicates the signal corresponding to $CDCl_3$)

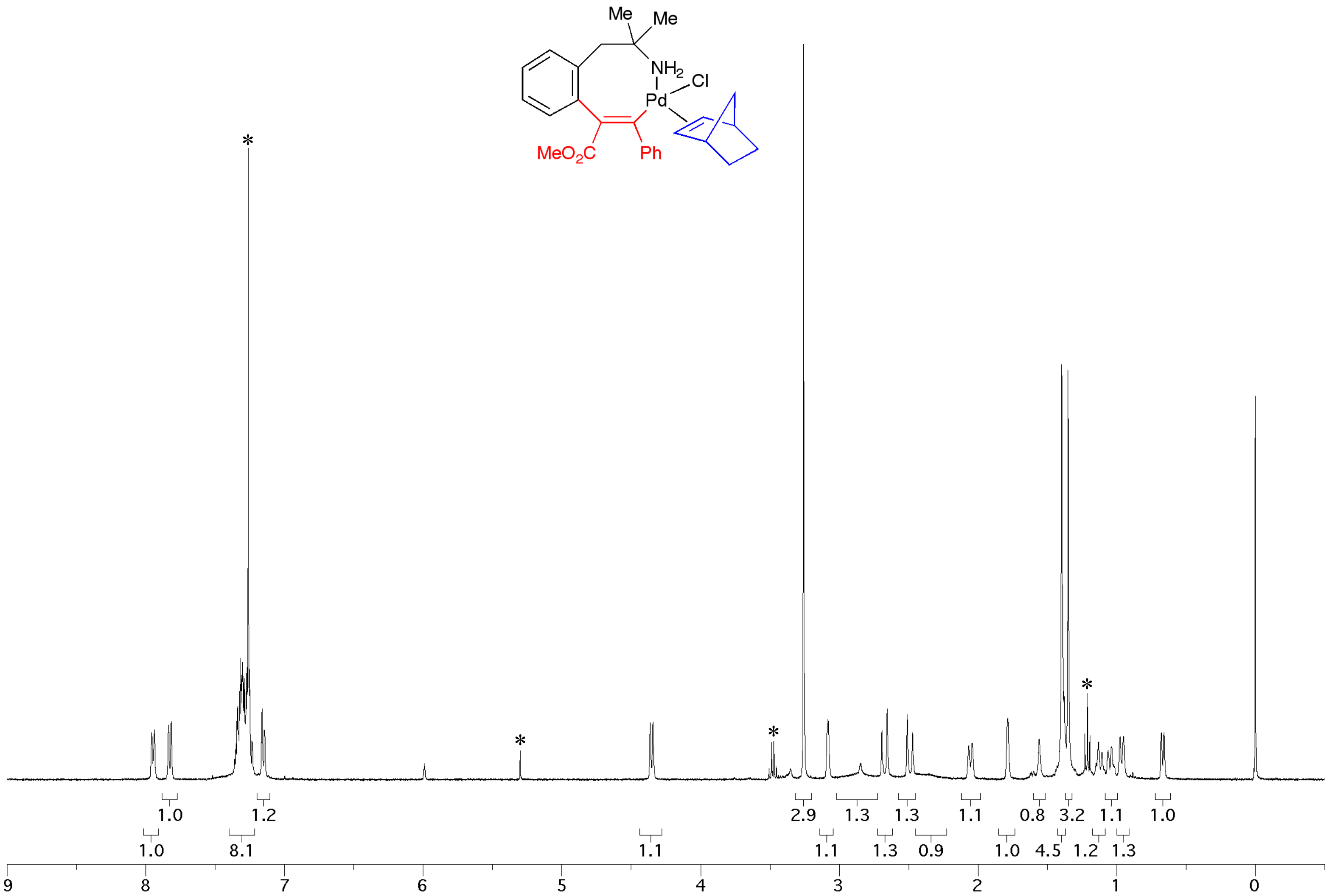

Figure S51. ^{1}H NMR spectrum of **6d** (400.9 MHz, $CDCl_3$, 25 ºC)

(From left to right, the asteriks indicate the signals corresponding to $CHCl_3$, CH_2Cl_2, and Et_2O)

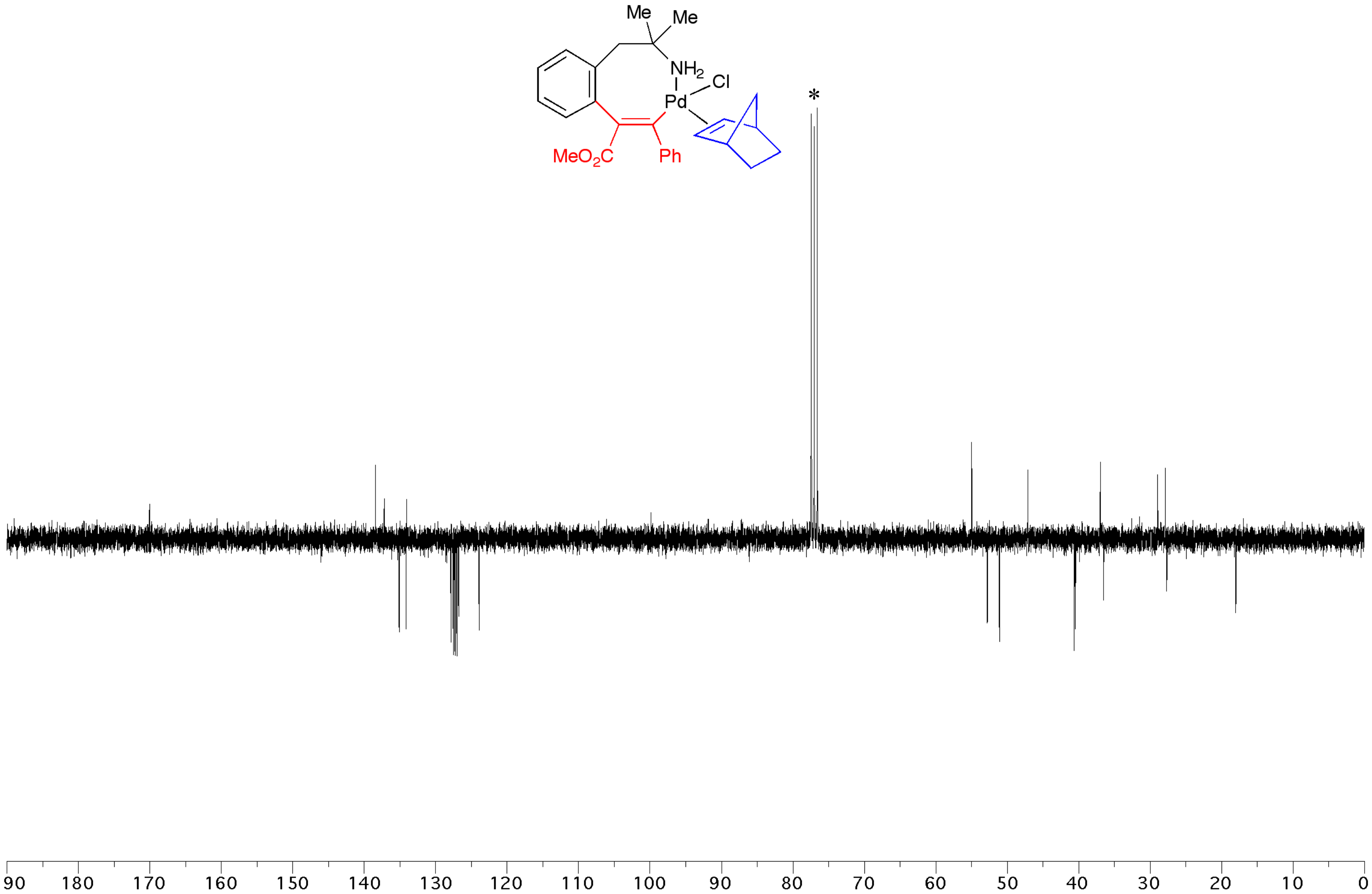

Figure S52. $^{13}C\{^{1}H\}$ APT NMR spectrum of **6d** (75.5 MHz, $CDCl_3$, 25 ºC)

(The asterik indicates the signal corresponding to $CDCl_3$)

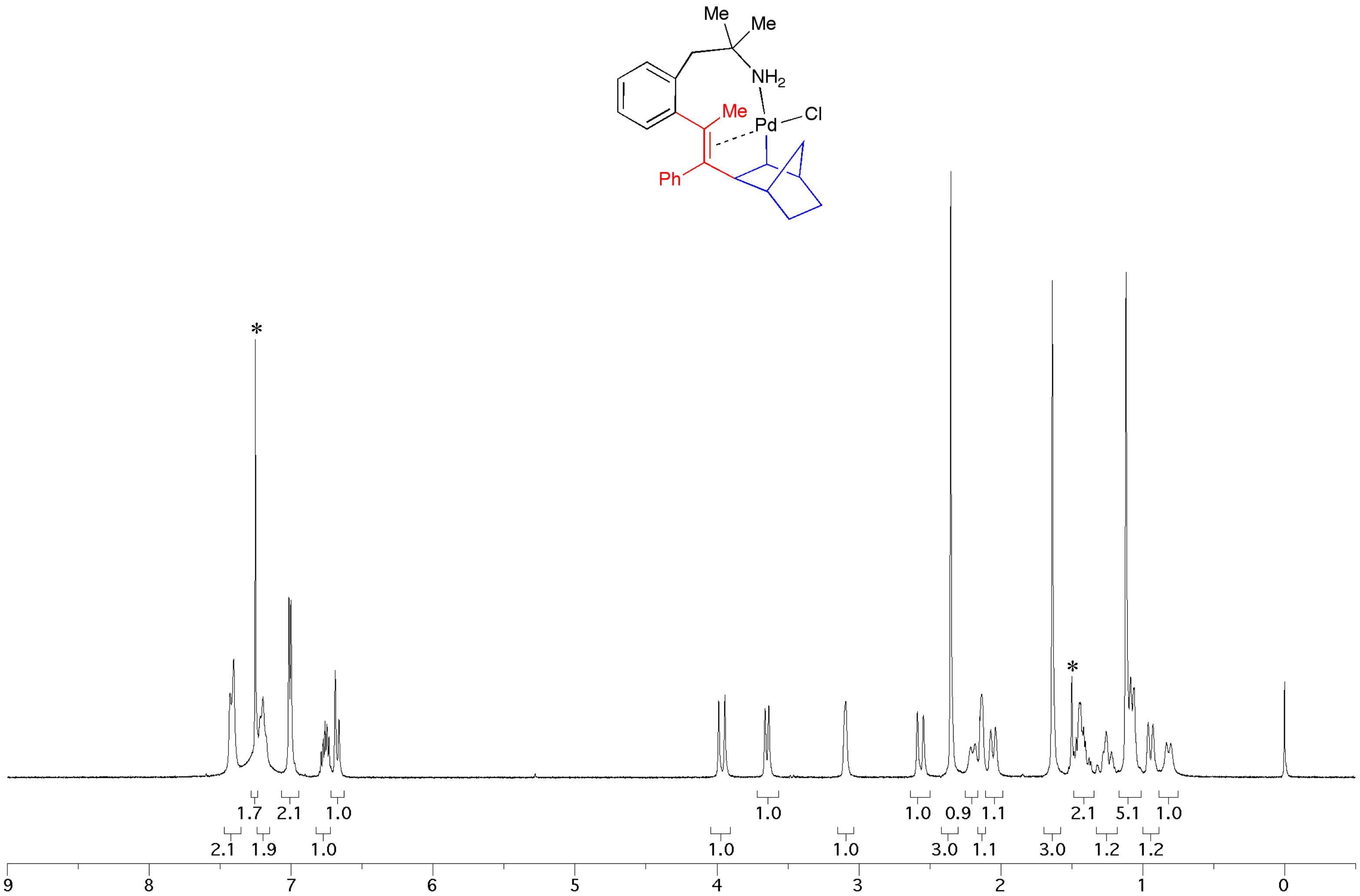

Figure S53. ^{1}H NMR spectrum of **7c**·$CHCl_3$ (300.1 MHz, $CDCl_3$, 25 ºC)

(From left to right, the asteriks indicate the signals corresponding to $CHCl_3$, and H_2O)

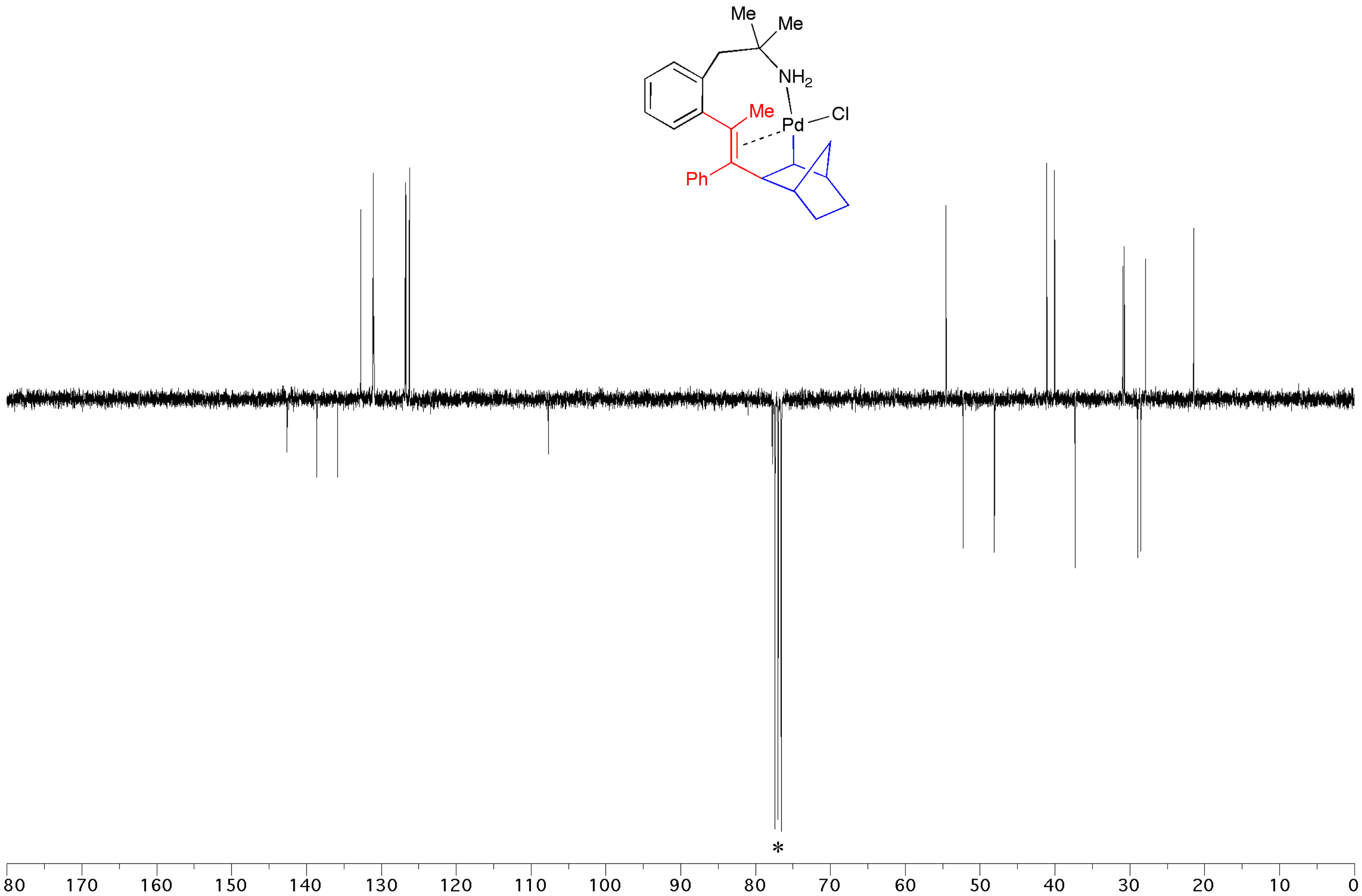

Figure S54. $^{13}C\{^{1}H\}$ APT NMR spectrum of **7c**·$CHCl_3$ (75.5 MHz, $CDCl_3$, 25 ºC)

(The asterik indicates the signal corresponding to $CDCl_3$)

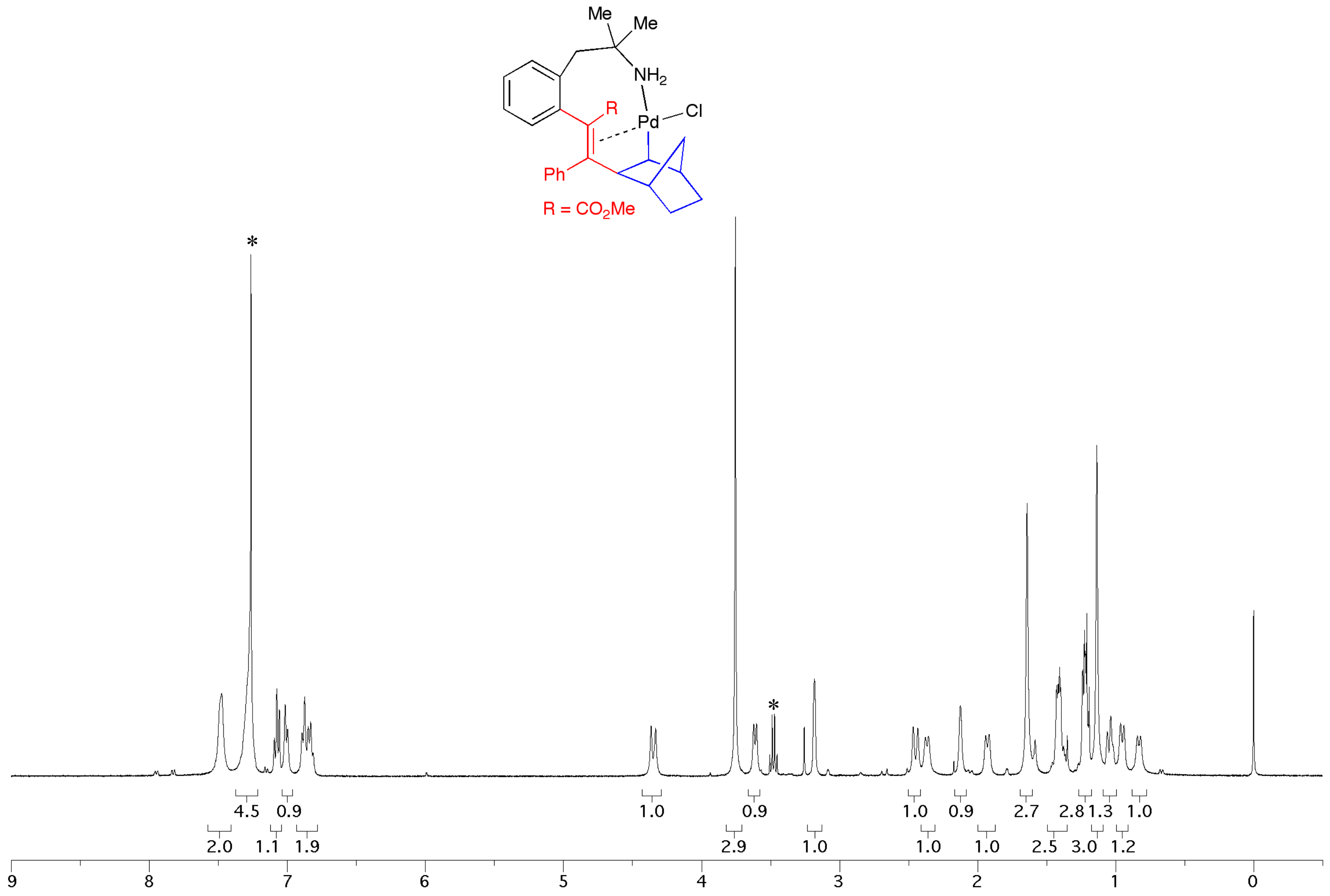

Figure S55. ^{1}H NMR spectrum of **7d**·1/2$CHCl_3$ (400.9 MHz, $CDCl_3$, 25 ºC)

(From left to right, the asteriks indicate the signals corresponding to $CHCl_3$, and Et_2O)

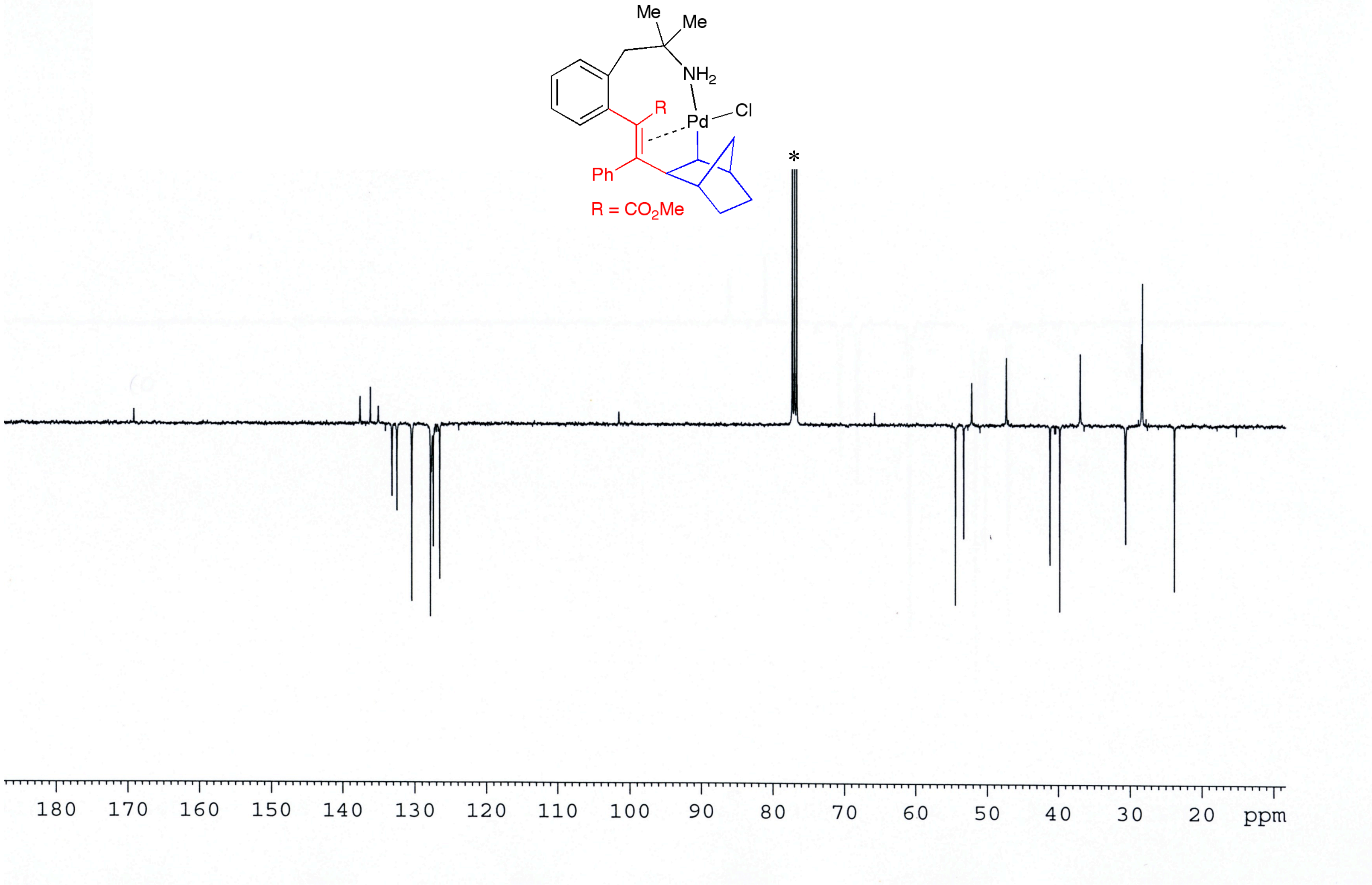

Figure S56. $^{13}C\{^{1}H\}$ APT NMR spectrum of **7d**·1/2CHCl$_3$ (100.8 MHz, CDCl$_3$, 25 ºC)

(The asterik indicates the signal corresponding to CDCl$_3$)

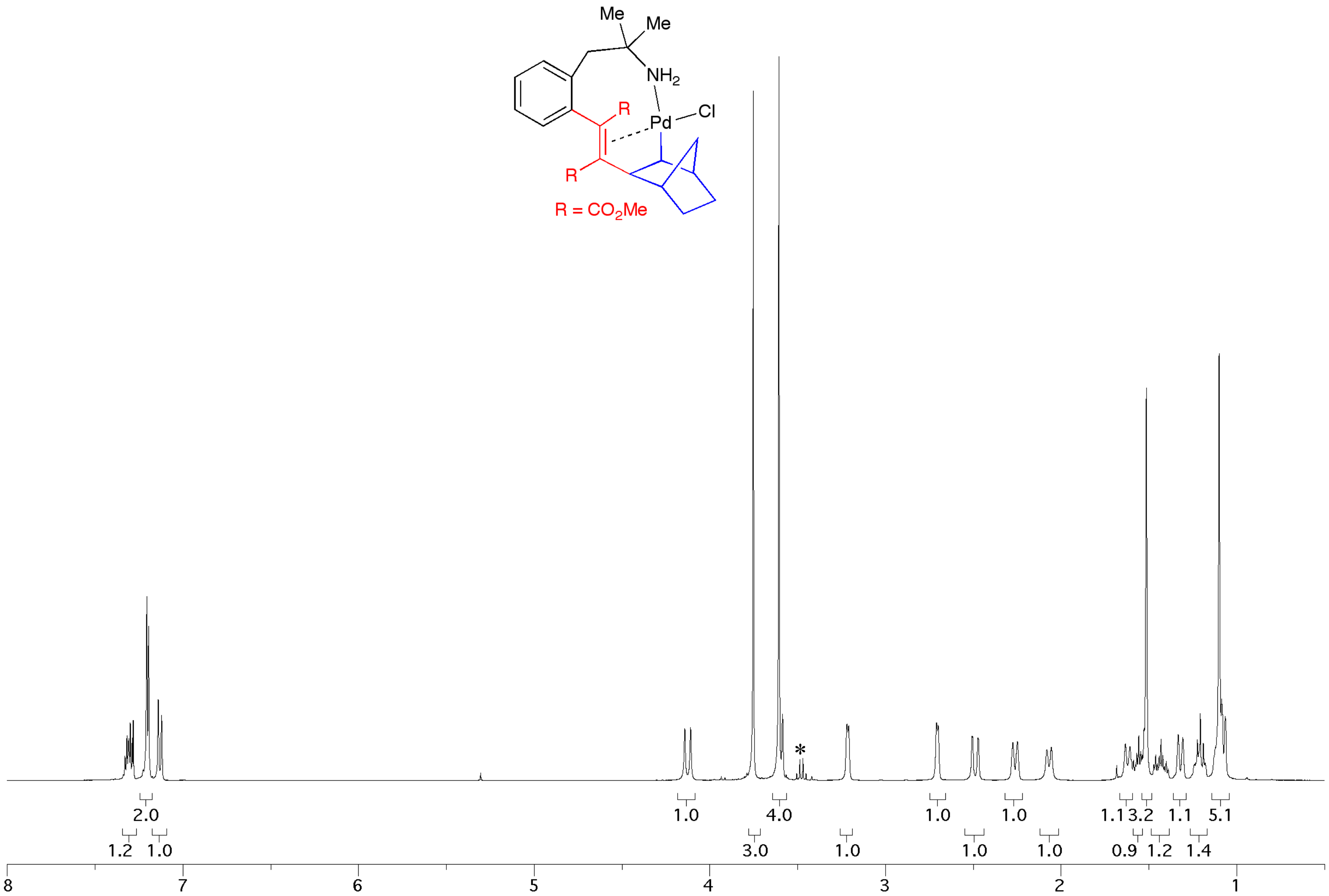

Figure S57. ^{1}H NMR spectrum of **7f** (400.9 MHz, $CDCl_3$, 25 ºC)

(The asterik indicates the signal corresponding to traces of Et_2O)

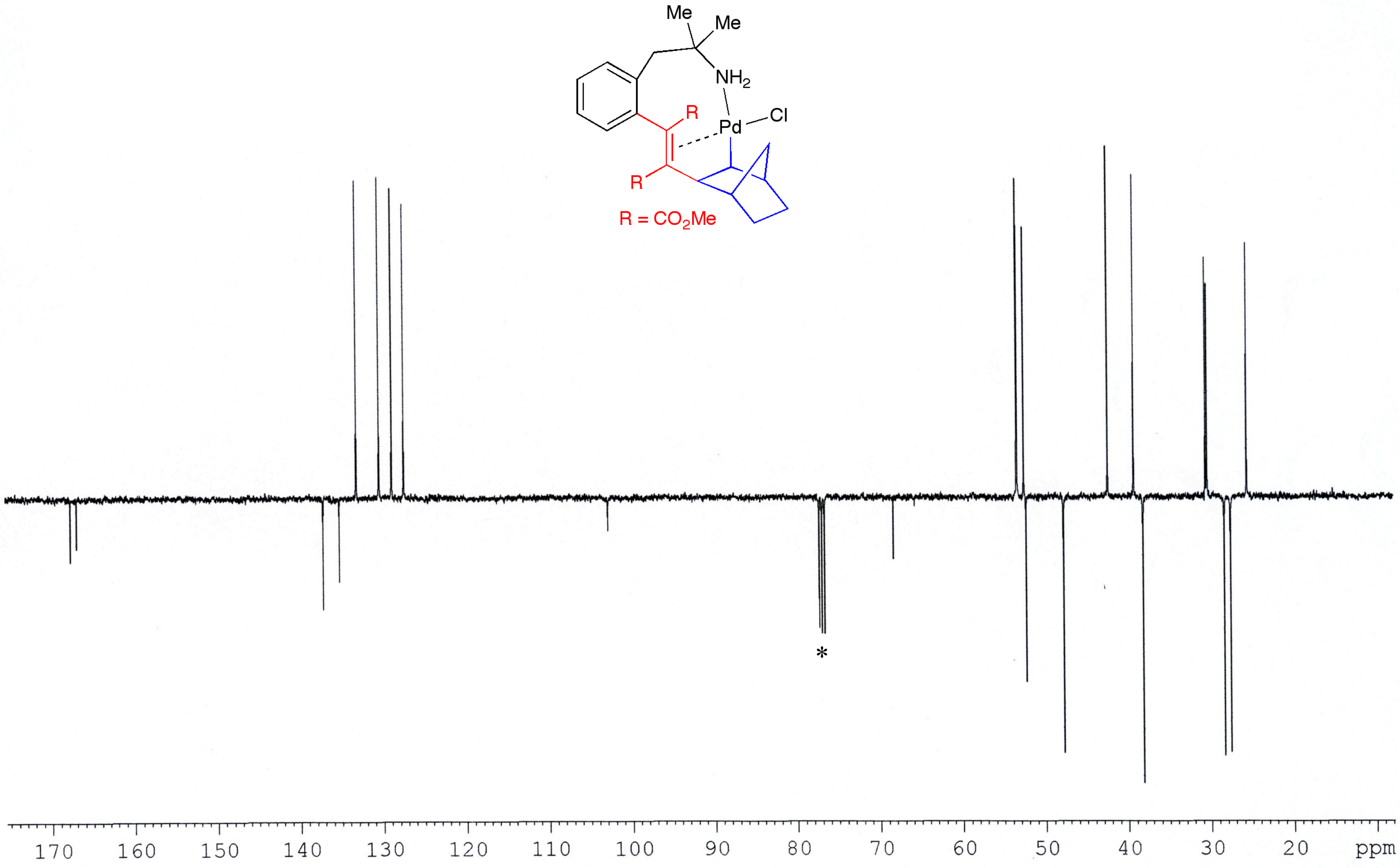

Figure S58. $^{13}C\{^{1}H\}$ APT NMR spectrum of **7f** (100.8 MHz, $CDCl_3$, 25 ºC)

(The asterik indicates the signal corresponding to $CDCl_3$)

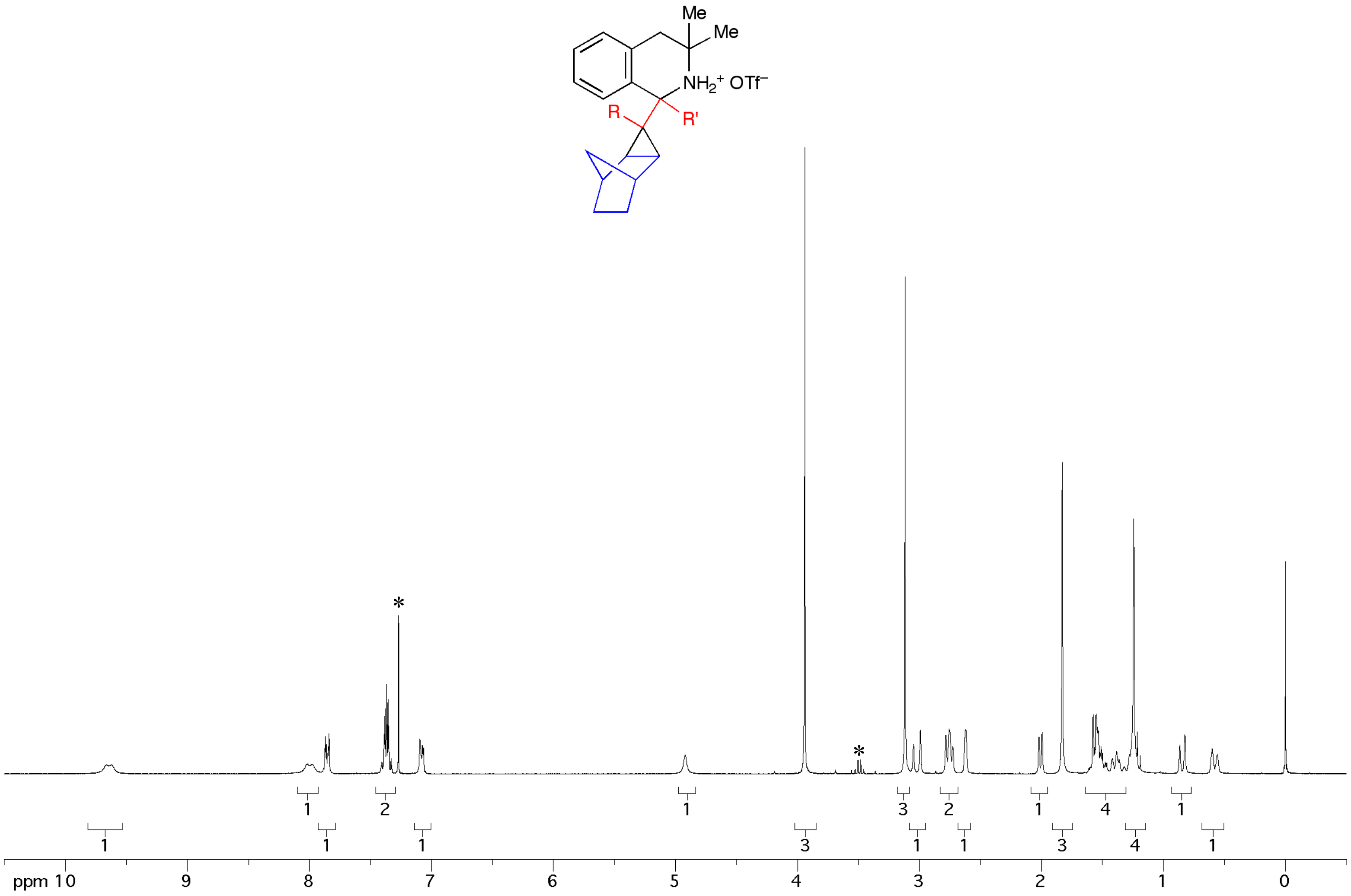

Figure S59. ^{1}H NMR spectrum of **8f** (300.1 MHz, $CDCl_3$, 25 ºC)

(From left to right, the asteriks indicate the signals corresponding to $CHCl_3$ and traces of Et_2O)

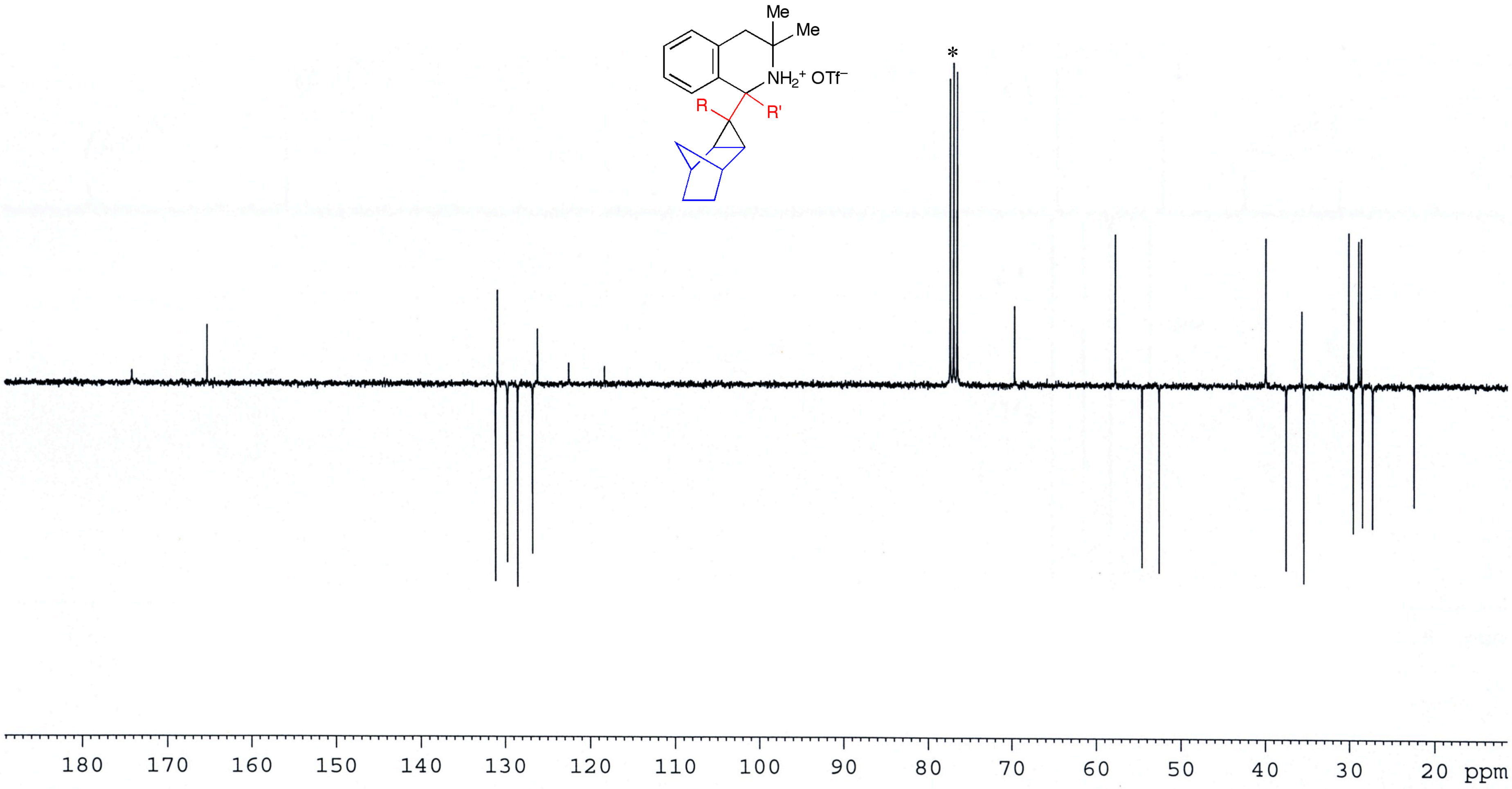

Figure S60. $^{13}C\{^{1}H\}$ APT NMR spectrum of **8f** (75.5 MHz, $CDCl_3$, 25 ºC)

(The asterik indicates the signal corresponding to $CDCl_3$)

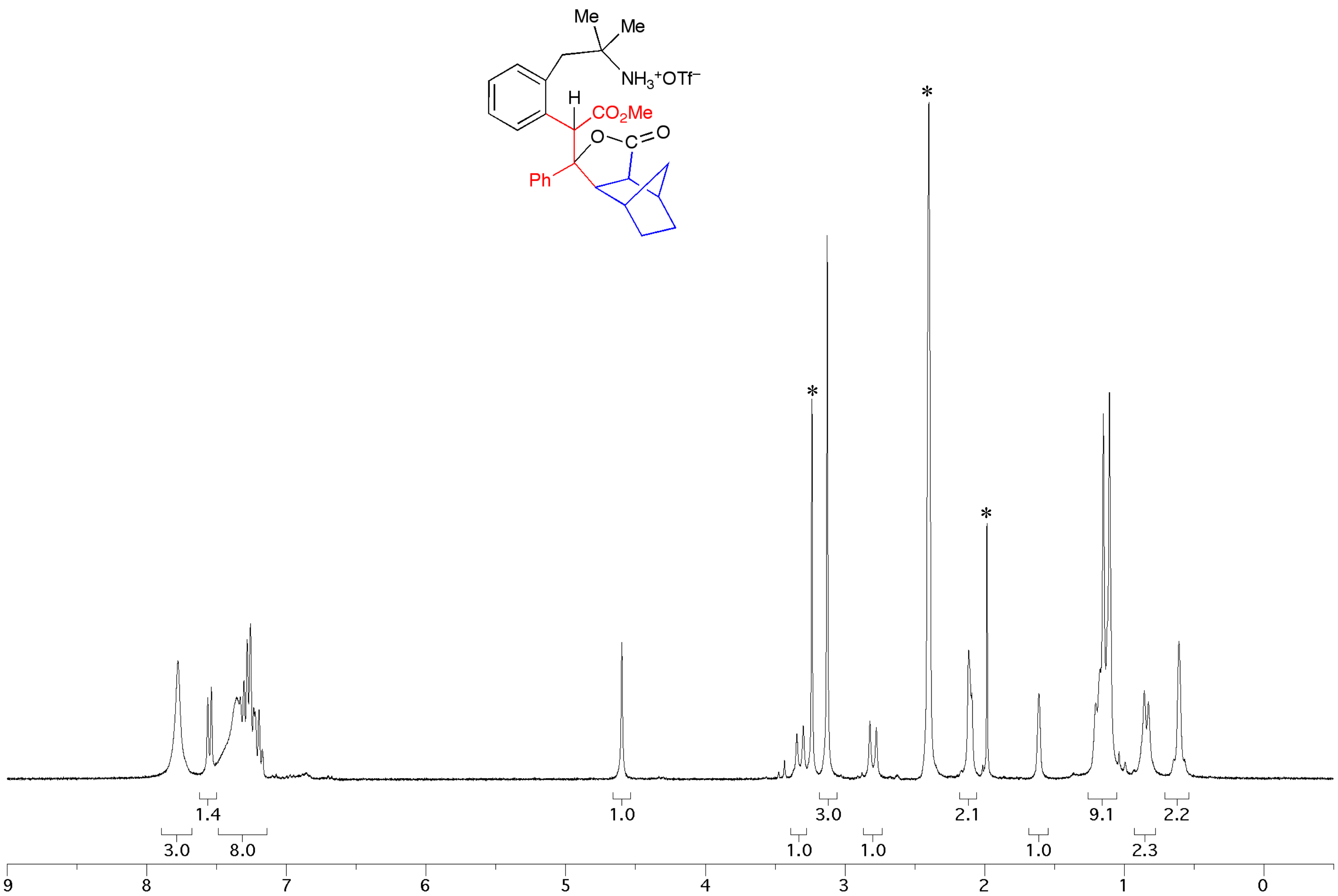

Figure S61. ^{1}H NMR spectrum of **9d** (300.1 MHz, DMSO-d_6, 25 ºC)

(From left to right, the asteriks indicate the signals corresponding to H_2O, deuterated DMSO, and acetone)

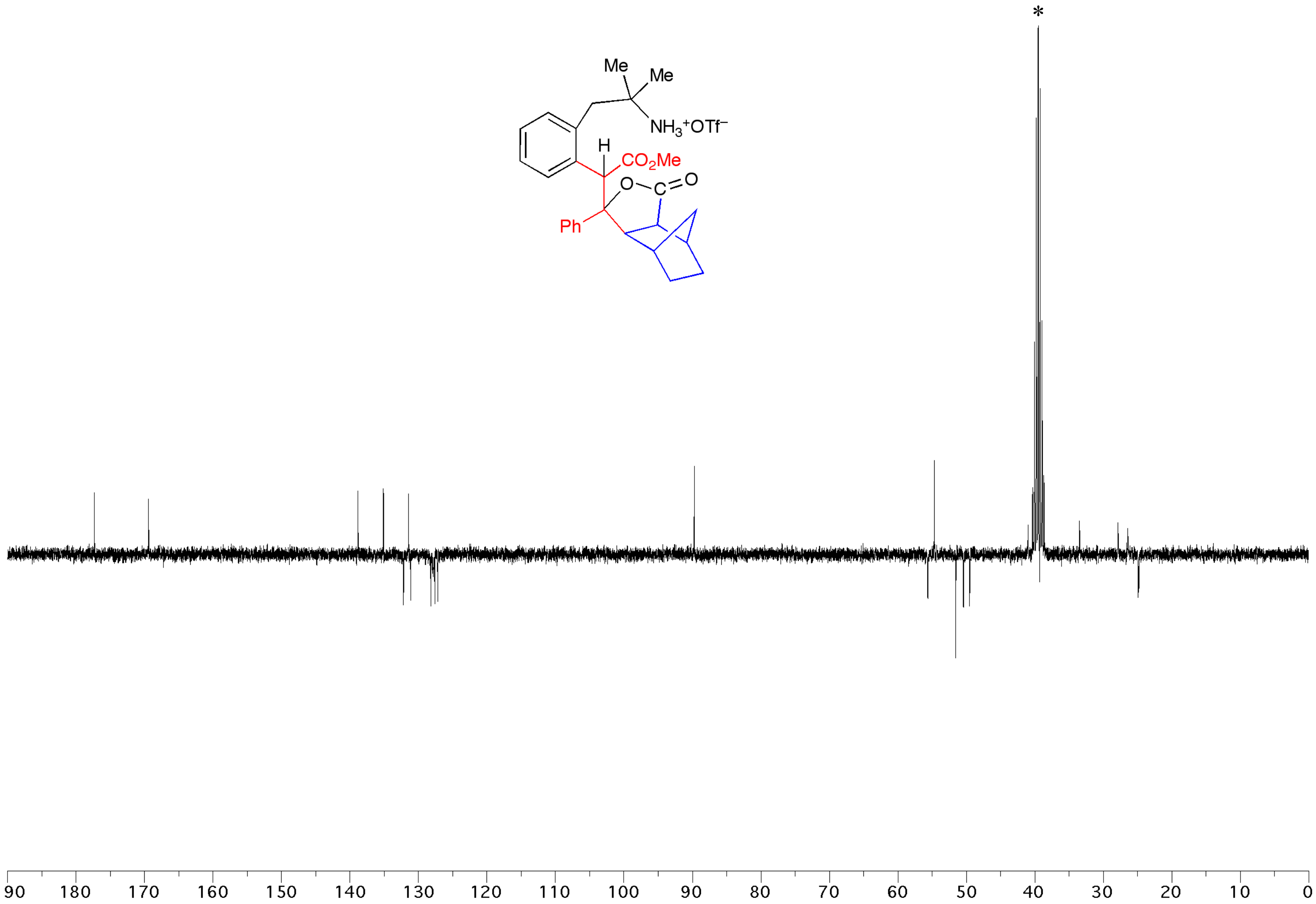

Figure S62. $^{13}C\{^1H\}$ APT NMR spectrum of **9d** (75.5 MHz, DMSO-d_6, 25 ºC)

(The asterik indicates the signal corresponding to DMSO-d_6)

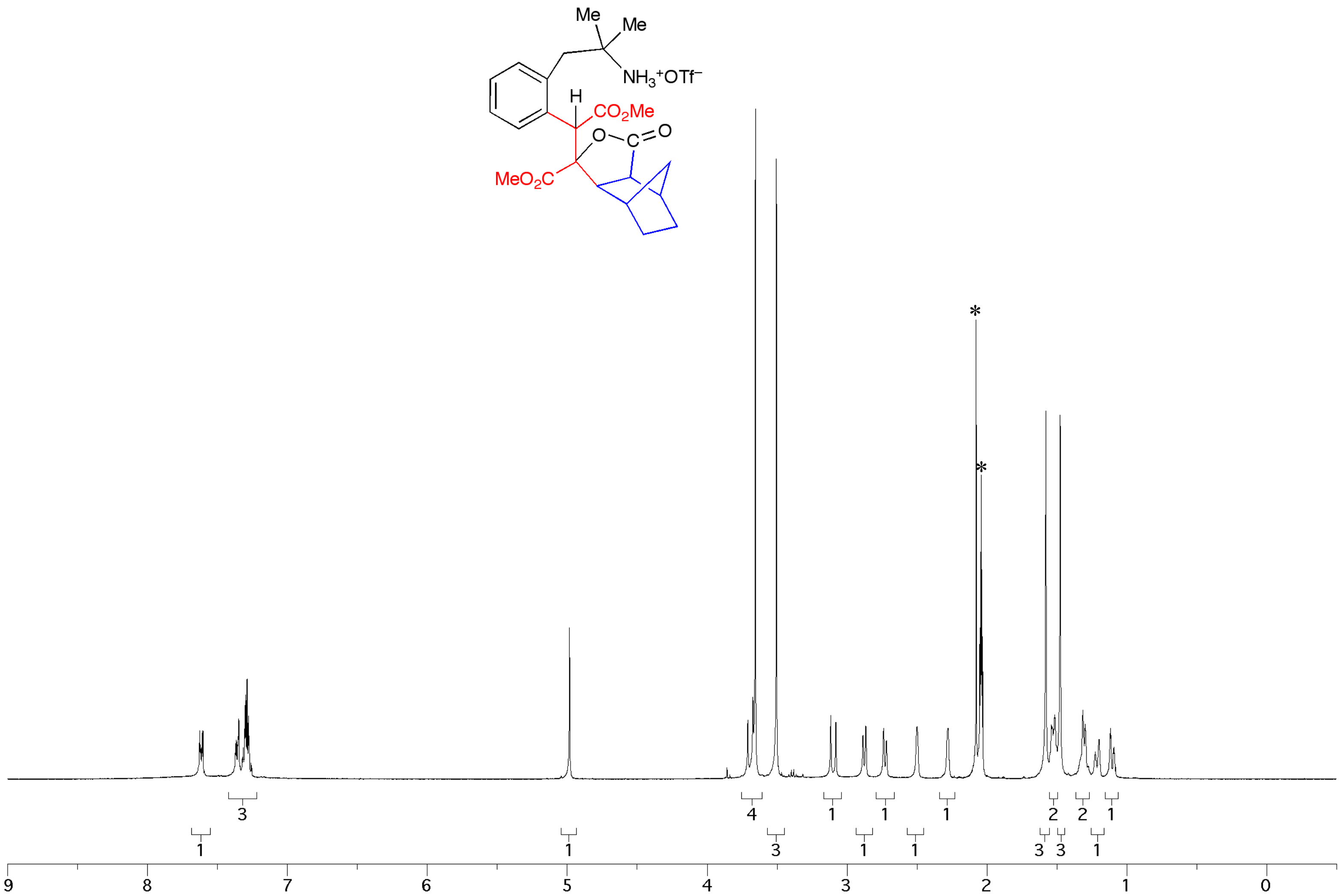

Figure S63. ^{1}H NMR spectrum of **9f** (400.9 MHz, acetone-d_6, 25 ºC)

(From left to right, the asteriks indicate the signals corresponding to deuterated acetone, and acetone)

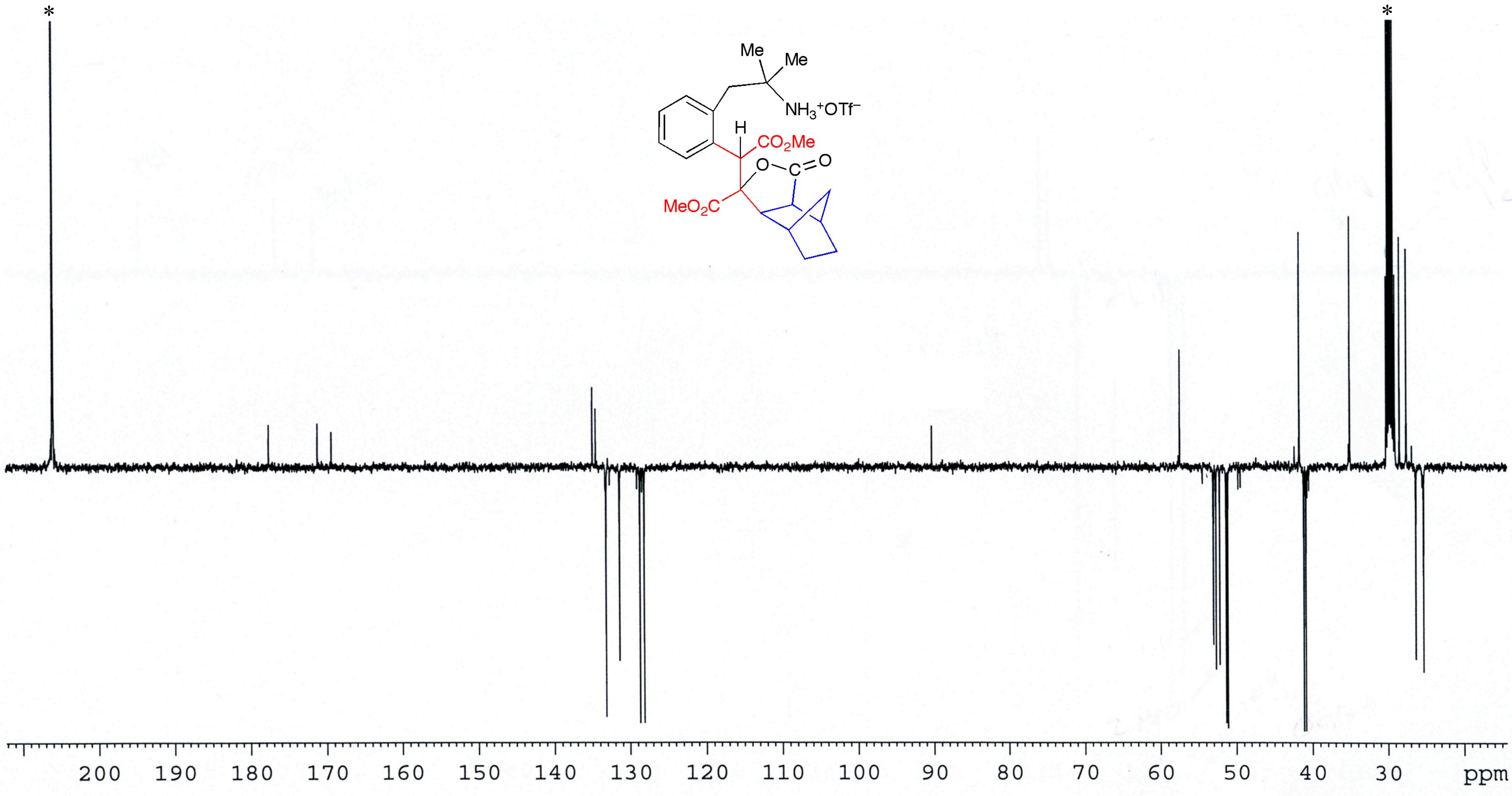

Figure S64. $^{13}C\{^{1}H\}$ APT NMR spectrum of **9f** (100.8 MHz, acetone-d_6, 25 ºC)

(The asteriks indicate the signals corresponding to acetone-d_6)

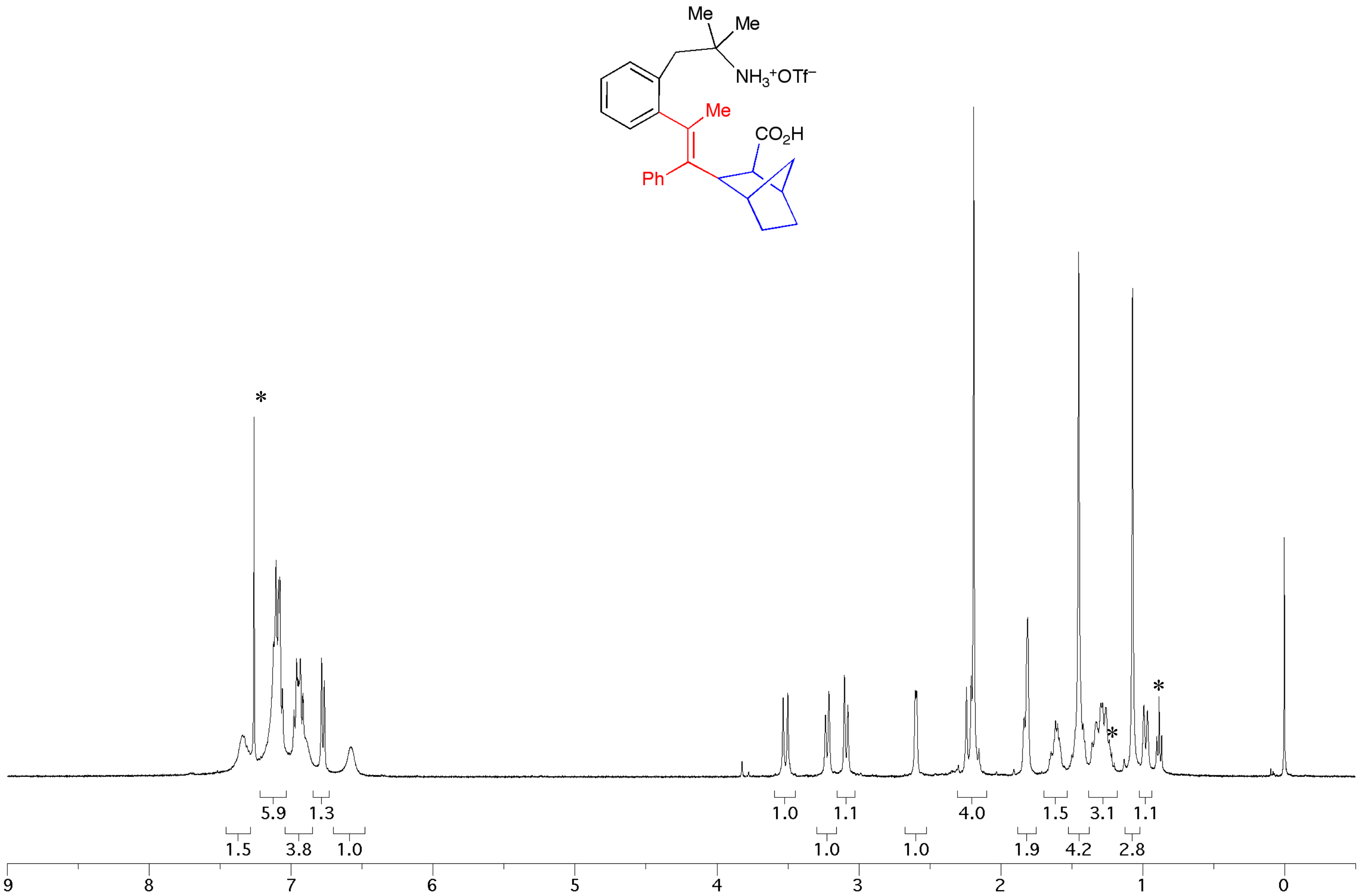

Figure S65. ¹H NMR spectrum of **10c** (400.9 MHz, $CDCl_3$, 25 ºC)

(From left to right, the asteriks indicate the signals corresponding to $CDCl_3$, and traces of *n*-pentane)

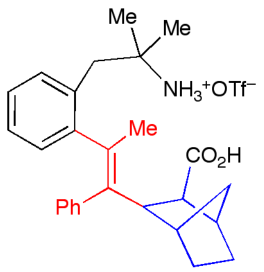

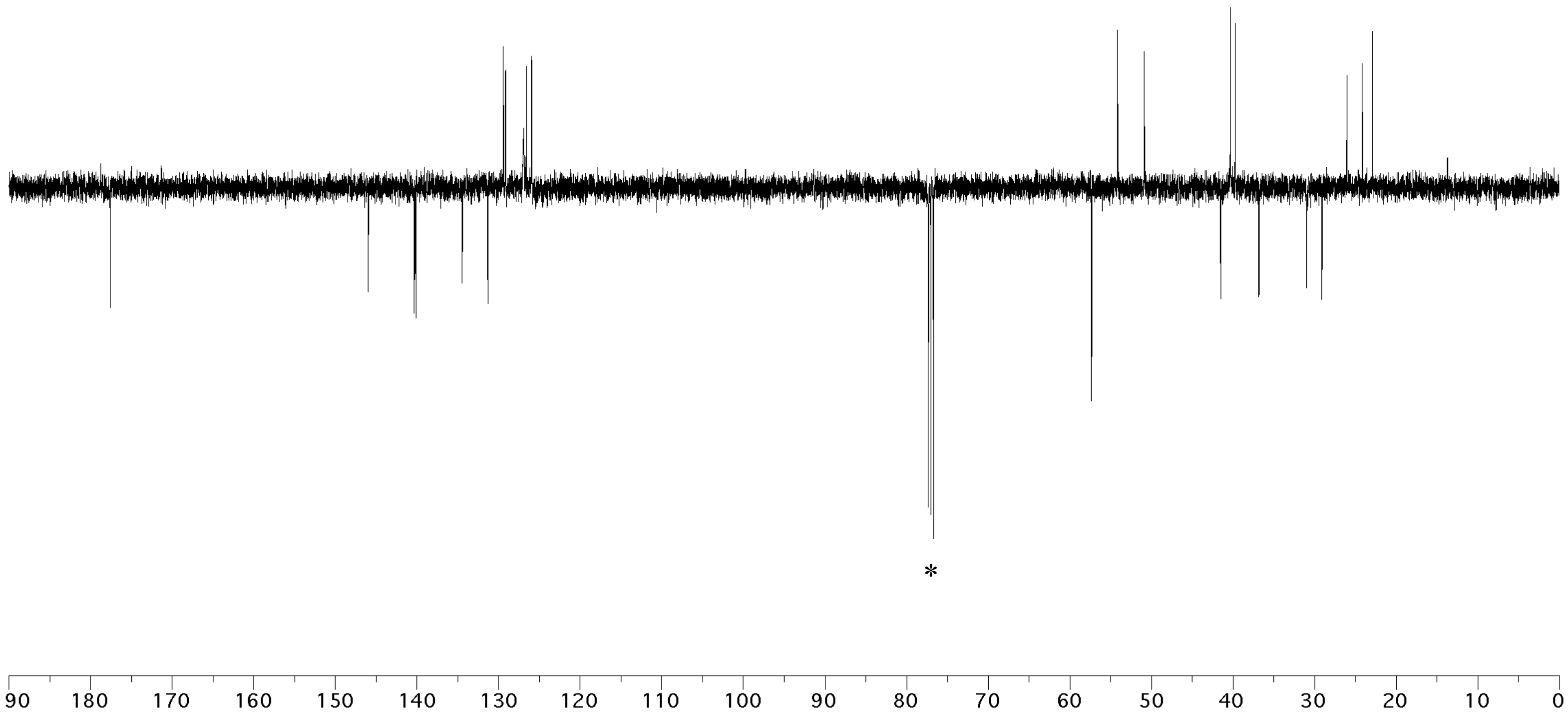

Figure S66. $^{13}C\{^{1}H\}$ APT NMR spectrum of **10c** (100.8 MHz, $CDCl_3$, 25 ºC)

(The asterik indicates the signal corresponding to $CDCl_3$)

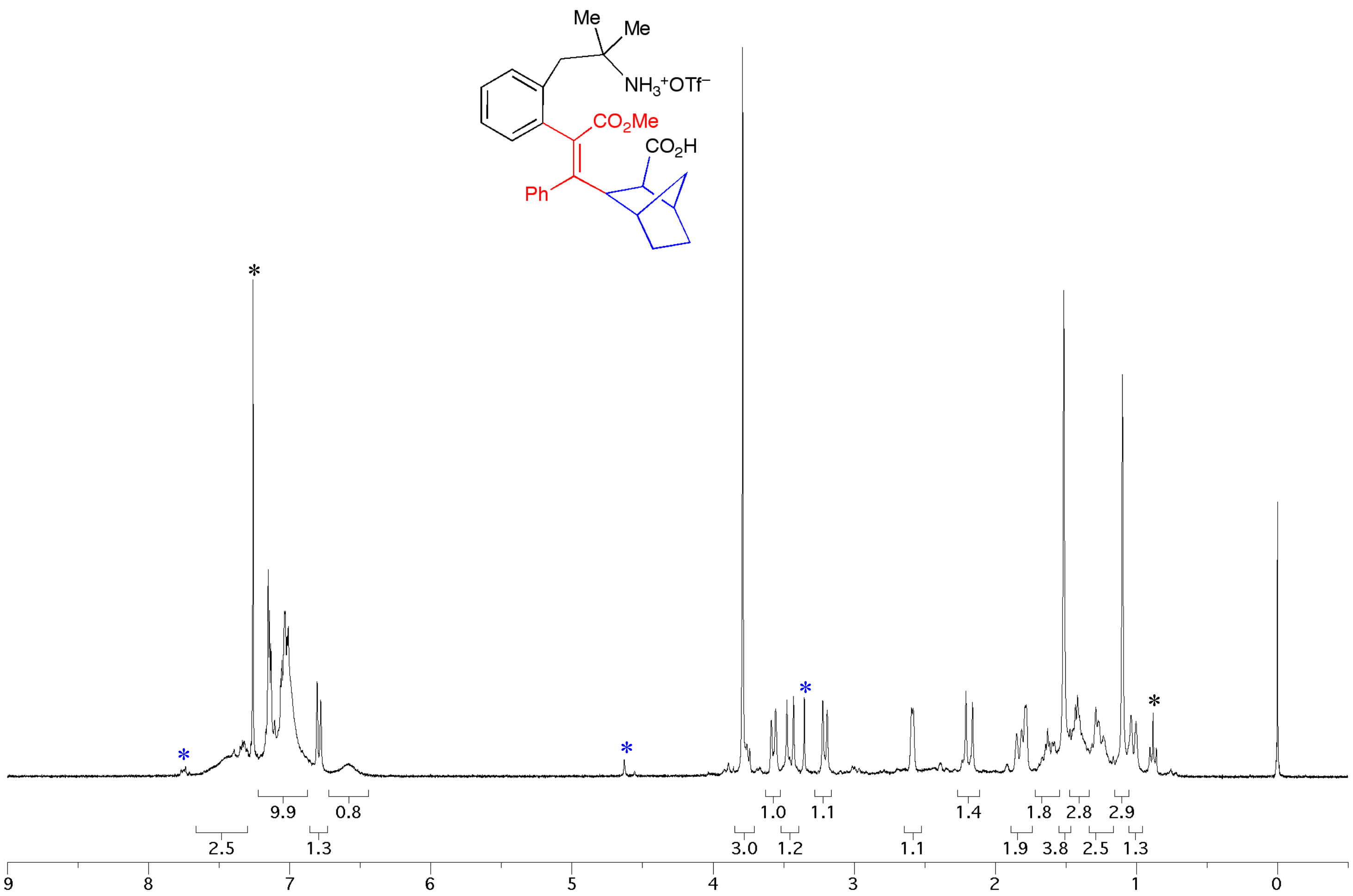

Figure S67. ^{1}H NMR spectrum of **10d** (300.1 MHz, $CDCl_3$, 25 ºC)

(From left to right, the black asteriks indicate the signals corresponding to $CDCl_3$, and traces of *n*-pentane. The blue asteriks correspond to traces of compound **9d**)

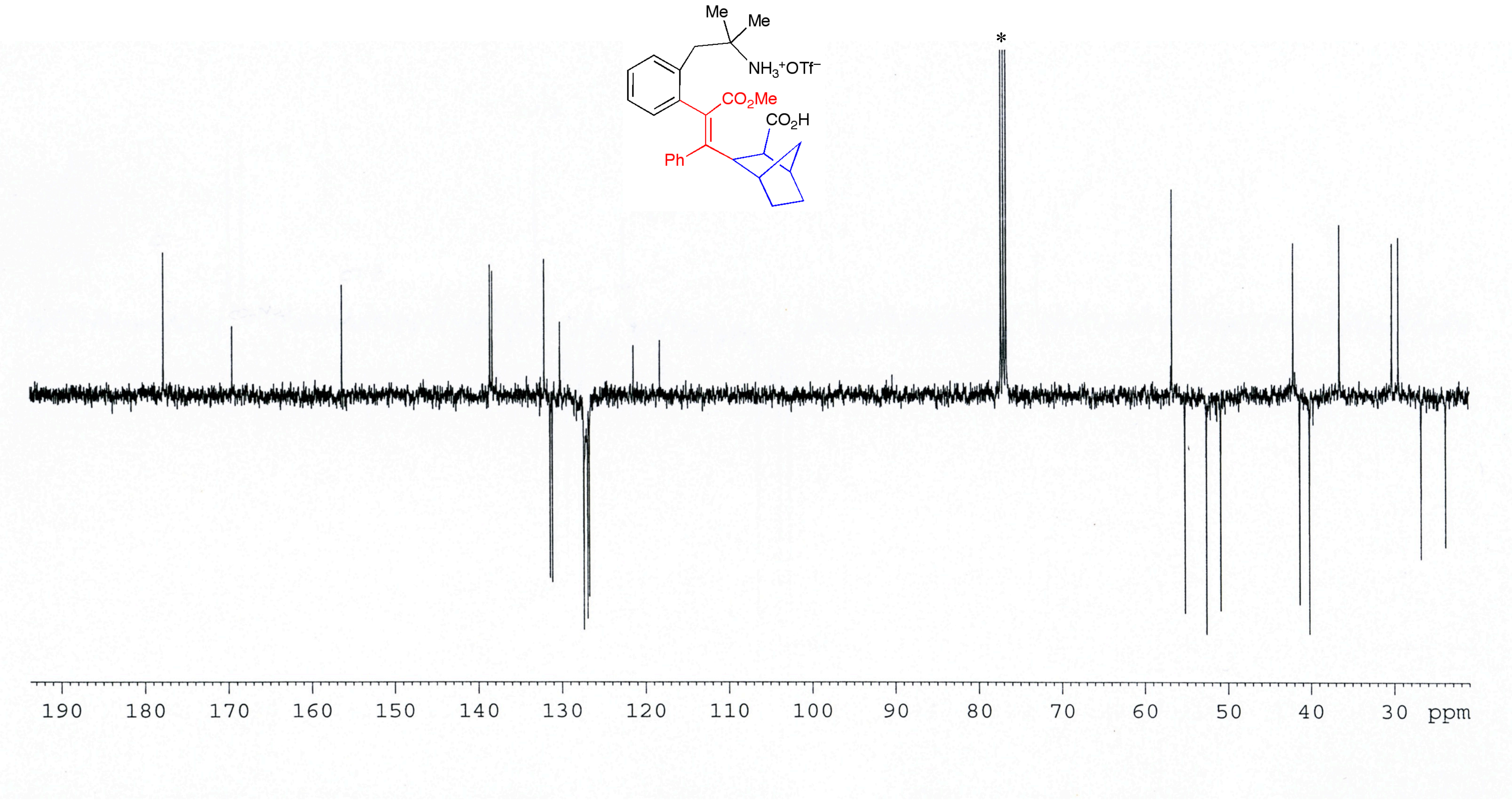

Figure S68. $^{13}C\{^{1}H\}$ APT NMR spectrum of **10d** (100.8 MHz, $CDCl_3$, 25 ºC)

(The asterik indicates the signal corresponding to $CDCl_3$)
